# The Arabidopsis ATP-Binding Cassette E protein ABCE2 is a conserved component of the translation machinery

**DOI:** 10.3389/fpls.2022.1009895

**Published:** 2022-10-17

**Authors:** Carla Navarro-Quiles, Eduardo Mateo-Bonmatí, Héctor Candela, Pedro Robles, Antonio Martínez-Laborda, Yolanda Fernández, Jan Šimura, Karin Ljung, Vicente Rubio, María Rosa Ponce, José Luis Micol

**Affiliations:** ^1^ Instituto de Bioingeniería, Universidad Miguel Hernández, Elche, Spain; ^2^ Área de Genética, Universidad Miguel Hernández, Alicante, Spain; ^3^ Centro Nacional de Biotecnología, CNB-CSIC, Madrid, Spain; ^4^ Umeå Plant Science Centre, Department of Forest Genetics and Plant Physiology, Swedish University of Agricultural Sciences, Umeå, Sweden

**Keywords:** Arabidopsis ABCE2, ribosome recycling, translation machinery, venation pattern, auxin metabolism

## Abstract

ATP-Binding Cassette E (ABCE) proteins dissociate cytoplasmic ribosomes after translation terminates, and contribute to ribosome recycling, thus linking translation termination to initiation. This function has been demonstrated to be essential in animals, fungi, and archaea, but remains unexplored in plants. In most species, ABCE is encoded by a single-copy gene; by contrast, *Arabidopsis thaliana* has two *ABCE* paralogs, of which *ABCE2* seems to conserve the ancestral function. We isolated *apiculata7-1* (*api7-1*), the first viable, hypomorphic allele of *ABCE2*, which has a pleiotropic morphological phenotype reminiscent of mutations affecting ribosome biogenesis factors and ribosomal proteins. We also studied *api7-2*, a null, recessive lethal allele of *ABCE2*. Co-immunoprecipitation experiments showed that ABCE2 physically interacts with components of the translation machinery. An RNA-seq study of the *api7-1* mutant showed increased responses to iron and sulfur starvation. We also found increased transcript levels of genes related to auxin signaling and metabolism. Our results support for the first time a conserved role for ABCE proteins in translation in plants, as previously shown for the animal, fungal, and archaeal lineages. In Arabidopsis, the ABCE2 protein seems important for general growth and vascular development, likely due to an indirect effect through auxin metabolism.

## Introduction

Messenger RNA (mRNA) molecules are decoded for protein synthesis by the complex and ancient translation machinery, formed by the ribosome and different sets of translation factors, which function at different translation phases. Translation initiation factors promote the formation of the 70S/80S initiation complex, and the recognition of the mRNA translation start site (Rodnina, 2018; Shirokikh and Preiss, 2018). Translation elongation factors participate in the binding of aminoacyl-tRNAs to the ribosome, the elongation of the peptides, and the ulterior release of the deacylated tRNA (Dever et al., 2018). Translation termination factors act when the ribosome reaches the translation stop codon and the newly synthesized peptide is released. In this latter phase, the ribosome is dissociated into its 50S/60S and 30S/40S subunits, which are recycled for a new cycle of translation initiation (Hellen, 2018). The ATP-Binding Cassette E (ABCE) proteins are soluble ABC proteins that participate in ribosome recycling and translation initiation, as have been demonstrated for archaea, fungi, and animals, but whose roles in plants remain unexplored (Kashima et al., 2014; Young et al., 2015; Nürenberg-Goloub et al., 2020; Simonetti et al., 2020). Human ABCE1 was first named RNASE L INHIBITOR (RLI) due to its ability to inhibit the activity of RNase L, an enzyme that is only present in mammals (Bisbal et al., 1995).

ABCE proteins contain an iron-sulfur cluster binding domain (FeSD), two nucleotide binding domains (NBD1 and NBD2), and two hinge motifs (Karcher et al., 2005; Barthelme et al., 2007; Karcher et al., 2008). The first hinge motif allows NBD movement to bind and hydrolyze ATP. The second hinge motif and a helix-loop-helix (HLH) mediate the interaction of the ABCE protein with the ribosome after occlusion of two ATP molecules. Once in the ribosome, the ABCE protein displaces its FeSD to split the ribosome, and remains bound to the 30S/40S subunit to prevent a premature recruitment of a 50S/60S subunit during translation initiation. Finally, ATP hydrolysis allows ABCE detachment from the 30S/40S subunit (Barthelme et al., 2011; Becker et al., 2012; Preis et al., 2014; Heuer et al., 2017; Nürenberg-Goloub et al., 2018; Gouridis et al., 2019; Kratzat et al., 2021).

In most genomes, the ABCE subfamily is represented by a single-copy gene, usually named *ABCE1*, whose null alleles are lethal, while hypomorphic alleles result in developmental defects and slow-growth phenotypes (Navarro-Quiles et al., 2018). *Arabidopsis thaliana* (hereafter referred to as Arabidopsis), however, has two *ABCE* paralogs named *ABCE1* and *ABCE2* (Sánchez-Fernández et al., 2001; Verrier et al., 2008). Arabidopsis ABCE2 has been studied for its RNA silencing suppression activity (Sarmiento et al., 2006; Mõttus et al., 2020). In *Cardamine hirsuta*, a close relative of Arabidopsis with compound leaves, only one *ABCE* gene has been identified, *SIMPLE LEAF3* (*SIL3*), which is required for leaflet formation and leaf development. The leaves of homozygotes for the hypomorphic *sil3* mutation are simple and have vascular defects, probably caused by an aberrant auxin transport and homeostasis (Kougioumoutzi et al., 2013).

Here, we report a functional analysis of the Arabidopsis *ABCE2* gene. We studied two recessive alleles of *ABCE2*: the hypomorphic and viable *apiculata7-1* (*api7-1*) allele, and the null and lethal *api7-2* allele. The *api7-1* mutant exhibits the typical morphological phenotype caused by mutations in genes encoding ribosome biogenesis factors and ribosomal proteins, which includes aberrant leaf venation patterns. We found by co-immunoprecipitation that ABCE2 physically interacts with components of the translation machinery, and by RNA-seq that its partial loss of function triggers iron and sulfur deficiency responses that might be related to FeS cluster biogenesis, as well as the upregulation of auxin biosynthesis genes. Our observations strongly suggest a conserved role for plant ABCE proteins in translation, probably through ribosome recycling as previously shown for the animal, fungal, and archaeal lineages.

## Materials and methods

### Plant materials, growth conditions, and crosses

The *Arabidopsis thaliana* (L.) Heynh. wild-type accessions Landsberg *erecta* (L*er*) and Columbia-0 (Col-0), and the *asymmetric leaves1-1* (*as1-1*; N3374; in the Col-1 genetic background) and *as2-1* (N3117; in ER) mutants were initially obtained from the Nottingham Arabidopsis Stock Center (NASC; Nottingham, United Kingdom). We introgressed the *as1-1* and *as2-1* mutations into the Col-0 background by crossing to Col-0 three times. The NASC also provided seeds of the *api7-2* (GABI_509C06; N448798) (Kleinboelting et al., 2012) and *PIN1_pro_:PIN1:GFP DR5_pro_:3XVENUS:N7* (N67931) (Heisler et al., 2005) lines. The *ATHB8_pro_:GUS* line (N296) was kindly provided by Simona Baima (Baima et al., 1995). The *api7-1* line was isolated in the L*er* background after ethyl methanesulfonate (EMS) mutagenesis in our laboratory, and then backcrossed twice to L*er* (Berná et al., 1999). Unless otherwise stated, all the mutants mentioned in this work are homozygous for the mutations indicated. Seed sterilization and sowing, plant culture, crosses, and allelism tests were performed as previously described (Ponce et al., 1998; Berná et al., 1999; Quesada et al., 2000).

### Positional cloning and molecular characterization of *api7* mutant alleles

Genomic DNA was extracted as previously described (Ponce et al., 2006). The *ABCE2* gene was cloned as previously described (Mateo-Bonmatí et al., 2014). First, we mapped the *api7-1* mutation to a 123.5-kb candidate interval containing 30 genes using a mapping population of 273 F_2_ plants derived from an *api7-1* × Col-0 cross, and the primers listed in **Supplementary Table S1**, as previously described (Ponce et al., 1999; Ponce et al., 2006). Then, the whole *api7-1* genome was sequenced by Fasteris (Geneva, Switzerland) using the Illumina HiSeq2000 platform. The bioinformatic analysis of the data was performed as previously described (Mateo-Bonmatí et al., 2014).

Discrimination between the wild-type *ABCE2* and *api7-1* mutant alleles was done by PCR with the api7-1_F/R primers (**Supplementary Table S1**), followed by restriction with *Eco*57I (Thermo Fisher Scientific), as the *api7-1* mutation (CTCCAG→CTTCAG) creates an *Eco*57I restriction site. The presence and position of the *api7-2* T-DNA insertion in the GABI_509C06 line was confirmed by PCR amplification and Sanger sequencing, respectively, using gene-specific primers and the o8409 primer for the GABI-Kat T-DNA (**Supplementary Table S1**).

### Gene constructs and plant transformation

All inserts were PCR amplified from Col-0 genomic DNA using Phusion High Fidelity DNA Polymerase (Thermo Fisher Scientific) and primers that contained *att*B sites at their 5′ ends (**Supplementary Table S1**). PCR products were purified using an Illustra GFX PCR DNA and Gel Band Purification Kit (Cytiva), and then cloned into the pGEM-T Easy221 vector, transferred to *Escherichia coli* DH5α, and subcloned into the pEarleyGate 101, pMDC83, or pMDC107 destination vectors (Curtis and Grossniklaus, 2003; Earley et al., 2006) as previously described (Mateo-Bonmatí et al., 2018).

All constructs were transferred to electrocompetent *Agrobacterium tumefaciens* GV3101 (C58C1 Rif^R^) cells, which were used to transform L*er* or *api7-1* plants by the floral dip method (Clough and Bent, 1998). Putative transgenic plants were selected on plates supplemented with 15 µg·ml^−1^ hygromycin B (Thermo Fisher Scientific, Invitrogen).

To obtain the GSRhino-TAP-tagged ABCE2 fusion, a pGEM-T Easy221 vector harboring the *ABCE2* coding sequence, together with the pEN-L4-2-R1 and pEN-R2-GSRhinotag-L3 entry vectors were subcloned into the pKCTAP destination vector as previously described (Van Leene et al., 2015). Transformation of Arabidopsis cell cultures was performed as previously described (Van Leene et al., 2015).

### Phenotypic analysis and morphometry

Photographs were taken with a Nikon SMZ1500 stereomicroscope equipped with a Nikon DXM1200F digital camera. For larger specimens, four to five partial images from the same plant were taken and merged using the Photomerge tool of Adobe Photoshop CS3 software. For rosette size, rosette silhouettes were drawn on the screen of a Cintiq 18SX Interactive Pen Display (Wacom) using Adobe Photoshop CS3, and their sizes were measured with the NIS Elements AR 3.1 image analysis package (Nikon). Root length was measured per triplicate from photographs with the Freehand line tool from Fiji software (https://imagej.net/ImageJ) (Schindelin et al., 2012). Shoot length was measured *in vivo* with a millimeter ruler, from the soil to the apex of the main shoot. Chlorophyll *a* and *b* and carotenoids were extracted and spectrophotometrically determined as previously described (Wellburn, 1994; Micol-Ponce et al., 2020), and normalized to the amount of collected tissue.

### Differential interference contrast and bright-field microscopy, andGUS analyses

For differential interference contrast (DIC) and bright-field microscopy, all samples were cleared, mounted, and photographed as previously described (Candela et al., 1999). Micrographs of venation patterns, and leaf primordia expressing *ATHB8_pro_:GUS* were taken under bright field with a Nikon D-Eclipse C1 confocal microscope equipped with a Nikon DS-Ri1 camera, using the NIS-Elements AR 3.1 software (Nikon). Diagrams from leaf cells and venation patterns, and morphometric analysis of leaf cells were obtained as previously described (Pérez-Pérez et al., 2011; Mateo-Bonmatí et al., 2018). For venation pattern morphometry, the phenoVein (http://www.plant-image-analysis.org) (Bühler et al., 2015) software was used. Leaf lamina circularity was calculated as 4 · *π* · *area/perimeter* ^2^. Lamina area and perimeter were measured on diagrams from the leaf lamina with the Fiji Wand tool. GUS assays were performed as previously described (Robles et al., 2010).

### Confocal microscopy and fluorescence quantification

Confocal laser scanning microscopy images were obtained using a Nikon D-Eclipse C1 confocal microscope equipped with a Nikon DS-Ri1 camera and processed with the operator software EZ-C1 (Nikon). Visualization of the fluorescent proteins and dyes was performed on primary roots mounted with deionized water on glass slides. Fluorescent proteins, 4′,6-diamidino-2-phenylindole (DAPI), and propidium iodide were visualized as described in **Supplementary Table S2**. For fluorescence quantification of the *PIN1_pro_:PIN1:GFP* and *DR5_pro_:3XVENUS:N7* protein products, wild-type and *api7-1* seedlings homozygous for these transgenes were grown vertically on the same Petri dishes under identical conditions for 5 days. Image acquisition was performed using a 40**×** objective with a 0.75 numerical aperture. The dwell time was set at 2.16 and 1.68 µs for PIN1:GFP and 3XVENUS:N7, respectively. Four images were acquired and averaged per optical section. Five optical sections encompassing 4 µm from the innermost root layers were photographed. Acquired images (.ids files) were used to generate flat images (.tiff files) with Fiji, by stacking the optical sections from the fluorescent protein channel. Fluorescence quantification was performed using the Fiji Mean gray value measurement.

### RNA isolation, cDNA synthesis, and quantitative PCR

Samples for RNA extraction were collected on ice and immediately frozen for storage at −80°C until use. RNA was isolated using TRIzol (Thermo Fisher Scientific, Invitrogen). Removal of contaminating DNA, cDNA synthesis, and quantitative PCR (qPCR) were performed as previously described (Mateo-Bonmatí et al., 2018). The qPCR was performed as follows: 2 min at 50°C, 10 min at 95°C, followed by 41 cycles of 15 s at 95°C and 1 min at 60°C, and a final step of 15 s at 95°C, and *ACTIN2* (*ACT2*) was used as an internal control for relative expression analysis (Moschopoulos et al., 2012). Three biological replicates, each with three technical replicates, were analyzed per genotype. Relative quantification of gene expression data was performed using the comparative C_T_ method (2^−ΔΔC^_T_) (Schmittgen and Livak, 2008).

### RNA-seq analysis

Total RNA was isolated from 100 mg of L*er* and *api7-1* rosettes collected 14 days after stratification (das) using TRIzol. RNA concentration and quality were assessed using a 2100 Bioanalyzer (Agilent Genomics) with an RNA 600 Nano Kit (Agilent Technologies) as previously described (Mateo-Bonmatí et al., 2018). Three biological replicates per genotype, with more than 14 µg of total RNA per sample, and an RNA integrity number (RIN) higher than 7, were sent to Novogene (Cambridge, United Kingdom) for massive parallel sequencing. Sequencing libraries were generated using NEBNext Ultra RNA Library Prep Kit for Illumina (New England Biolabs) and fed into a NovaSeq 6000 Illumina platform with a S4 Flow Cell type, which produced paired-end reads of 150 bp (**Supplementary Table S3**). Read mapping to the Arabidopsis genome (TAIR10) using the 2.0.5 version of HISAT2 (Kim et al., 2019), with default parameters, and the identification of differentially expressed genes between L*er* and *api7-1* with the 1.22.2 version of DESeq2 R package (Love et al., 2014) were performed by Novogene. Genes with a *P* < 0.05 adjusted with the Benjamini and Hochberg’s method, and with a fold change > 1.5 were considered differentially expressed. The GO enrichment analysis of the differentially expressed genes was performed with the online tool DAVID (https://david.ncifcrf.gov/home.jsp) (Huang et al., 2009a; Huang et al., 2009b).

### Indol-3-acetic acid metabolite profiling

Shoots, whole roots, and primary root tips (3 mm approximately) were collected 9 das from vertically grown seedlings. These samples were rapidly weighed and frozen in liquid nitrogen. Extraction and purification of the targeted compounds (anthranilate, Ant; tryptophan, Trp; indole-3-acetonitrile, IAN; indol-3-acetic acid, IAA; glycosylated IAA, IAA-glc; IAA conjugated to aspartate, IAA-Asp, and glutamate, IAA-Glu; 2-oxindole-3-acetic acid, oxIAA; oxIAA-glucoside, oxIAA-glc) were performed as previously described (Novák et al., 2012; Mateo-Bonmatí et al., 2021). Ultra-high performance liquid chromatography followed by MS/MS (UHPLC-MS/MS) analysis was performed as previously described (Pěnčík et al., 2018).

### Co-immunoprecipitation assay

For protein extraction, 700 mg of whole *api7-1 35S_pro_:ABCE2:YFP* seedlings were collected 10 das per biological replicate. The tissue was crosslinked with 1× phosphate-saline buffer containing 1% (v/v) formaldehyde as previously described (Poza-Viejo et al., 2019). For protein extraction, the tissue was ground to a fine powder with liquid nitrogen and then resuspended in a lysis buffer (50 mM Tris-HCl, pH 7.5; 0.1% [v/v] IGEPAL CA-630 [Sigma-Aldrich]; 2 mM phenylmethylsulfonyl fluoride [PMSF; Sigma-Aldrich]; 150 mM NaCl; and a cOmplete protease inhibitor cocktail tablet [Sigma-Aldrich]) using a vortexer. After incubation on ice for 10 min, the samples were centrifuged at 4°C and the supernatants were used as protein extracts. Co-immunoprecipitation was performed with the µMACS GFP Isolation Kit (Milteny Biotec) using protein extracts from three biological replicates. The immunoprecipitation of the ABCE2:YFP fusion protein was checked by western blotting using an anti-GFP-HRP antibody (Milteny Biotec), and the WesternSure chemiluminiscent substrate on a C-DiGit Blot Scanner (LI·COR).

The co-immunoprecipitates were analyzed by liquid chromatography followed by electrospray ionization and tandem mass spectrometry (LC-ESI-MS/MS) at the Centro Nacional de Biotecnología (CNB) Proteomics facility (Madrid, Spain). Tandem mass spectra were searched against Araport11 using the MASCOT search engine (Matrix Science, http://www.matrixscience.com/). Peptide sequences identified with a false discovery rate (FDR) < 1% were considered statistically valid. Proteins identified with at least 2 peptides without overlapping sequences (unique peptides) in at least 2 biological replicates (namely, at least 4 peptides) were considered identified with high confidence. To search for potential ABCE2:YFP interactors, proteins whose subcellular localization was not predicted to be cytoplasmic by SUBA4 (https://suba.live/) (Hooper et al., 2014; Hooper et al., 2017) were discarded, with the exception of At2g20830, which is predicted to localize to mitochondria (see Results). To further discard potential false positive interactions, all the proteins identified in three other co-immunoprecipitations of GFP-fused proteins performed in our laboratory under identical conditions to that of ABCE2:YFP, but functionally unrelated, were used to create a subtract list. Proteins identified in ABCE2:YFP samples with at least twice the number of peptides assigned to the same protein in the subtract list were considered enriched. The rest of the proteins, which contained a more similar number of peptides between the ABCE2:YFP list and the subtract list, were considered false positives and discarded. In addition, there were few proteins that were solely identified in ABCE2:YFP samples.

### Tandem affinity purification assay

TAP assay of the GSRhino-TAP-tagged ABCE2 fusion from Arabidopsis cell suspension cultures was performed as previously described (Van Leene et al., 2015; García-León et al., 2018). Proteins were identified by nano LC-MS/MS at the CNB. Tandem mass spectra were searched against Araport11 using the MASCOT search engine. Proteins identified with at least 1 unique peptide with a MASCOT score higher than 25 (*P* < 0.05) were considered to be valid. Proteins identified with at least 1 unique peptide in the 2 biological replicates or 2 unique peptides in 1 biological replicate were considered identified with high confidence. We discarded as putative ABCE2 interactors those proteins that were not predicted to be cytoplasmic by SUBA4.

### Bioinformatic analyses

The identity and similarity values between conserved proteins were obtained from global pairwise sequence alignments performed with EMBOSS Needle (https://www.ebi.ac.uk/Tools/psa/emboss_needle/) (Madeira et al., 2019). The multiple sequence alignment of ABCE orthologs was obtained with Clustal Omega (https://www.ebi.ac.uk/Tools/msa/clustalo/) (Madeira et al., 2019).

A TBLASTN search was performed to identify *ABCE* genes within eudicots (taxid:71240) against the sequences contained in the Nucleotide collection database at the National Center for Biotechnology Information BLASTP server (NCBI; https://blast.ncbi.nlm.nih.gov/Blast.cgi) (Altschul et al., 1997) using *Arabidopsis thaliana* ABCE2 protein as the query (NP_193656). The phylogenetic analysis was performed using the NCBI accession numbers listed in **Supplementary Table S4** with MEGA X software (Kumar et al., 2018): the multiple sequence alignment and the phylogenetic tree were obtained using codon recognition with Muscle (Edgar, 2004b; Edgar 2004a), and the Neighbor-Joining method (Saitou and Nei, 1987), respectively.

### Accession numbers

Sequence data can be found at The Arabidopsis Information Resource (https://www.arabidopsis.org/) under the following accession numbers: *ABCE1* (At3g13640), *ABCE2* (At4g19210), *ACT2* (At3g18780), *ATHB8* (At4g32880), *OTC* (At1g75330), and *PIN1* (At1g735

## Results

### The *apiculata7-1* mutant exhibits a pleiotropic morphological phenotype

**Figure 1:**
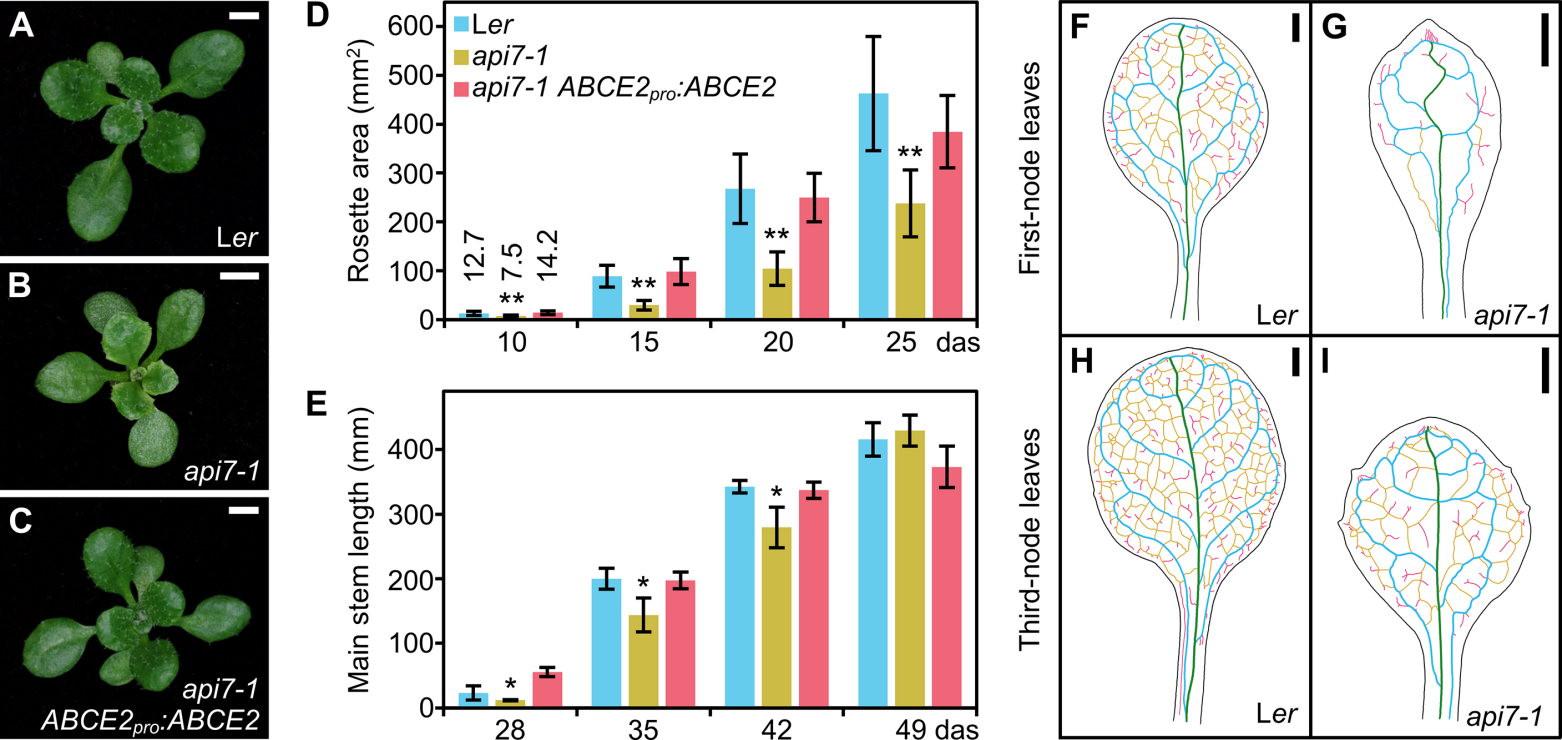
Morphological phenotype of the *api7-1* mutant. **(A–C)** Rosettes from **(A)** the wild-type L*er*, **(B)** the *api7-1* mutant, and **(C)** an *api7-1 ABCE2_pro_:ABCE2* mutant and transgenic plant. Pictures were taken 16 das. Scale bars indicate 2 mm. **(D, E)** Growth progression of **(D)** rosette area and **(E)** main stem length. **(D)** Bars indicate mean and **(E)** median values. Error bars represent **(D)** standard deviation and **(E)** median absolute deviation. Asterisks indicate a significant difference with L*er* in **(D)** a Student’s *t* test (10 < *n* < 17) or **(E)** a Mann-Whitney *U* test (*n* = 8) (*P < 0.05, **P < 0.001). **(F–I)** Venation pattern of *api7-1* first- and third-node leaves. Representative diagrams of mature **(F, G)** first- and **(H, I)** third-node leaves from **(F, H)** L*er* and **(G, I)** *api7-1* plants after visualization of 12 samples per organ and genotype. Margins were drawn in black, primary veins in green, secondary veins in blue, higher-order connected veins in yellow, and higher-order disconnected veins in pink. Organs were collected 21 das. Scale bars indicate 1 mm.

The *apiculata7-1* (*api7-1*) mutant, which we initially named *api7*, was isolated in a previous large-scale screen for EMS-induced mutations affecting leaf development (Berná et al., 1999). Its pleiotropic morphological phenotype includes a small rosette, a short primary root, and a delay in main stem growth (**Figures 1A–E**; **Supplementary Figure S1A**). The *api7-1* inflorescences and siliques are seemingly normal (**Supplementary Figure S1B–G**). The rosette leaves are pointed, indented, and pale, and contain a reduced amount of photosynthetic pigments, compared to its wild-type L*er* (**Figure 1**; **Supplementary Figure S1H**).

The pleiotropic phenotype of *api7-1* plants is reminiscent of mutants carrying loss-of-function alleles of genes encoding ribosomal proteins or ribosome biogenesis factors (Byrne, 2009; Horiguchi et al., 2011; Rosado et al., 2012; Weis et al., 2015; Micol-Ponce et al., 2018). As these mutations usually alter leaf vascular development, we cleared *api7-1* and L*er* leaves with chloral hydrate, and observed their venation patterns. We confirmed that *api7-1* fully expanded first-node and, to a lesser extent, third-node rosette leaves, contain fewer higher-order veins, and more prominent indentations and vascularized hydathodes, particularly in the leaf apex, than L*er* leaves (**Figures 1F–I**; **Supplementary Figure S2**). In contrast, these phenotypic traits seemed to be unaffected on *api7-1* cotyledons, cauline leaves, sepals, and petals (**Supplementary Figure S3**; **Supplementary Table S5**). The *ARABIDOPSIS THALIANA HOMEOBOX GENE 8* (*ATHB8*) gene is expressed in pre-procambial cells that will differentiate into veins (Baima et al., 1995). To determine the stage at which *api7-1* leaf venation pattern formation diverged from that of L*er*, we crossed *api7-1* plants to an *ATHB8_pro_:GUS* line, and studied the expression of the transgene in cleared first-node rosette leaf primordia of *api7-1 ATHB8_pro_:GUS* plants. Consistent with the slow growth phenotype of *api7-1* plants, we observed a delay in the emergence of first-node leaves (**Supplementary Figure S4**). In addition, *api7-1* primordia retained high GUS activity at their apical region even after the formation of the whole midvein (**Supplementary Figure S4M, N**), suggesting that an increased vascular differentiation in that region is responsible of the vascular phenotype of mature *api7-1* leaves (**Supplementary Figure S2**).

**Figure 2:**
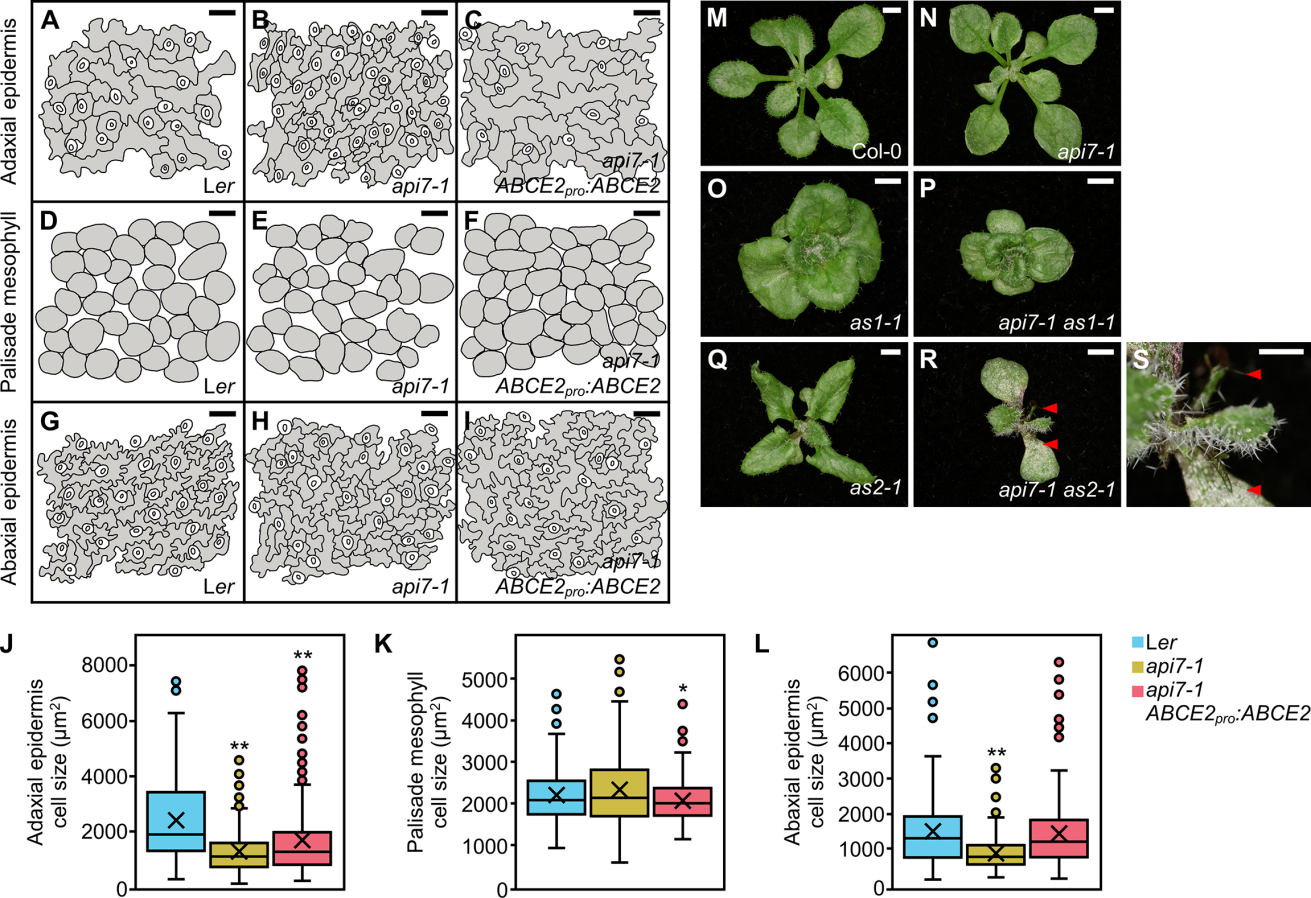
Dorsoventral patterning in *api7-1* leaves. **(A–L)** Leaf cell phenotypes of L*er*, *api7-1*, and *api7-1 ABCE2_pro_:ABCE2* plants. **(A–I)** Representative diagrams of the **(A–C)** adaxial epidermis, **(D–F)** subepidermal layer of palisade mesophyll, and **(G–I)** abaxial epidermis, from **(A, D, J, G)** L*er*, **(B, E, H)** *api7-1*, and **(C, F, I)** *api7-1 ABCE2_pro_:ABCE2* plants. **(J–L)** Boxplot distributions of cell area in the **(J)** adaxial epidermis, **(K)** subepidermal layer of palisade mesophyll, and **(L)** abaxial epidermis, from first-node leaves collected 21 das. Other details as described in the legend of **Supplementary Figure S1** for its **(A)** section. Between 144 and 351 cells were analyzed from at least 4 different samples. Asterisks indicate a significant difference with the L*er* wild-type in a Student’s *t* test (*P < 0.05, **P < 0.001). **(M–S)** Genetic interactions of *api7-1* with *as1-1* and *as2-1*. Rosettes from the wild-type **(M)** L*er*, the **(N)** *api7-1*, **(O)** *as1-1*, and **(Q)** *as2-1* single mutants, and the **(P)** *api7-1 as1-1* and **(R)** *api7-1 as2-1* double mutants. **(G)** Close up of **(F)**. Red arrowheads indicate radial leaves. Pictures were taken 16 das. Scale bars indicate **(M–R)** 2 and **(S)** 1 mm.

Cleared *api7-1* first-node leaves also showed a marked reduction in cell size in the abaxial and adaxial epidermal layers, but not in the palisade mesophyll (**Figure 2**). Indeed, the adaxial epidermis of *api7-1* was more similar to its abaxial epidermis than to the adaxial epidermis of the wild-type. This observation suggests that *api7-1* leaves are abaxialized, as has been reported for other mutants affected in the translation machinery (Pinon et al., 2008; Yao et al., 2008; Horiguchi et al., 2011; Moschopoulos et al., 2012; Casanova-Sáez et al., 2014; Mateo-Bonmatí et al., 2015; Matsumura et al., 2016). *ASYMMETRIC LEAVES 1* (*AS1*) and *AS2* encode transcription factors involved in leaf dorsoventral patterning. Double mutant combinations of *as1* or *as2* with mutations in genes encoding ribosomal proteins or other components of the translation machinery usually produce synergistic phenotypes. These phenotypes are easily distinguished by the presence of trumpet-shaped (peltate) or radial leaves originated by partial or complete loss of dorsoventrality, respectively (Pinon et al., 2008; Yao et al., 2008; Horiguchi et al., 2011; Moschopoulos et al., 2012; Casanova-Sáez et al., 2014; Mateo-Bonmatí et al., 2015; Matsumura et al., 2016). We obtained *api7-1 as1-1* and *api7-1 as2-1* double mutants in the Col-0 background; these double mutants exhibited additive and synergistic phenotypes, respectively (**Figures 2M–S**). The presence of radial leaves in *api7-1 as2-1* plants further supports a role for API7 in translation (**Figures 2R, S**).

### *api7-1* is a viable mutant allele of the *ABCE2* gene

The *api7-1* mutation was previously mapped to chromosome 4 (Robles and Micol, 2001). To identify the mutated gene, we combined map-based cloning and next-generation sequencing, as previously described (Mateo-Bonmatí et al., 2014). First, we performed linkage analysis of an F_2_ mapping population, which allowed us to delimit a candidate interval encompassing 30 annotated genes (**Supplementary Figure S5A**). We then sequenced the whole *api7-1* genome and identified 4 EMS-type nucleotide substitutions within the candidate interval (**Supplementary Table S6**). Only one of these, a C→T transition in At4g19210, was predicted to be a missense mutation causing a Pro138→Ser substitution (**Supplementary Figure S5B**). The At4g19210 gene encodes ABCE2, a protein of 605 amino acids (68.39 kDa). The Pro138 residue, at the beginning of the HLH motif located within NBD1, is conserved across all eukaryotic ABCE proteins tested, except in *Caenorhabditis elegans* (**Supplementary Figure S6**), in which it seems to have evolved more divergently (Chen et al., 2006). The conservation of this residue suggests that it is necessary for the proper function of ABCE proteins, probably for the interactions with the ribosome, which mainly occur through the HLH and hinge motifs (Heuer et al., 2017; Nürenberg-Goloub et al., 2020; Kratzat et al., 2021).

To confirm that the mutation found in At4g19210 causes the phenotype of the *api7-1* mutant, we obtained the *ABCE2_pro_:ABCE2* transgene, which was transferred into *api7-1* plants. This transgene completely restored the wild-type rosette leaf shape and stem height (**Figures 1C–E**), as well as the photosynthetic pigment content (**Supplementary Figure S1H**). The *ABCE2_pro_:ABCE2* transgene partially restored leaf epidermal cell sizes and root length (**Figure 2**; **Supplementary Figure S1A**). To provide further confirmation that *api7-1* is an allele of *ABCE2*, we performed an allelism test using GABI_509C06 plants (Kleinboelting et al., 2012), which were heterozygous for a T-DNA insertion in the 10^th^ exon of At4g19210 (**Supplementary Figure S5B**). We named *api7-2* the insertional allele in GABI_509C06. In the F_2_ population of this cross, no *api7-2/api7-2* plants were found, and *api7-1/api7-2* and *api7-1/api7-1* plants were phenotypically similar, confirming that these mutations are allelic and that loss of function of *ABCE2* is responsible for the phenotype of the *api7-1* mutant (**Supplementary Figure S7A–C**).

The absence of *api7-2/api7-2* plants derived from GABI_509C06 seeds, and of ungerminated seeds in the F_1_ progeny of selfed heterozygous *ABCE2/api7-2* plants, suggested an early lethality of this mutant allele. We dissected immature siliques from *ABCE2/api7-2* plants and found 21.95% aborted seeds (*n* = 328), which fits a 1:3 Mendelian segregation ratio (χ^2^ = 1.63; *P* = 0.202; degrees of freedom = 1). Col-0 siliques showed 1.37% aborted ovules (*n* = 148; **Supplementary Figure S7D, E**). The lethality caused by *api7-2* suggests that it is a null allele of *ABCE2*, while *api7-1* is hypomorphic.

### The Arabidopsis genome contains two partially redundant *ABCE* paralogs

To gather information about the origin of the two Arabidopsis *ABCE* paralogs, we performed a phylogenetic analysis of *ABCE* coding sequences from some Rosidae species (rosids; **Supplementary Figure S8**). Among them, we found that other Brassicaceae genomes also encode ABCE1 and ABCE2 proteins, but only *ABCE2* was identified in *Cardamine hirsuta*. Consistent with the whole-genome triplication in *Brassica rapa* (Zhang et al., 2018), we found two and three *Brassica rapa ABCE1* and *ABCE2* sequences, respectively. All Brassicaceae *ABCE1* genes grouped together in the phylogenetic tree, and separately from their *ABCE2* paralogs, which formed other subclade. Although both *ABCE1* and *ABCE2* paralogs have been conserved, *ABCE1* orthologs have evolved more rapidly than their *ABCE2* paralogs, whose short evolutionary distances indicate that they are under strong evolutionary pressure, as expected for an essential gene.

As previously described (Braz et al., 2004; Sarmiento et al., 2006), we observed that *ABCE2* is highly expressed throughout all Arabidopsis developmental stages. By contrast, the expression levels of its *ABCE1* paralog are very low in all studied organs, in which first-node leaves and flowers show the lowest and highest expression levels, respectively (**Supplementary Figure S9A, B**). The expression level of *ABCE1* in *api7-1* rosettes was the same as in L*er*, showing that ABCE1 cannot compensate for the partial loss of ABCE2 function in rosettes (**Supplementary Figure S9C**). However, *api7-1* flowers, where we observed the highest *ABCE1* expression levels, do not show apparent aberrations (**Supplementary Figure S1C**), suggesting that ABCE1 and ABCE2 might play similar roles during flower development.

**Figure 3:**
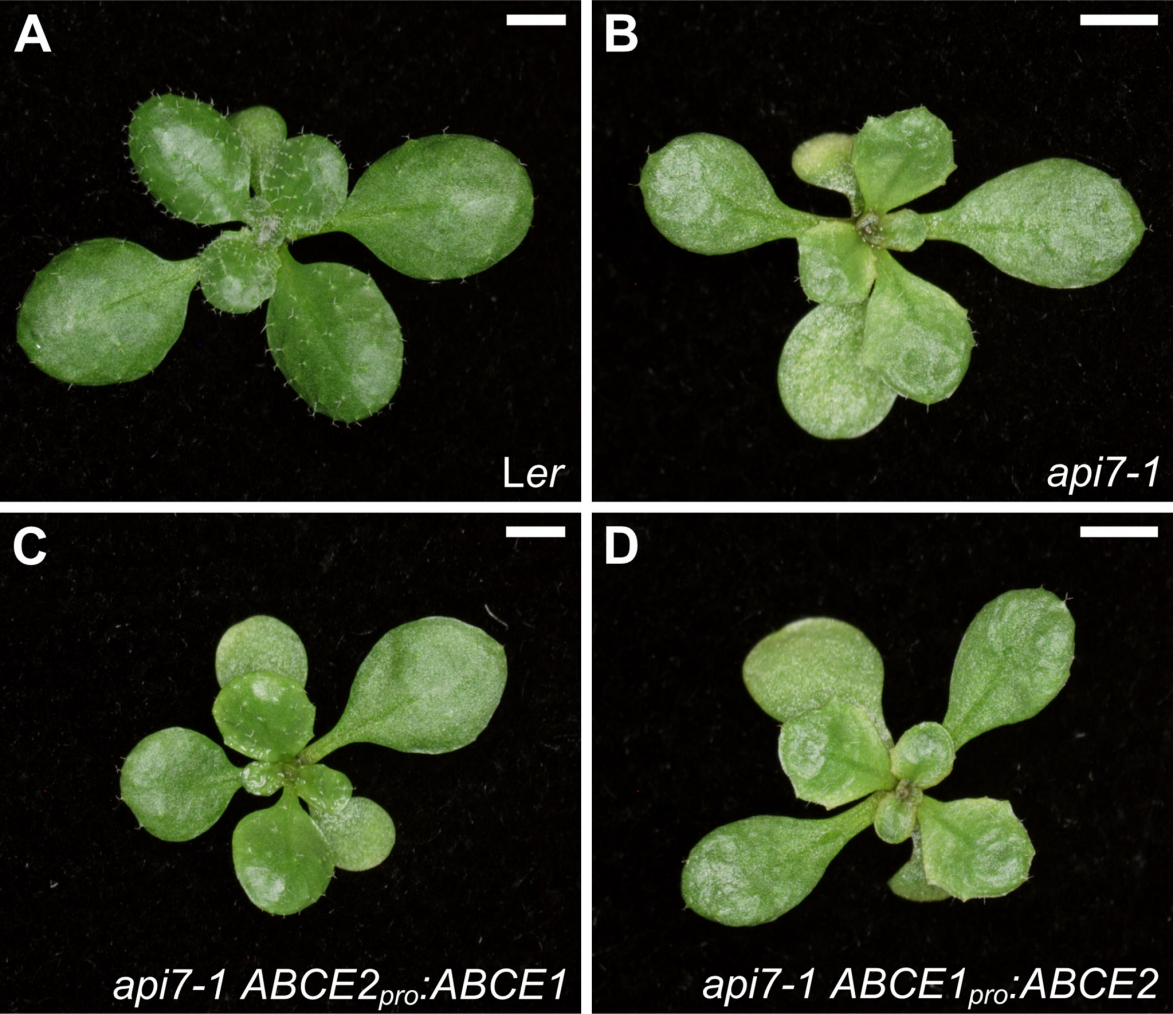
Effects of the *ABCE2_pro_:ABCE1* and *ABCE1_pro_:ABCE2* transgenes on the morphological phenotype of the *api7-1* mutant. Rosettes from **(A)** L*er*, **(B)** *api7-1*, **(C)** *api7-1 ABCE2_pro_:ABCE1*, and **(D)** *api7-1 ABCE1_pro_:ABCE2* plants. Pictures were taken 14 das. Scale bars indicate 2 mm.

The ABCE1 and ABCE2 proteins share 80.8% identity, suggesting that ABCE1 and ABCE2 might be functionally equivalent. To test this hypothesis, we performed a promoter swapping assay between the *ABCE1* and *ABCE2* genes (**Figure 3**). As expected from the lower expression levels driven by the *ABCE1* promoter, *api7-1 ABCE1_pro_:ABCE2* plants were indistinguishable from *api7-1* mutants, highlighting that correct protein levels are as important as the correct sequence for normal *ABCE2* function. In contrast, the *ABCE2_pro_:ABCE1* transgene partially rescued the *api7-1* phenotype, showing that the ABCE1 and ABCE2 proteins are functionally redundant. Further supporting equivalent functions for ABCE1 and ABCE2, the constitutive expression of *ABCE1* with a *35S_pro_:ABCE1* transgene fully restored a wild-type phenotype in *api7-1* rosettes (**Supplementary Figure S10**).

### ABCE2 is a cytoplasmic protein that physically associates with components of the translation machinery

**Figure 4:**
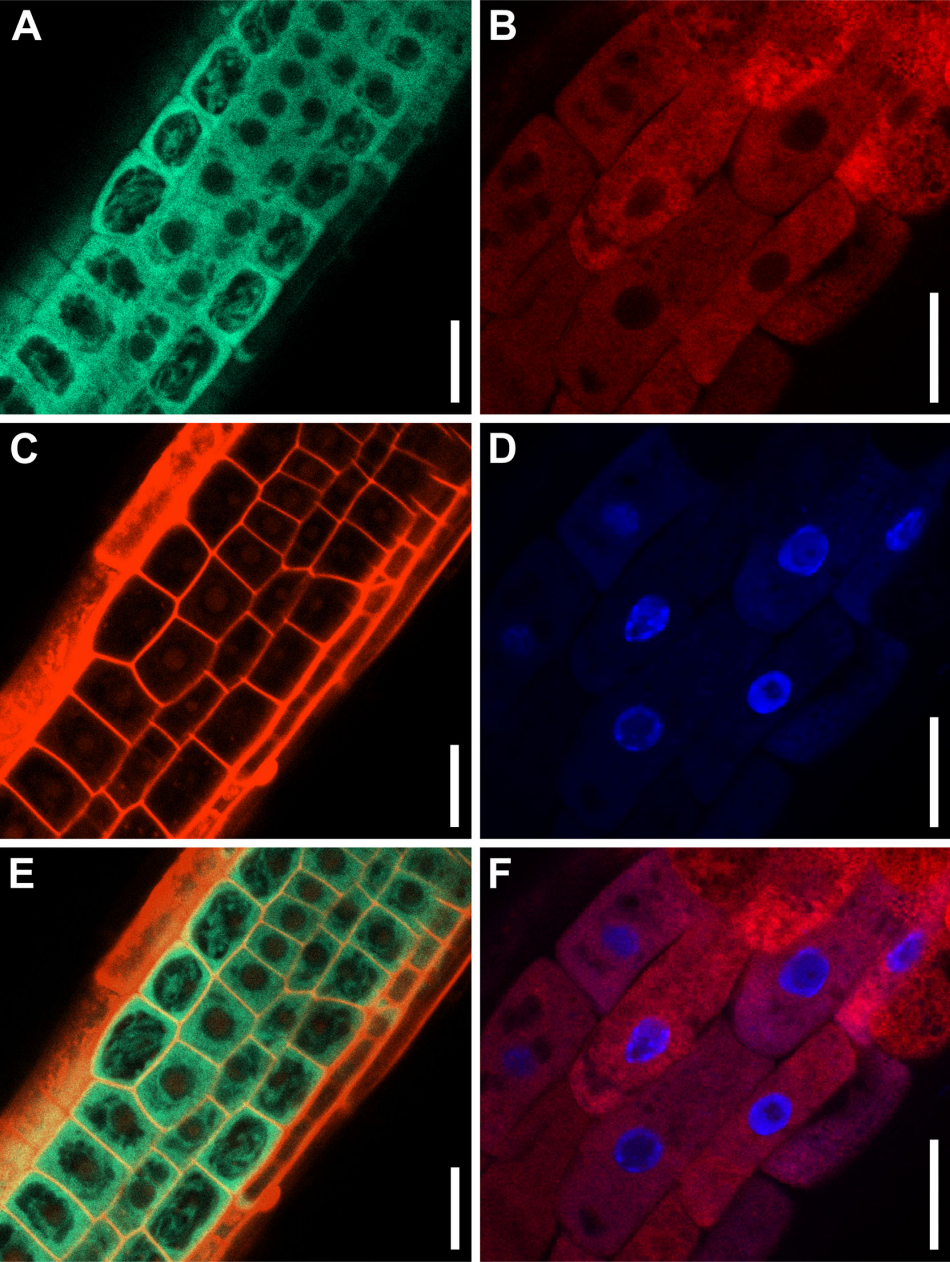
Subcellular localization of the ABCE2 protein in cells from the root elongation zone. Confocal laser scanning micrographs of **(A, C, E)** L*er 35S_pro_:ABCE2:GFP* and **(B, D, F)** *api7-1 35S_pro_:ABCE2:YFP* transgenic plants. Fluorescent signals correspond to **(A)** GFP, **(B)** YFP, **(C)** propidium iodide, and **(D)** DAPI staining, and the overlay of **(E)** GFP and propidium iodide, and **(F)** YFP and DAPI. Pictures were taken **(A, C, E)** 14 and **(B, D, F)** 5 das. Scale bars indicate 20 μm.

To determine the subcellular localization of Arabidopsis ABCE2, we obtained in-frame translational fusions of ABCE2 to GFP and YFP, driven by the *35S* promoter: *35S_pro_:ABCE2:GFP* and *35S_pro_:ABCE2:YFP*. We visualized the ABCE2:GFP fusion protein in the cytoplasm of root cells treated with propidium iodide, which mainly stains cell walls, and the ABCE2:YFP fusion protein in roots stained with the nucleoplasm dye DAPI, and confirmed the nuclear exclusion of ABCE2 (**Figure 4**).

**Figure 5:**
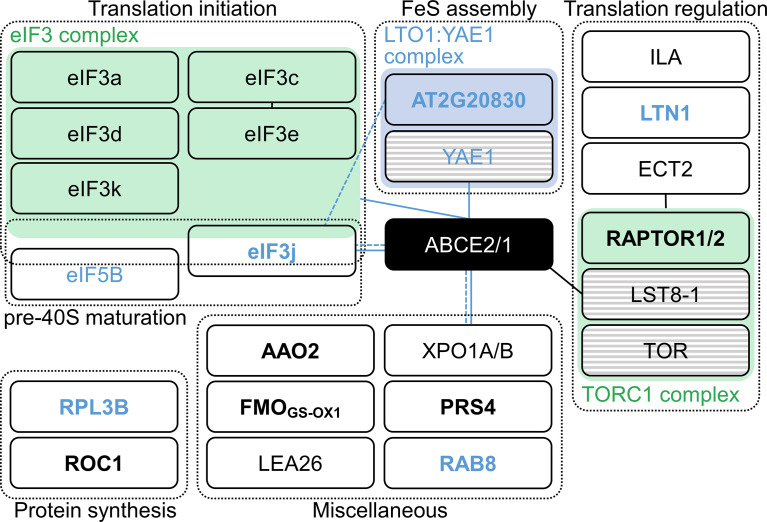
Proteins identified in an ABCE2:YFP co-immunoprecipitation assay. Proteins were grouped within dashed boxes according to their annotated functions for Arabidopsis (names in black letters) or orthologous (names in blue letters) proteins. Green and blue boxes represent complexes that have been described in Arabidopsis and other species, respectively. Proteins in striped boxes were not identified in our assay but have been included in this Figure because they are known to belong to a given complex. Continuous and dashed lines connecting boxes indicate physical and genetic interactions described elsewhere for Arabidopsis (black) or other species (blue), respectively. For references, see **Supplementary Table S8**. Names in bold and plain letters indicate proteins unique to or enriched in ABCE2:YFP samples, respectively.

To investigate the function of ABCE2, we performed a co-immunoprecipitation assay using the ABCE2:YFP protein from a homozygous T_3_ *api7-1 35S_pro_:ABCE2:YFP* line, which was phenotypically wild-type, confirming that the fusion protein is functional (**Supplementary Figure S11A–C**). We checked the purification of the fusion protein by western blotting using an anti-GFP antibody (**Supplementary Figure S11D–F**). Using LC-ESI-MS/MS, we identified 20 putative interactors of ABCE2, of which 13 participate in translation (6 subunits of the eIF3 complex, eIF5B, RPL3B, and ROTAMASE CYP 1 [ROC1]) or in its regulation (At5g58410, EVOLUTIONARILY CONSERVED C-TERMINAL REGION 2 [ECT2], ILITYHIA [ILA], and REGULATORY-ASSOCIATED PROTEIN OF TOR 1 [RAPTOR1] or RAPTOR2), and two others had previously been shown to interact with ABCE2 orthologs (At2g20830, and EXPORTIN 1A [XPO1A] or XPO1B). The functions of the remaining 5 proteins that co-immunoprecipitated with ABCE2 are unclear and these proteins were therefore set aside for future characterization (**Figure 5**; **Supplementary Figure S12**; **Supplementary Tables S7**, **S8**; **Supplementary Data Set 1**).

At2g20830 encodes a folic acid binding/transferase that shares 30.2% and 26.7% identity with human and *Saccharomyces cerevisiae* Lto1 (named after “required for biogenesis of the large ribosomal subunit and initiation of translation in oxygen”), respectively (human and *S. cerevisiae* Lto1 proteins share 27.8% identity). Lto1, together with Yae1, constitute an essential complex for FeS cluster assembly on ABCE1 (Zhai et al., 2014; Paul et al., 2015; Zhu et al., 2020; Prusty et al., 2021). Despite the observation that At2g20830 protein was predicted to localize to mitochondria, the conservation level of this protein with its yeast and human Lto1 orthologs prompted us to consider At2g20830 an ABCE2 interactor. Indeed, At2g20830 may be necessary for FeS cluster assembly on ABCE2.

Our co-immunoprecipitation assay suggested that Arabidopsis ABCE2 interacts with 6 of the 13 eIF3 subunits: eIF3a, c, d, e, k, and j. In *S. cerevisiae*, those interactions have been related to the presence of the ABCE1 protein in the 40S subunit after ribosome dissociation, until late steps of initiation of a new cycle of translation (Heuer et al., 2017; Mancera-Martínez et al., 2017; Kratzat et al., 2021). Interestingly, the interaction between the non-stoichiometric subunit eIF3j and ABCE1 also occurs in humans and *S. cerevisiae*. In these species, eIF3j acts as an accessory factor for ABCE1-mediated ribosome dissociation (Young and Guydosh, 2019; Kratzat et al., 2021), a function that seems to be conserved in Arabidopsis.

To corroborate and extend the list of interactions between ABCE2 and components of the translation machinery, we performed a tandem affinity purification (TAP) assay of a GSRhino-TAP-tagged ABCE2 bait, obtained from cell suspension cultures, and identified its putative interactors by nano LC-MS/MS (**Supplementary Data Set 2**). We found that 81 proteins co-purified with ABCE2, of which 28 were ribosomal proteins (**Supplementary Data Set 2E**).

### The *api7-1* mutation perturbs auxin metabolism

To gain insight into the biological processes affected in the *api7-1* mutant, we performed an RNA-seq analysis of L*er* and *api7-1* shoots collected 14 das. We identified 3218 downregulated and 2135 upregulated genes in the *api7-1* mutant (**Supplementary Data Set 3A**). A gene ontology (GO) enrichment analysis performed separately for down- and upregulated genes showed that the downregulated genes were mainly related to responses to abiotic and biotic stresses and protein post-translational modifications. In contrast, upregulated genes grouped into more diverse Biological Process terms (**Supplementary Data Set 3B, C**). Among them, we found three terms related to auxin (response to auxin [GO:0009733], auxin-activated signaling pathway [GO:0009734], and auxin polar transport [GO:0009926]).

**Figure 6:**
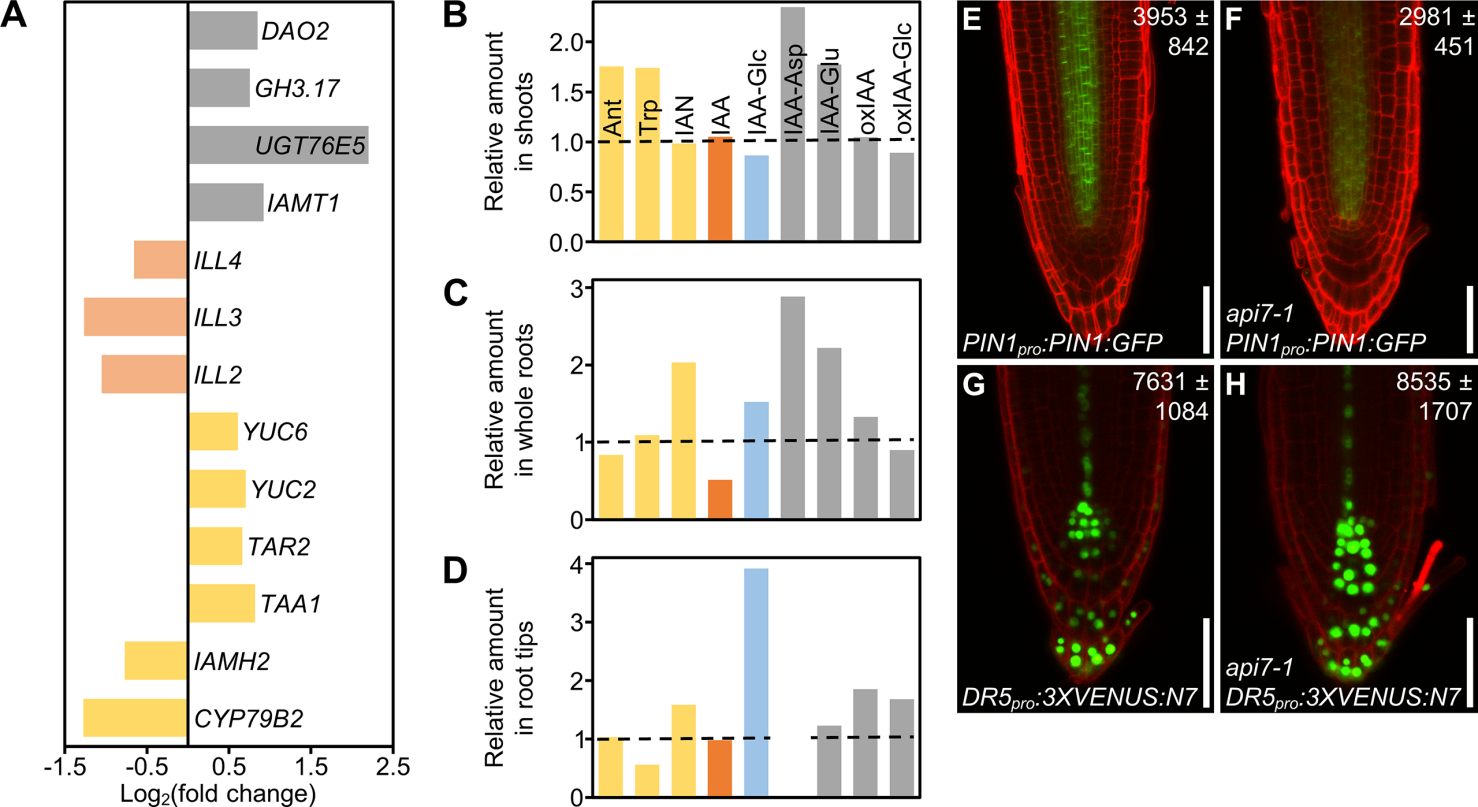
Auxin metabolism, transport and signaling are altered in *api7-1* plants. **(A)** Expression levels of genes related to auxin metabolism (biosynthesis, yellow; activation, pale orange; storage and catabolism, grey) in *api7-1* shoots 14 das. Values are shown as the binary logarithm of the fold change between *api7-1* and L*er* mean reads. Mean reads were calculated from three biological replicates. **(B–D)** Relative amounts of some IAA precursors (yellow; Ant, anthranilate; Trp, tryptophan; IAN, indole-3-acetonitrile), IAA (orange), the IAA storage molecule IAA-Glc (glycosylated IAA; blue), and IAA catabolites (grey; IAA-Asp and IAA-Glu, IAA conjugated to aspartate and glutamate, respectively; oxIAA, 2-oxindole-3-acetic acid; oxIAA-Glc, glycosylated 2-oxindole-3-acetic acid) in *api7-1* **(B)** shoots, **(C)** whole roots, and **(D)** root tips 9 das. IAA-Asp was not detected in root tips. The mean amounts of each metabolite in L*er* were used as the reference value (dashed lines; see **Supplementary Figure S14**). Mean amounts were calculated from four biological replicates. **(E–H)** Visualization of the expression of reporter transgenes for auxin **(E, F)** transport and **(G, H)** perception, in **(E, G)** wild-type and **(F, H)** *api7-1* roots. Cell walls were stained with propidium iodide. Values indicate average fluorescence intensities ± standard deviation from **(E, F)** GFP and **(G, H)** VENUS, which are significantly different from the wild type in a Student’s *t* test [*P <* 0.001, **(E, F)** *n* = 25; *P <* 0.05, **(G, H)** *n* = 27]. Pictures were taken 5 das. Scale bars indicate 50 µm.

We observed that four out of the six genes that participate in the main auxin biosynthesis pathway in shoots were upregulated. They included two of the three genes encoding enzymes that convert tryptophan (Trp) into indole-3-pyruvic acid (IPyA), *TRYPTOPHAN AMINOTRANSFERASE OF ARABIDOPSIS 1* (*TAA1*), and *TAA1-RELATED 2* (*TAR2*), and two *YUCCA* genes (*YUC2* and *YUC6*) encoding enzymes that turn IPyA into indole-3-acetic acid (IAA) (Cheng et al., 2007; Casanova-Sáez et al., 2021; Kneuper et al., 2021). However, the expression of two genes involved in a secondary pathway for IAA biosynthesis, *CYTOCHROME P450, FAMILY 79, SUBFAMILY B, POLYPEPTIDE 2* (*CYP79B2*) and *IAMHYDROLASE12* (*IAMH2*) were downregulated (Gao et al., 2020). We also found that four genes involved in auxin inactivation, *IAA CARBOXYLMETHYLTRANSFERASE 1* (*IAMT1*), *GRETCHEN HAGEN 3.17* (*GH3.17*), *DIOXYGENASE FOR AUXIN OXIDATION 2* (*DAO2*), and *UDP-GLYCOSYLTRANSFERASE 76E5* (*UGT76E5*) were upregulated, and that three genes involved in auxin reactivation, *IAA-LEUCINE RESISTANT (ILR)-LIKE 2* (*ILL2*), *ILL3*, and *ILL4* (Takubo et al., 2020; Casanova-Sáez et al., 2021; Hayashi et al., 2021; Mateo-Bonmatí et al., 2021), were downregulated, probably in response to high auxin levels (**Figure 6A**). In this manner, our transcriptional data point to an increase in auxin biosynthesis in *api7-1* shoots, which might be partially or fully compensated by reducing the synthesis rate in secondary pathways, and by inactivating and preventing the reactivation of IAA.

To directly assess our hypothesis, we checked the content of indol-3-acetic acid (IAA), the main auxin in most plants, as well as some of its precursors and inactive forms in *api7-1* and L*er* shoots, whole roots and root tips. We found a similar trend within the three tissues: an increase in IAA catabolism, as suggested by the RNA-seq results, and an accumulation of its precursors, when compared to L*er* tissues (**Figures 6B–D**; **Supplementary Figure S13**). The inactivation of IAA in *api7-1* shoots and whole roots mainly occurs through glutamate (IAA-Glu) and aspartate (IAA-Asp) conjugation, while in root tips occurs through IAA oxidation (oxIAA), and subsequent glycosylation (oxIAA-glc). *api7-1* shoots accumulate Ant, a substrate for Trp biosynthesis, and Trp itself, the main precursor for IAA biosynthesis. Whole roots and root tips accumulate IAN, another IAA precursor, and store the inactive glycosylated IAA (IAA-glc). The IAA levels were normal in shoots and root tips, suggesting that auxin homeostasis is maintained in *api7-1*. However, the IAA levels in whole roots were decreased by almost 50%, maybe due to its high inactivation levels. Trp levels were low in root tips, suggesting that it might be converted to IAN, which is overaccumulated, or to IAA, which seems to be stored and catabolized to maintain its normal levels.

In agreement with the reduced levels of IAA in *api7-1* whole roots, the levels of a fusion protein between the auxin exporter PIN-FORMED1 (PIN1) and GFP (PIN1:GFP) in *api7-1 PIN1_pro_:PIN1:GFP* roots were lower than in L*er* roots (**Figures 6E, F**; **Supplementary Figure S14A**). In addition, we observed that the expression of the synthetic auxin-responsive promoter *DR5* in *api7-1 DR5_pro_:3XVENUS:N7* root tips, measured as the fluorescence intensity of 3XVENUS:N7, was slightly increased in comparison to L*er DR5_pro_:3XVENUS:N7* root tips, indicating that auxin signaling might be also altered in *api7-1* (**Figures 6G,H**; **Supplementary Figure S14B**).

### Genes related to iron homeostasis are deregulated in *api7-1* plants

Interestingly, we also found in our RNA-seq assay that iron ion homeostasis and transport (GO:0055072 and GO:0006826), and response to iron and sulfur ion starvation (GO:0010106 and GO:0010438) terms were among the most enriched in the analysis of upregulated genes (**Supplementary Data Set 3B**). For instance, genes related to iron uptake, such as *IRON-REGULATED TRANSPORTER 1* (*IRT1*) and *FERRIC CHELATE REDUCTASE DEFECTIVE 1* (*FRD1*) (Eide et al., 1996; Robinson et al., 1999), or to iron mobility, such as *NATURAL RESISTANCE-ASSOCIATED MACROPHAGE PROTEIN 3* (*NRAMP3*) and *NRAMP6* (Lanquar et al., 2005; Li et al., 2019), and several genes encoding transcription factors induced by iron and sulfur deficiencies were upregulated in the *api7-1* mutant (**Supplementary Figure S15**). These pathways might be activated in *api7-1* plants to provide iron and sulfur for FeS cluster biogenesis, probably to compensate for the depletion in ABCE2 protein. Indeed, the gene that encodes the Arabidopsis NEET protein (termed after its conserved Asn-Glu-Glu-Thr sequence near its C-terminus) (Colca et al., 2004), which participates in FeS cluster transference during its biogenesis (Nechushtai et al., 2012; Zandalinas et al., 2020), was also upregulated (**Supplementary Figure S15**).

Consequently, the iron content in *api7-1* cells might be higher than in the wild type, and might be inducing the formation of reactive oxygen species (ROS), as occurs in mutants affected in free iron storage (Briat et al., 2010). In agreement with this assumption, several terms related to oxidative stress responses were also enriched. Specifically, we found that *FERRITIN 2* (*FER2*) and *FER3*, which encode iron storage proteins in response to high iron levels to avoid oxidative damage (Briat et al., 2010; Reyt et al., 2015), were upregulated (**Supplementary Figure S15**). In addition, previous studies have shown that ROS prevent FeS cluster assembly into ABCE proteins, which is necessary for their activity in ribosome recycling (Alhebshi et al., 2012; Sudmant et al., 2018; Zhu et al., 2020). In this manner, *api7-1* plants might experience a positive feedback loop where a response to iron starvation due to reduced activity of ABCE2 increases iron levels, inducing the production of ROS which, in turn, further disturbs ABCE2 activity. Nevertheless, further studies are needed to ascertain a potential relation among ABCE2 activity, iron homeostasis, and oxidative stress, which were beyond the scope of this work.

## Discussion

### Plant ABCE proteins participate in translation in a cross-kingdom conserved manner

In this work, we studied Arabidopsis ABCE2, one of the most conserved proteins among archaea and eukaryotes (Hopfner, 2012). Archaea, fungi, and animal ABCE proteins dissociate cytoplasmic ribosomes into their 30S/40S and 50S/60S subunits at different translation-related events (Nürenberg-Goloub and Tampé, 2019). After ribosome dissociation, an ABCE escorts the 30S/40S subunit until the late steps of translation initiation, preventing premature joining of the 50S/60S subunit into the preinitiation complex (Heuer et al., 2017; Nürenberg-Goloub et al., 2020).

The crosslinking performed on the tissue used for the ABCE2 co-immunoprecipitation assay did not allow us to discern direct from indirect ABCE2 interactors. However, the interactions with XPO1A/B, eIF3j, and the protein encoded by At2g20830 are very likely to be direct, in agreement with previous studies in non-plant species (Kirli et al., 2015; Paul et al., 2015; Young and Guydosh, 2019; Kratzat et al., 2021). In contrast, the interactions observed with other eIF3 subunits, RPL3B, ROC1, ECT2, ILA, RAPTOR1/2, and the protein encoded by At5g58410, which is annotated as E3 ubiquitin-protein ligase listerin (LTN1; UniProt code: Q9FGI1) might occur indirectly as they are part of or interact with the translation machinery (Coaker et al., 2006; Shao et al., 2013; Kashima et al., 2014; Sesma et al., 2017; Wang et al., 2017; Arribas-Hernández et al., 2018; Faus et al., 2018; Izquierdo et al., 2018; Scutenaire et al., 2018; Wei et al., 2018). However, the interaction between ABCE2 and eIF5B, which does not seem to occur in *S. cerevisiae* and mammals (Heuer et al., 2017; Mancera-Martínez et al., 2017), will require further exploration.

Further supporting a role for ABCE2 in translation, we observed a synergistic interaction in the *api7-1 as2-1* double mutant, which shows radial leaves, as previously described for double mutant combinations of loss-of-function alleles of *AS1* or *AS2* and other components of the translation machinery (Pinon et al., 2008; Yao et al., 2008; Horiguchi et al., 2011; Moschopoulos et al., 2012; Casanova-Sáez et al., 2014; Mateo-Bonmatí et al., 2015). In this manner, our results suggest that Arabidopsis and, by extension, all plant ABCEs, probably dissociate cytoplasmic ribosomes, as has been reported for species of other kingdoms (Nürenberg-Goloub and Tampé, 2019). In addition, previous works also support a conserved role for the Arabidopsis ABCE2 and human ABCE1 proteins as suppressors of RNA silencing (Braz et al., 2004; Sarmiento et al., 2006; Kärblane et al., 2015; Mõttus et al., 2020). However, we did not find any ABCE2 interactor potentially involved in this process, nor any enriched ontology term related to gene silencing in our RNA-seq assay. This might be due to the need for a cellular environment that triggers RNA silencing and exposes this novel function of ABCE proteins. Further research will help to assess a potential relationship between ribosome recycling and RNA silencing.

### The developmental defects of the *api7-1* mutant have different causes

The essential function of ABCEs has been confirmed in several species: null alleles of *ABCE* genes in all studied organisms are lethal, while hypomorphic alleles cause severe growth aberrations (Navarro-Quiles et al., 2018). In this work, we describe the first hypomorphic and null alleles of the Arabidopsis *ABCE2* gene, *api7-1* and *api7-2*, respectively. We showed that the *api7-2* mutation is lethal and that *api7-1* plants share developmental defects with other mutants affected in genes encoding ribosomal proteins or ribosome biogenesis factors. These phenotypic traits include an aberrant leaf venation pattern (Horiguchi et al., 2011), as is the case for the *api7-1* mutant. Indeed, a mutant allele of *SIL3*, the *Cardamine hirsuta ABCE* ortholog, also causes venation pattern defects which may be related to an aberrant auxin transport and signaling (Kougioumoutzi et al., 2013).

In agreement with the involvement of local auxin biosynthesis, polar transport and signalling in vascular development (Verna et al., 2019; Kneuper et al., 2021), we observed that auxin metabolism and auxin-induced genes were upregulated in the *api7-1* mutant. In addition, a previous study found that the IAA content in *api7-1* seedlings was slightly reduced when compared to L*er* (Pěnčík et al., 2018). In our experimental conditions, IAA levels in *api7-1* shoots and root tips were normal, but reduced in whole roots. However, the general accumulation of IAA precursors and catabolites in *api7-1* seedlings suggests that, despite auxin metabolism in *api7-1* is perturbed, auxin homeostasis is maintained through different compensation mechanisms, like occurs in other mutants affected in IAA metabolism (Mellor et al., 2016; Porco et al., 2016; Zhang et al., 2016). In this sense, the altered levels of IAA precursors and catabolites, and the deregulation of auxin signalling might contribute to the aberrant phenotype of *api7-1* plants. Our transcriptomic results also point to the deregulation of additional biological pathways as potential contributors to the *api7-1* phenotype: one of them might be an increased production of ROS, caused by a potential deregulation of iron and sulfur homeostasis.

### The Arabidopsis ABCE1 and ABCE2 proteins are functionally redundant

Arabidopsis has two ABCE paralogs, *ABCE1* and *ABCE2* (Sánchez-Fernández et al., 2001; Verrier et al., 2008). In agreement with previous literature (Braz et al., 2004; Sarmiento et al., 2006), we observed that *ABCE1* expression levels are low in all studied organs and throughout development, in contrast to the high expression of *ABCE2*. We also showed that *ABCE1* is unable to complement *ABCE2* dysfunction in *api7-1* rosettes *per se*.

However, the wild-type phenotype of *api7-1* flowers, where we found the highest expression levels of *ABCE1*, and the ability of *ABCE2_pro_:ABCE1* and *35S_pro_:ABCE1* to complement the *api7-1* mutant phenotype, indicate that the ABCE1 protein is functional and that it may contribute to translation in the reproductive tissues of wild-type plants. In addition, our phylogenetic analysis showed that the *ABCE* duplication event occurred early during the evolution of Brassicaceae, and that at least five species from this clade conserved an *ABCE1* gene that evolved more rapidly than its *ABCE2* paralog, suggesting that *ABCE2* conserved the ancestral function, whereas *ABCE1* underwent hypofunctionalization (Veitia, 2017).

ABCE proteins are encoded by a single gene in most species, and they are essential for archaea and eukaryotes (Navarro-Quiles et al., 2018). Due to their importance, the molecular mechanisms by which they participate in ribosome recycling have been deeply studied, and remain a subject of intense research (Heuer et al., 2017; Mancera-Martínez et al., 2017; Nürenberg-Goloub et al., 2018; Nürenberg-Goloub et al., 2020; Kratzat et al., 2021). Nevertheless, the biological consequences of ABCE depletion or disruption are poorly understood in all organisms. In this sense, future research linking the molecular function of ABCEs with the phenotypic output of their dysfunction will contribute to determining the pathways through which translation modulates development, as we show here with the isolation and study of the hypomorphic and viable *api7-1* allele of the Arabidopsis *ABCE2* gene.

## Data availability statement

The raw data from genome resequencing and RNA-seq were deposited in the Sequence Read Archive (https://www.ncbi.nlm.nih.gov/sra/) database under accession numbers SRP065876 and PRJNA719000, respectively. The mass spectrometry proteomics data from the co-immunoprecipitation and TAP assays have been deposited to the ProteomeXchange Consortium via the PRIDE (Perez-Riverol et al., 2022) partner repository with the dataset identifiers PXD036412 and PXD036626, respectively.

## Author contributions

JLM conceived, designed, and supervised the research, provided resources, and obtained funding. Several experiments were codesigned by CN-Q, EM-B, and JLM, CN-Q performed most of the experiments. EM-B obtained the *ABCE2_pro_:ABCE2* and *35S_pro_:ABCE2:GFP* transgenes, and contributed to the phenotypic analysis of *api7-1*. EM-B and HC obtained the *api7-1 as* double mutants. CN-Q and HC performed the phylogenetic analysis. HC and AM-L screened the Micol collection of leaf mutants for abnormal leaf venation patterns. PR performed preliminary morphometric analysis of cells and venation from *api7-1* leaves. JŠ and KL performed the IAA metabolite profiling. YF and VR performed the TAP assay. MRP, HC, and EM-B performed the mapping and cloning of the *api7-1* mutation. CN-Q and JLM wrote the manuscript. All authors revised and approved the manuscript.

## Funding

This work was supported by the Ministerio de Ciencia e Innovación of Spain [PID2019-105495GB-I00 (MCI/AEI/FEDER, UE), to VR; PGC2018-093445-B-I00 (MCI/AEI/FEDER, UE), to JLM]; and the Generalitat Valenciana [PROMETEO/2019/117, to JLM and MRP]. CN-Q and EM-B held predoctoral fellowships from the Universidad Miguel Hernández [401PREDO] and the Ministerio de Educación, Cultura y Deporte of Spain [FPU13/00371], respectively. KL and JŠ were funded by the Knut and Alice Wallenberg Foundation (KAW 2016.0341 and KAW 2016.0352) and the Swedish Governmental Agency for Innovation Systems (VINNOVA 2016-00504). Funding for open access charge: Universidad Miguel Hernández.

## Acknowledgments

We thank J. Castelló, J.M. Serrano, and M.J. Ñíguez for their excellent technical assistance, and M. Sendra-Ortolà and I.C. Pomares-Bri for helping in the phenotypic analysis of *api7-1* and some gene constructs. This manuscript was previously published as a preprint at: https://www.biorxiv.org/content/10.1101/2022.05.30.493987v1.

## Conflict of interest

The authors declare that the research was conducted in the absence of any commercial or financial relationships that could be construed as a potential conflict of interest.

## Publisher’s note

All claims expressed in this article are solely those of the authors and do not necessarily represent those of their affiliated organizations, or those of the publisher, the editors and the reviewers. Any product that may be evaluated in this article, or claim that may be made by its manufacturer, is not guaranteed or endorsed by the publisher.
