# Supplementary material for: The Arabidopsis ATP-Binding Cassette E protein ABCE2 is a conserved component of the translation machinery
Source: Front Plant Sci. 2022 Oct 17;13:1009895. doi: 10.3389/fpls.2022.1009895 (PMC9618717; doi:10.3389/fpls.2022.1009895)
Supplement: Supplementary file 4 [file Presentation_1.pdf]

The Arabidopsis ATP-Binding Cassette E protein ABCE2 is a conserved component of the translation machinery

Carla Navarro-Quiles[1], Eduardo Mateo-Bonmatí[1], Héctor Candela[1], Pedro Robles[1], Antonio Martínez-Laborda[2], Yolanda Fernández[3], Jan Šimura[4], Karin Ljung[4], Vicente Rubio[3], María Rosa Ponce[1], and José Luis Micol[1]

[1]Instituto de Bioingeniería, Universidad Miguel Hernández, Campus de Elche, Elche, Spain; [2]Área de Genética, Universidad Miguel Hernández, Campus de Sant Joan, Alicante, Spain; [3]Centro Nacional de Biotecnología, CNB-CSIC, Madrid, Spain; [4]Umeå Plant Science Centre, Department of Forest Genetics and Plant Physiology, Swedish University of Agricultural Sciences, Umeå, Sweden

Supplementary Figures and Tables

Supplementary Material not included in this file

Navarro-Quiles et al_Data Set 1.xlsx

Navarro-Quiles et al_Data Set 2.xlsx

Navarro-Quiles et al_Data Set 2.xlsx

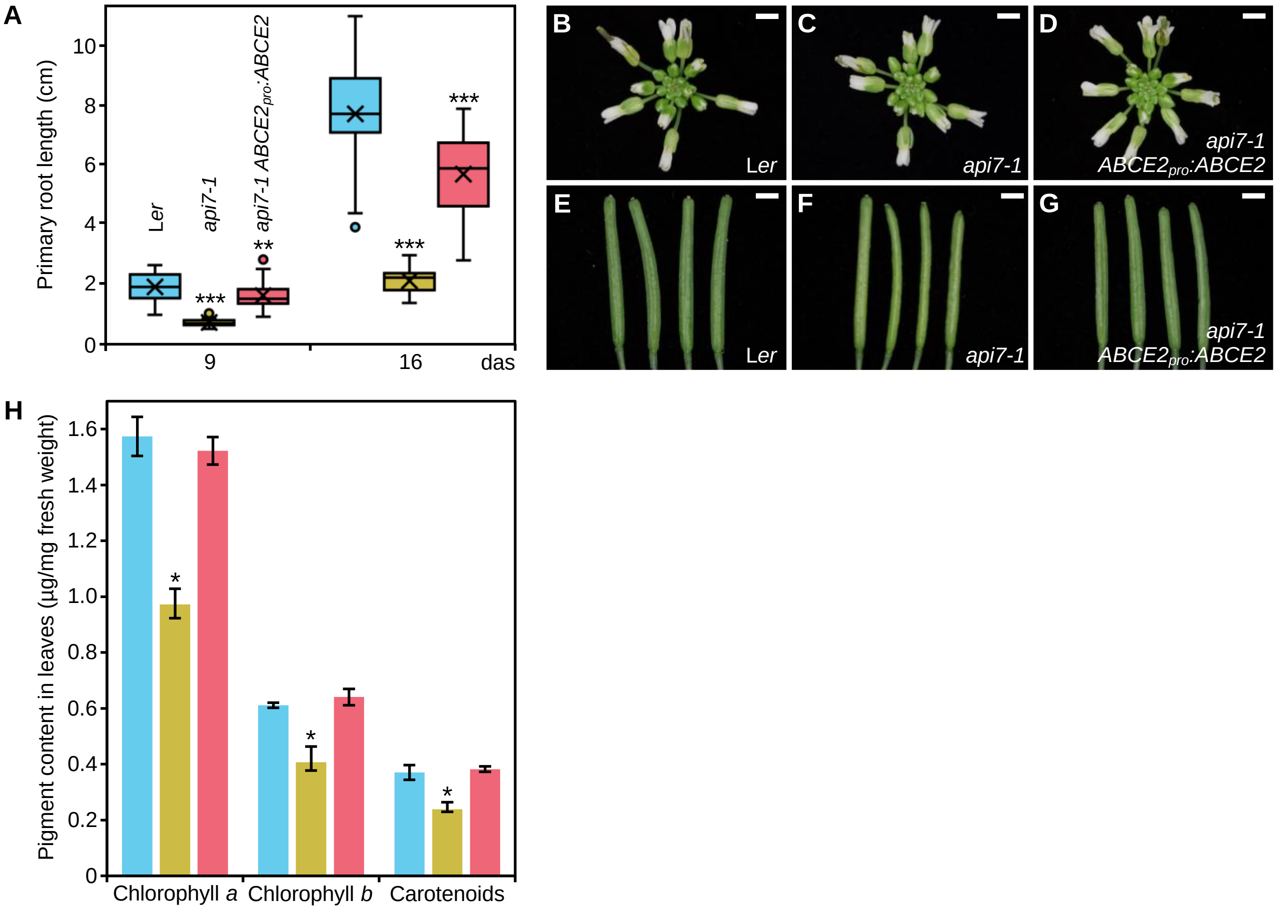

Supplementary Figure S1. Primary root length, inflorescence and silique morphological phenotypes, and pigment content in leaves of L*er*, *api7-1*, and *api7-1 ABCE2$_{pro}$:ABCE2* plants. **(A)** Primary root growth progression between 9 and 16 das in wild-type L*er*, *api7-1* mutant, and *api7-1 ABCE2$_{pro}$:ABCE2* mutant and transgenic rosettes. Boxes are delimited by the first (Q1, lower hinge) and third (Q3, upper hinge) quartiles. Whiskers represent the most extreme data points that are no more than Q3 + 1.5 × IQR or no less than Q1 − 1.5 × IQR, where the interquartile range (IQR) is Q3 − Q1. ×: Mean. —: Median. ○: Outlier. **(B–D)** Inflorescences and **(E–G)** siliques of **(B,E)** L*er*, **(C,F)** *api7-1*, and **(D,G)** *api7-1 ABCE2$_{pro}$:ABCE2* plants. Pictures were taken 40 das. Scale bars indicate 2 mm. **(H)** Chlorophyll *a* and *b*, and carotenoid content in plants of the genotypes mentioned in **(A)** collected 16 das. Median values are shown. Error bars represent median absolute deviation. Asterisks indicate a significant difference with L*er* in **(A)** a Student's *t* test (28 < *n* < 35) or **(H)** a Mann-Whitney *U* test (*n* = 5) (**P* < 0.05, ***P* < 0.01, ****P* < 0.001).

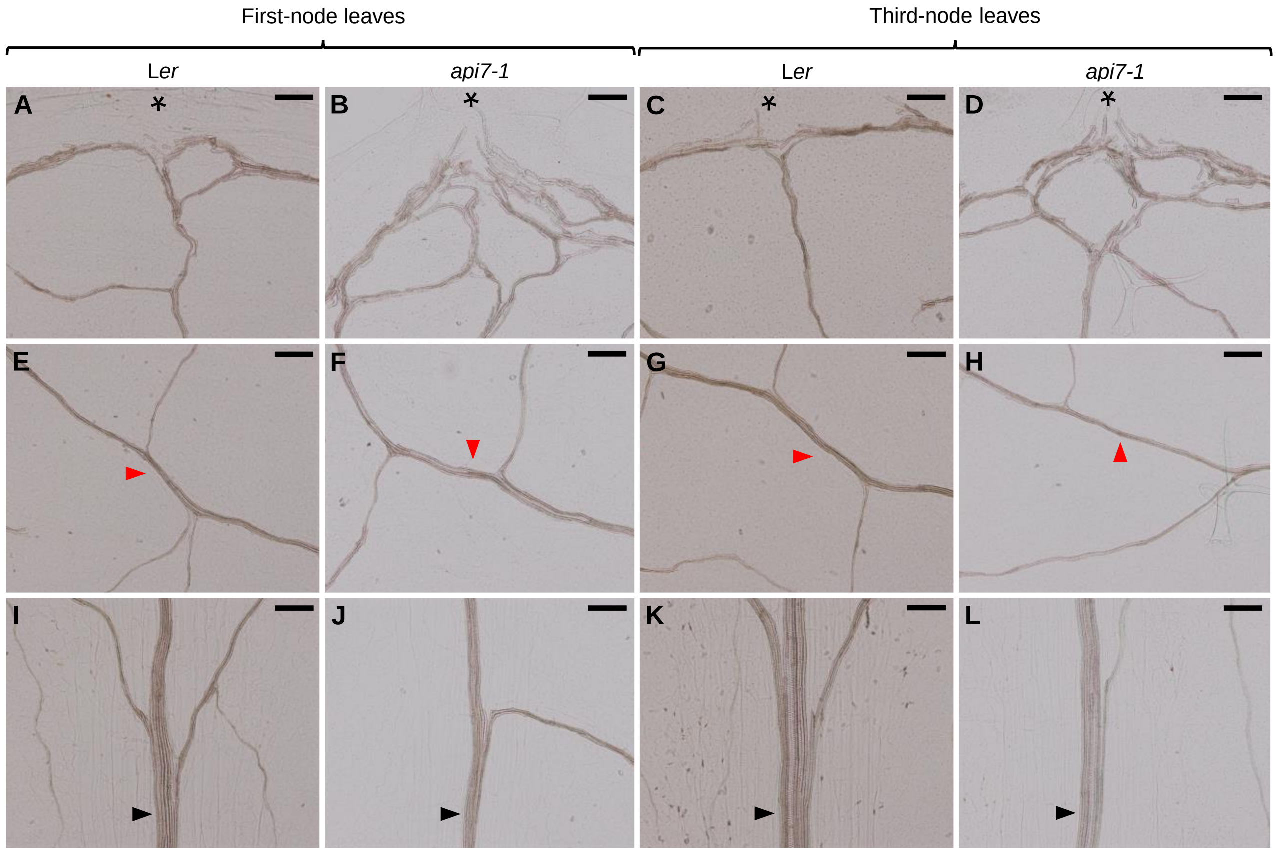

Supplementary Figure S2. Some details of the vascular phenotype of first- and third-node leaves from L*er* and *api7-1* plants. Veins from **(A,C,E,G,I,K)** L*er* and **(B,D,F,H,J,L)** *api7-1* **(A,B,E,F,I,J)** first- and **(C,D,G,H,K,L)** third-node leaves. Venation on **(A–D)** the apical region (an asterisk indicates the most apical region) of the lamina, **(E–H)** a secondary vein (red arrowheads) bifurcating to render tertiary veins, and **(I–L)** the primary vein (black arrowheads), close to the base of the lamina. We observed 6 first- and third-node leaves from L*er* and 18 from *api7-1* with similar vascular phenotypes to the ones shown. Pictures were taken 21 das. Scale bars indicate 100 µm.

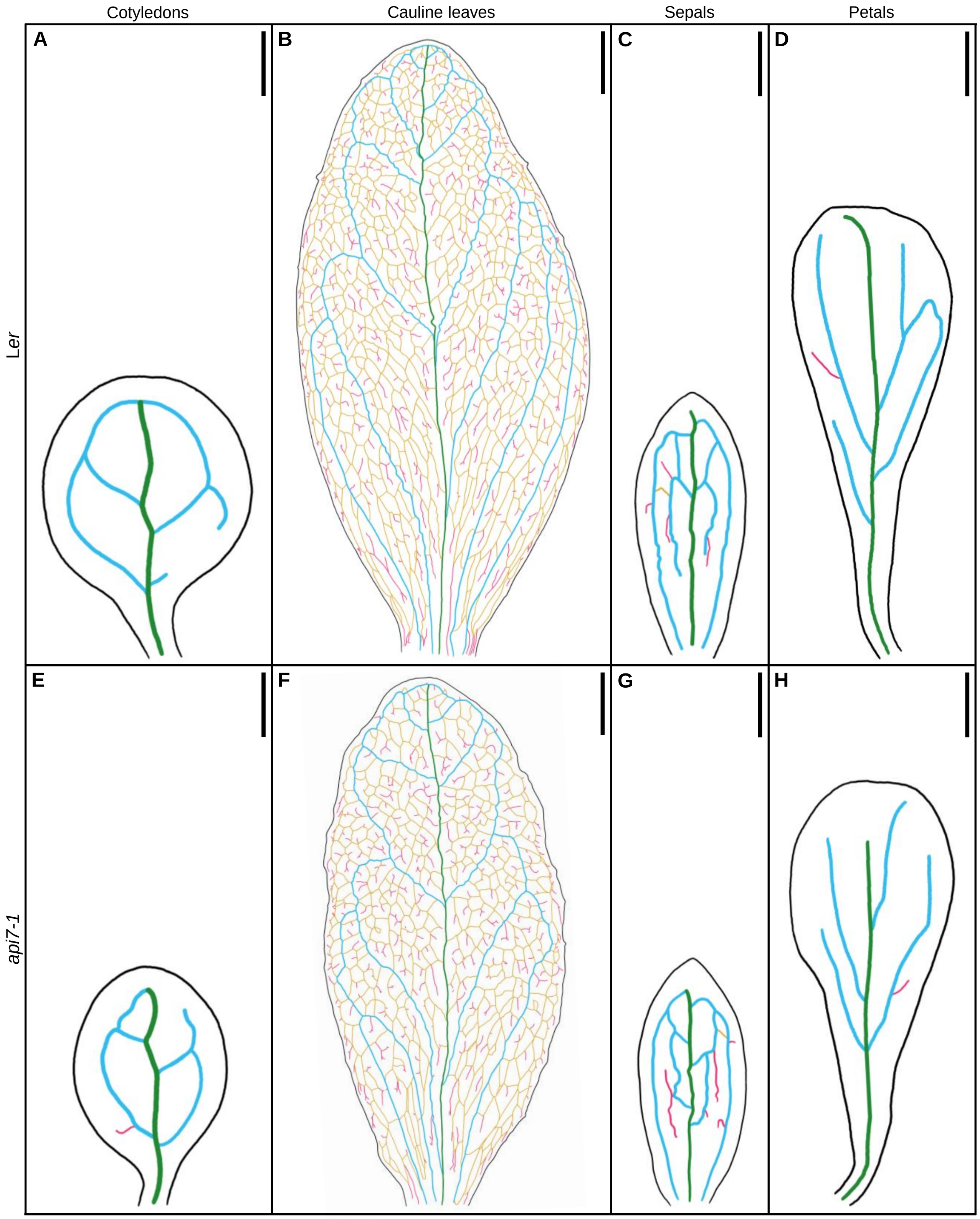

Supplementary Figure S3. Venation pattern of *api7-1* cotyledons, cauline leaves, sepals, and petals. Representative diagrams of **(A,E)** cotyledons, **(B,F)** cauline leaves, **(C,G)** sepals, and **(D,H)** petals from **(A–D)** L*er* and **(E–H)** *api7-1* plants after visualization of 12 samples per organ and genotype. Margins and veins were drawn as described in Figure 2. Organs were collected **(A,E)** 6 and **(B–D,F–H)** 35 das. Scale bars indicate **(A,C–E,G,H)** 0.5 and **(B,F)** 2.5 mm.

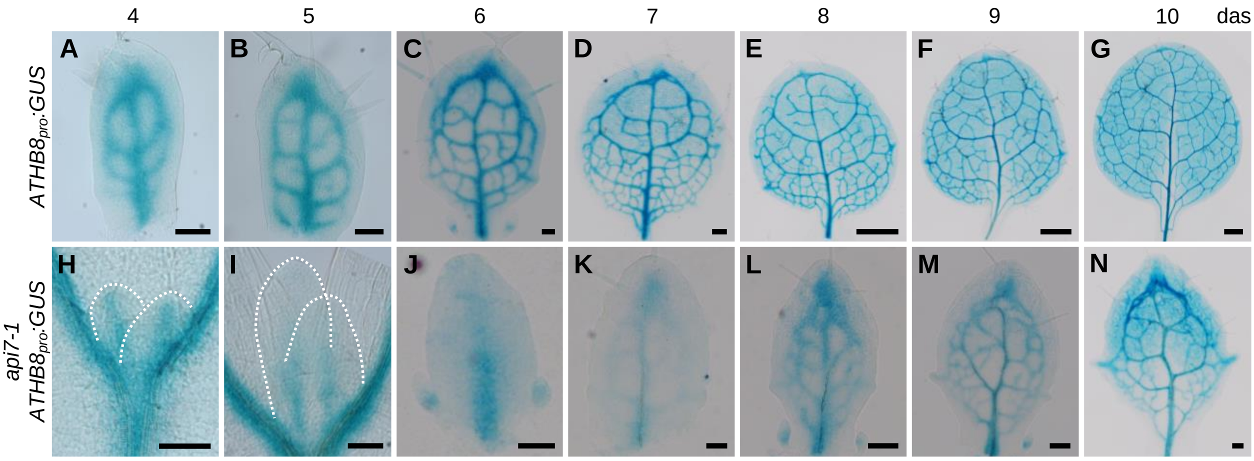

Supplementary Figure S4. Vascularization in *api7-1* leaf primordia. Vascular fate specification is shown as *ATHB8$_{pro}$:GUS* activity at expanding first-node leaf primordia in **(A–G)** L*er* and **(H–N)** *api7-1* backgrounds. First- and second-note leaf primordia have been delinated in **(H)** and **(I)**. Pictures were taken **(A,H)** 4, **(B,I)** 5, **(C,J)** 6, **(D,K)** 7, **(E,L)** 8, **(F,M)** 9, and **(G,N)** 10 das. Scale bars indicate **(A–C,H–J)** 50, **(D,K–N)** 100, and **(E–G)** 500 µm.

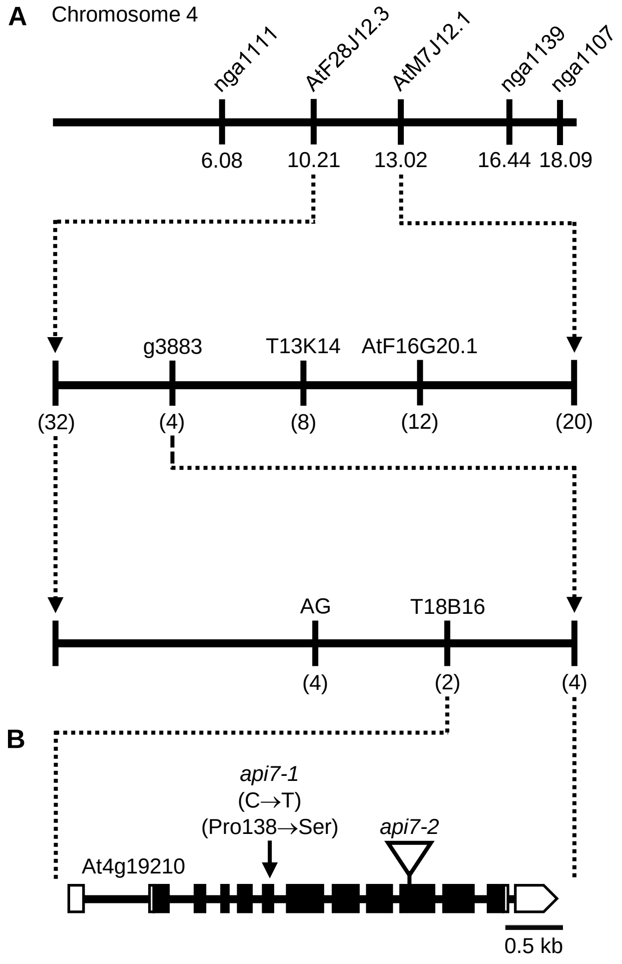

Supplementary Figure S5. Fine mapping by linkage analysis of the *api7-1* mutation. **(A)** A mapping population of 273 F_2 plants derived from an *api7-1* × Col-0 cross allowed us to delimit a candidate region of 123.5 kb in chromosome 4, flanked by the T18B16 and g3883 markers. The names and physical map positions of the molecular markers used for linkage analysis are shown. All values outside parentheses indicate Mb. The number of recombinant chromosomes found (from a total of 546 chromosomes analyzed) is indicated in parentheses. **(B)** Structure of the At4g19210 (*ABCE2*) gene, located within the candidate region, with indication of the nature and position of the *api7* mutations studied in this work. Boxes and lines indicate exons and introns, respectively. White boxes represent UTRs. The arrow indicates the *api7-1* point mutation. The triangle indicates the *api7-2* T-DNA insertion (GABI_509C06).

```
S. solfataricus     1 ----------------MRVAVINYDFCKPDKCNL-ECINFCPVDRSGGKAIELSEIVKGK
P. furiosus         1 ----------------MRIAVIDYDKCNPDKCGHFLCERVCPVNRMGGEAIIIDEE-NYK
C. elegans          1 MSRKGPLIKNNETDVPLRIAIVEKDRCKPKNCGL-ACKRACPVNRQGKQCIVVEAT-STI
S. cerevisiae       1 -----------MSDKNSRIAIVSADKCKPKKCRQ-ECKRSCPVVKTGKLCIEVTPT-SKI
O. sativa           1 -----------MAERLTRIAIVSEDKCKPKKCRQ-ECKKSCPVVKTGKLCIEVTPA-SKL
S. lycopersicum     1 -----------MSDRLTRIAIVSDDKCKPKKCRQ-ECKKSCPVVKTGKLCIEVTVG-SKI
A. thaliana         1 -----------MADRLTRIAIVSSDRCKPKKCRQ-ECKKSCPVVKTGKLCIEVTVG-SKL
C. hirsuta          1 -----------MADRLTRIAIVSSDRCKPKKCRQ-ECKKSCPVVKTGKLCIEVTVA-SKI
D. melanogaster     1 MSR---RKENEEVDKQTRIAIVSDDKCKPKRCRQ-ECKKTCPVVRMGKLCIEVTPT-SKI
H. sapiens          1 -----------MADKLTRIAIVNHDKCKPKKCRQ-ECKKSCPVVRMGKLCIEVTPQ-SKI
O. cuniculus        1 -----------MADKLTRIAIVNHDKCKPKKCRQ-ECKKSCPVVRMGKLCIEVTPQ-SKI
D. rerio            1 -----------MADKNTRIAIVNHDKCKPKKCRQ-ECKKSCPVVRMGKLCIEVSPQ-SKI
consensus           1            . .. .*.*..  *.*.*..*.. .*...***.. *...*...   ...

S. solfataricus    44 PVIYEETCIGCGICVKKCPYEAISIVNLPDELEGEVIHRYKVNGFKLFGLPTPKNNTILG
P. furiosus        44 PIIQEASCTGCGICVHKCPFKAISIVNLPEQLEEDCVHRYGVNAFVLYRLPVVKEGMVVG
C. elegans         59 SQISEILCIGCGICVKKCPYDAIKIINLPANLANETTHRYSQNSFKLHRLPTPRCGEVLG
S. cerevisiae      48 AFISEILCIGCGICVKKCPFDAIQIINLPTNLEAHVTHRYSANSFKLHRLPTPRPGQVLG
O. sativa          48 AFISEELCIGCGICVKKCPFDAIEIINLPKDLEKDTTHRYGPNTFKLHRLPVPRPGQVLG
S. lycopersicum    48 AFISEELCIGCGICVKKCPFEAIQIINLPKDLDKDTTHRYGPNTFKLHRLPVPRPGQVLG
A. thaliana        48 AFISEELCIGCGICVKKCPFEAIQIINLPRDLEKDTTHRYGANTFKLHRLPVPRPGQVLG
C. hirsuta         48 AFISEELCIGCGICVKKCPFEAIQIINLPRDLEKDTTHRYGPNTFKLHRLPVPRPGQVLG
D. melanogaster    56 ASLSEELCIGCGICVKKCPFEAITIINLPSNLEKHTTHRYSKNSFKLHRLPIPRPGEVLG
H. sapiens         48 AWISETLCIGCGICIKKCPFGALSIVNLPSNLEKETTHRYCANAFKLHRLPIPRPGEVLG
O. cuniculus       48 AWISETLCIGCGICIKKCPFGALSIVNLPSNLEKETTHRYCANAFKLHRLPIPRPGEVLG
D. rerio           48 VWISESLCIGCGICIKKCPFGALSIVNLPSNLEKETTHRYCANSFKLHRLPIPRPGEVLG
consensus          61 ....* .*.*****..***..*. *.***  *.....***  *.*.*..**..... ..*

S. solfataricus   104 VLGKNGVGKTTVLKILAGEIIPNFGDPNSKVGKDEVLKRFRGKEIYNYFKELYSNELKIV
P. furiosus       104 IVGPNGTGKSTAVKILAGQLIPNLCG--DNDSWDGVIRAFRGNELQNYFEKLKNGEIRPV
C. elegans        119 LVGTNGIGKSTALKILAGKQKPNLGNFQKEQEWTTIINHFRGSELQNYFTRILEDTLKCV
S. cerevisiae     108 LVGTNGIGKSTALKILAGKQKPNLGRFDDPPEWQEIIKYFRGSELQNYFTKMLEDDIKAI
O. sativa         108 LVGTNGIGKSTALKVLAGKLKPNLGRFKNPPDWQEILTYFRGSELQNYFTRILEDNLKAI
S. lycopersicum   108 LVGTNGIGKSTALKVLAGKLKPNLGRFTNPPDWQEILTHFRGSELQNYFTRILEDNLKAI
A. thaliana       108 LVGTNGIGKSTALKILAGKLKPNLGRFTSPPDWQEILTHFRGSELQNYFTRILEDNLKAI
C. hirsuta        108 LVGTNGIGKSTALKVLAGKLKPNLGRFTNPPDWQEILTHFRGSELQNYFTRILEDNLKAI
D. melanogaster   116 LVGQNGIGKSTALKILAGKQKPNLGKYANPPDWTEILSYFRGSELQNYFTKILEDNLKAL
H. sapiens        108 LVGTNGIGKSTALKILAGKQKPNLGKYDDPPDWQEILTYFRGSELQNYFTKILEDDLKAI
O. cuniculus      108 LVGTNGIGKSTALKILAGKQKPNLGKYDDPPDWQEILTYFRGSELQNYFTKILEDDLKAI
D. rerio          108 LVGTNGIGKSTALKILAGKQKPNLGRFDAPPDWQEILAYFRGSELQNYFTKILEDDLKAI
consensus         121 ..*.**.**.*..*.***. .**....  ........  ***.*..***...... ....

S. solfataricus   164 HKIQYVEYASKFLKGTVNEI---LTKIDER--GKKDEVKELLNMTNLWNKDANILSGGGL
P. furiosus       162 VKPQYVDLIPKAVKGKVIEL---LKKADET--GKLEEVVKALELENVLDREIQHLSGGEL
C. elegans        179 IKPQYVDQIPRAAKGTVEKN---LTRKHDN--DNLNSVIDQMELRGLLDREIDQLSGGEL
S. cerevisiae     168 IKPQYVDNIPRAIKGPVQKVGELLKLRMEKSPEDVKRYIKILQLENVLKRDIEKLSGGEL
O. sativa         168 IKPQYVDHIPKAVQGNVGQV---LDQKDER--GVKAELCVDLELNQVIDRNVGDLSGGEL
S. lycopersicum   168 IKPQYVDHIPKAVQGNVGQV---LDQKDER--DVKEKLCVDLELNQVLDRNVGDLSGGEL
A. thaliana       168 IKPQYVDHIPRAVKGNVGEV---LDQKDER--DKKAELCADLELNQVIDRDVENLSGGEL
C. hirsuta        168 IKPQYVDHIPKAVRGNVGEV---LDQKDER--NKKAELCADLELNQVIDRDVENLSGGEL
D. melanogaster   176 VKPQYVDQIPKAVRGAVGDL---LDKKDER--ELQTKICEMLDLSHIRDREIAQLSGGEL
H. sapiens        168 IKPQYVDQIPKAAKGTVGSI---LDRKDET--KTQAIVCQQLDLTHLKERNVEDLSGGEL
O. cuniculus      168 IKPQYVDQIPKAAKGTVGSI---LDRKDET--KTQAIVCQQLDLTHLKERNVEDLSGGEL
D. rerio          168 VKPQYVDQIPKTVKGSVGAI---LSRKDDT--KTEELVCEQLDLLHLRDRNVEDLSGGEL
consensus         181 .*.***. ......* *. .   *  ...        ..  ...  . .. .  ****.*
```

```
S. solfataricus  219 QRLLVAASLLREADVYIFDEPSSYLDVRERMNMAKAIRELLK-NKYVIVVDHDLIVLDYL
P. furiosus      217 QRVAIAAALLRNATFYFFDEPSSYLDIRQRLNAARAIRRLSEEGKSVLVVEHDLAVLDYL
C. elegans       234 QRFAIAMCCVQKADVYMFDEPSSYLDVKQRLKAAAIIRERVSDTNYVVVVEHDLAVLDYL
S. cerevisiae    228 QRFAIGMSCVQEADVYMFDEPSSYLDVKQRLNAAQIIRSLLAPTKYVICVEHDLSVLDYL
O. sativa        223 QRFAIAVVAVQNAEIFMFDEPSSYLDVKQRLKAAQVVRSLIRPNSYVIVVEHDLSVLDYL
S. lycopersicum  223 QRFAIAVVAIQNAEIYMFDEPSSYLDVKQRLKAAQVVRSLIRPNSYVIVVEHDLSVLDYL
A. thaliana      223 QRFAIAVVAIQNAEIYMFDEPSSYLDVKQRLKAAQVVRSLIRPNSYVIVVEHDLSVLDYL
C. hirsuta       223 QRFAIAVVAIQNAEIYMFDEPSSYLDVKQRLKAAQVVRSLIRPNSYVIVVEHDLSVLDYL
D. melanogaster  231 QRFAIAMVCIQNADIFMFDEPSSYLDVKQRLNAALTIRSLLHPTKFIIVVEHDLSVLDYL
H. sapiens       223 QRFACAVVCIQKADIFMFDEPSSYLDVKQRLKAAITIRSLINPDRYIIVVEHDLSVLDYL
O. cuniculus     223 QRFACAVVCIQKADIFMFDEPSSYLDVKQRLKAAITIRSLINPDRYIIVVEHDLSVLDYL
D. rerio         223 QRFACAVVCIQRADIFMFDEPSSYLDVKQRLRAAITIRSLISPDRYIIVVEHDLSVLDYL
consensus        241 **...... .. *....*********...*...*  .*... .  ....*.***.*****

S. solfataricus  278 TDLIHIIYGESSVYGRVSKSYAARVGINNFLKGYLPAENMKIRPDEIKFMLKEVSD-LDL
P. furiosus      277 SDIIHVVYGEPGVYGIFSQPKGTRNGINEFLRGYLKDENVRFRPYEIKFTKTGER----V
C. elegans       294 SDFICCLYGVPGVYGVVTLPSGVREGINMFLEGFIRTENMRFRESKLSFKTSEQQ----E
S. cerevisiae    288 SDFVCIIYGVPSVYGVVTLPASVREGINIFLDGHIPAENLRFRTEALQFRIADATEDL-Q
O. sativa        283 SDFICCLYGKPGAYGVVTLPFSVREGINIFLAGFVPTENLRFRDESLTFKIAET-QESAE
S. lycopersicum  283 SDFICCLYGKPGAYGVVTLPFSVREGINIFLAGFVPTENLRFRDESLTFKVAETPQEAAE
A. thaliana      283 SDFICCLYGKPGAYGVVTLPFSVREGINIFLAGFVPTENLRFRDESLTFKVAETPQESAE
C. hirsuta       283 SDFICCLYGKPGAYGVVTLPFSVREGINIFLAGFVPTENLRFRDESLTFKVAETPQESAE
D. melanogaster  291 SDFICCLYGVPGCYGVVTMPFSVREGINIFLDGFVPTENMRFRTESLTFKVSESAT--EE
H. sapiens       283 SDFICCLYGVPSAYGVVTMPFSVREGINIFLDGYVPTENLRFRDASLVFKVAETAN--EE
O. cuniculus     283 SDFICCLYGVPSAYGVVTMPFSVREGINIFLDGYVPTENLRFRDASLVFKVAETAN--EE
D. rerio         283 SDFICCLYGVPSAYGVVTMPFSVREGINIFLDGYVPTENLRFRETSLVFKVAETAA--EE
consensus        301 .*.....** .  **........*.***.** *....**...*. .. *.....     .

S. solfataricus  337 SKDLKTKMKWTKIIKKLGDEQLVVDNGEAKEGEIIGILGPNGIGKTTFARILVGEITADE
P. furiosus      333 EIERETLVTYPRLVKDYGSFRLEVEPGEIKKGEVIGIVGPNGIGKTTFVKMLAGVEEPTE
C. elegans       350 DIKRTGNIKYPSMSKTLGNFHLDVEAGDFSDSEIIVMLGENGTGKTTMIKMMAGSLKPED
S. cerevisiae    347 NDSASRAFSYPSLKKTQGDFVLNVEEGEFSDSEILVMMGENGTGKTTLIKLLAGALKPDE
O. sativa        342 EIETYQRYKYPTMSKTQGNFKLTVVEGEFTDSQIVVMLGENGTGKTTFIRMLAGLLKPDT
S. lycopersicum  343 EIESYARYRYPTMTKTQGNFKLKVSEGEFTDSQIVVMLGENGTGKTTFIRMLAGLLKPDV
A. thaliana      343 EIQSYARYKYPTMTKTQGNFRLRVSEGEFTDSQIIVMLGENGTGKTTFIRMLAGLLKPDD
C. hirsuta       343 EVQSYARYKYPTMSKTQGNFRLRVSEGEFTDSQIIVMLGENGTGKTTFIRMLAGLLKPDE
D. melanogaster  349 EIKRMNHYVYPAMVKTLGKFELTVEKGHFSDSEILVLLGENGTGKTTFIRMLAGNLQPDG
H. sapiens       341 EVKKMCMYKYPGMKKKMGEFELAIVAGEFTDSEIMVMLGENGTGKTTFIRMLAGRLKPDE
O. cuniculus     341 EVKKMCMYKYPGMKKKMGEFELAIVAGEFTDSEIMVMLGENGTGKTTFIRMLAGRLKPDE
D. rerio         341 EVKKLCRYQYPNMKKSMGEFTLTITEGEFTDSEIMVMLGENGTGKTTFIRMLAGGLKPDG
consensus        361 ..      .... . *. * * *  .  *..........*.**.****......*  .....

S. solfataricus  397 ----GSVTPEKQILSYKPQRIFPNYDGTVQQYLENASKDALSTSSWFFEEVTKRLNLHRL
P. furiosus      393 ----GKIEW-DLTVAYKPQYIKADYEGTVYELLSKIDASKLN-SNFYKTELLKPLGIIDL
C. elegans       410 E---NTELP-HVSISYKPQKISPKSETTVRFMLHDKIQNMYE-HPQFKTDVMNPLMMEQL
S. cerevisiae    407 ----GQDIP-KLNVSMKPQKIAPKFPGTVRQLFFKKIRGQFL-NPQFQTDVVKPLRIDDI
O. sativa        402 VEGTEIEIP-EFNVSYKPQKISPKFQHSVRHLLHQKIRDSYT-HPQFVSDVMKPLQIEQL
S. lycopersicum  403 VEGSDIEMP-EFNVSYKPQKISPKFQSTVRHLLHQKIRDSYQ-HPQFCSDVMKPLQIEQL
A. thaliana      403 TEGPDREIP-EFNVSYKPQKISPKFQNSVRHLLHQKIRDSYM-HPQFMSDVMKPLQIEQL
C. hirsuta       403 TEGPDREIP-EFNVSYKPQKISPKFQNSVRHLLHQKIRDSYM-HPQFVSDVMKPLQIEQL
D. melanogaster  409 ----EVELP-MLNISYKPQKISPKFQNHVRHLLHDKIRDAYV-HPQFIADVMKPMKIEEI
H. sapiens       401 ----GGEVP-VLNVSYKPQKISPKSTGSVRQLLHEKIRDAYT-HPQFVTDVMKPLQIENI
O. cuniculus     401 ----GGEVP-VLNVSYKPQKISPKSTGSVRQLLHEKIRDAYT-HPQFVTDVMKPLQIENI
D. rerio         401 ----GGDVP-ILNVSYKPQKISPKFKGSVRALLHDKIRDAYT-HPQFVTDVMKPMQIESI
consensus        421        ...   ....***.*....  .*. ... .... .  .... .......... .
```

```
S. solfataricus  453 LESNVNDLSGGELQKLYIAATLAKEADLYVLDEPSSYLDVEERYIVAKAIKRVTRERKAV
P. furiosus      447 YDREVNELSGGELQRVAIAATLLRDADIYLLDEPSAYLDVEQRLAVSRAIRHLMEKNEKT
C. elegans       465 LDRNVKELSGGELQRVALALCLGKTASLYLIDEPSAYLDSEQRLHAAKVIKRFIMHAKKT
S. cerevisiae    461 IDQEVQHLSGGELQRVAIVLALGIPADIYLIDEPSAYLDSEQRIICSKVIRRFILHNKKT
O. sativa        460 MDQEVINLSGGELQRVAICLCLGKPADIYLIDEPSAYLDSEQRIVASKVIKRFILHAKKT
S. lycopersicum  461 MDQEVVNLSGGELQRVALALCLGKPADIYLIDEPSAYLDSEQRIVASKVIKRFILHAKKT
A. thaliana      461 MDQEVVNLSGGELQRVALTLCLGKPADIYLIDEPSAYLDSEQRIVASKVIKRFILHAKKT
C. hirsuta       461 MDQEVINLSGGELQRVALALCLGKPADIYLIDEPSAYLDSEQRIVASKVIKRFILHAKKT
D. melanogaster  463 MDQEVQNLSGGELQRVALVLCLGKPADVYLIDEPSAYLDSEQRLVAAKVIKRYILHAKKT
H. sapiens       455 IDQEVQTLSGGELQRVALALCLGKPADVYLIDEPSAYLDSEQRLMAARVVKRFILHAKKT
O. cuniculus     455 IDQEVQTLSGGELQRVALALCLGKPADVYLIDEPSAYLDSEQRLMAARVVKRFILHAKKT
D. rerio         455 IDQDVQNLSGGELQRVALALCLGKPADVYLIDEPSAYLDSEQRLMAARVIKRFILHAKKT
consensus        481 ....*  *******.......*...*..*..****.***.*.*... .............

S. solfataricus  513 TFIIDHDLSIHDYIADRIIVFKGEPEKAGLATSPVTLKTGMNEFLRELEVTFRRDAETGR
P. furiosus      507 ALVVEHDVLMIDYVSDRLMVFEGEPGKYGRALPPMGMREGMNRFLASIGITFRRDPDTGR
C. elegans       525 AFVVEHDFIMATYLADRVVVFEGQPSVKCTACKPQSLLEGMNRFLKMLDITFRRDQETYR
S. cerevisiae    521 AFIVEHDFIMATYLADKVIVFEGIPSKNAHARAPESLLTGCNRFLKNLNVTFRRDPNSFR
O. sativa        520 AFIVEHDFIMATYLADKVIVYEGRPSIDCTANAPQSLVSGMNKFLSHLDITFRRDPTNYR
S. lycopersicum  521 AFVVEHDFIMATYLADRVIVYEGTPSIDCVANAPQSLLTGMNLFLSHLNITFRRDPTNYR
A. thaliana      521 AFVVEHDFIMATYLADRVIVYEGQPSIDCTANCPQSLLSGMNLFLSHLNITFRRDPTNFR
C. hirsuta       521 AFVVEHDFIMATYLADRVIVYEGQPSIDCTANCPQSLLSGMNLFLSHLNITFRRDPTNFR
D. melanogaster  523 GFVVEHDFIMATYLADRVIVIEGQPSVKTTAFSPQSLLNGMNRFLELLGITFRRDPNNFR
H. sapiens       515 AFVVEHDFIMATYLADRVIVFDGVPSKNTVANSPQTLLAGMNKFLSQLEITFRRDPNNYR
O. cuniculus     515 AFVVEHDFIMATYLADRVIVFDGVPSKNTVANSPQTLLAGMNKFLSQLEITFRRDPNNYR
D. rerio         515 AFVVEHDFIMATYLADRVIVFDGIPSRTTNANAPQTLLAGMNKFLAQLEITFRRDPNNFR
consensus        541 .....**.....*..*...*..* *.    *  *.... *.*.**  . .*****. ..*

S. solfataricus  573 PRVNKIGSYLDRVQKERGDYYSMVLSTQ-
P. furiosus      567 PRANKEGSVKDREQKEKGEYYYIA-----
C. elegans       585 PRINKLDSVKDVDQKKSGQFFFLDDN---
S. cerevisiae    581 PRINKLDSQMDKEQKSSGNYFFLDNTGI-
O. sativa        580 PRINKLDSTKDREQKSAGSYYYLDD----
S. lycopersicum  581 PRINKLESTKDREQKSAGSYYYLDD----
A. thaliana      581 PRINKLESTKDREQKSAGSYYYLDD----
C. hirsuta       581 PRINKLESTKDREQKSAGSYYYLDD----
D. melanogaster  583 PRINKNNSVKDTEQKRSGQFFFLEDEACN
H. sapiens       575 PRINKLNSIKDVEQKKSGNYFFLDD----
O. cuniculus     575 PRINKLNSIKDVEQKKSGNYFFLDD----
D. rerio         575 PRINKLNSIKDVEQKKSGNYFFLDD----
consensus        601 **.**. * .* .**  * ......
```

Supplementary Figure S6. Sequence conservation among ABCE orthologs. Multiple sequence alignment of full-length ABCE proteins from the archaea *Saccharolobus solfataricus* (Q980K5) and *Pyrococcus furiosus* (I6V0C7), and the eukaryotes *Caenorhabditis elegans* (Q9U2K8), *Saccharomyces cerevisiae* (Q03195), *Oryza sativa* (A0A0P0Y344), *Solanum lycopersicum* (A0A3Q7H7H5), *Arabidopsis thaliana* (At4g19210), *Cardamine hirsuta* (L7VNS9), *Drosophila melanogaster* (Q9VSS1), *Homo sapiens* (P61221), *Oryctolagus cuniculus* (G1SG72), and *Danio rerio* (Q6TNW3). Full-length protein sequences were obtained from UniProt (https://www.uniprot.org/), except that of *Arabidopsis thaliana*, which was obtained from The Arabidopsis Information Resource (TAIR; https://www.arabidopsis.org/). The alignment was obtained with Clustal Omega 1.2.4 with default settings, and shaded with BOXSHADE with output format RTF_new. Identical and similar residues across at least eight out of the twelve sequences are shaded in black and gray, respectively. Asterisks and dots indicate identical and similar residues, respectively. Numbers indicate residue positions. The conserved Pro138 residue, which is replaced by Ser in the *api7-1* mutant, is highlighted in red.

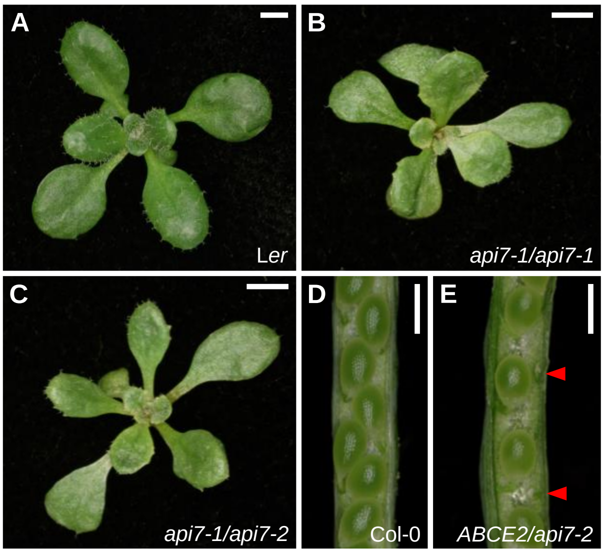

Supplementary Figure S7. *api7-2* is a lethal allele of *ABCE2*. **(A–C)** Rosettes from **(A)** L*er*, **(B)** *api7-1/api7-1*, and **(C)** *api7-1/api7-2* plants. **(D,E)** Dissected immature siliques from **(D)** Col-0 and **(E)** *ABCE2/api7-2* plants. Red arrowheads indicate aborted seeds. Pictures were taken **(A–C)** 16 and **(D,E)** 57 das. Scale bars indicate **(A–C)** 2 mm, and **(D,E)** 500 µm.

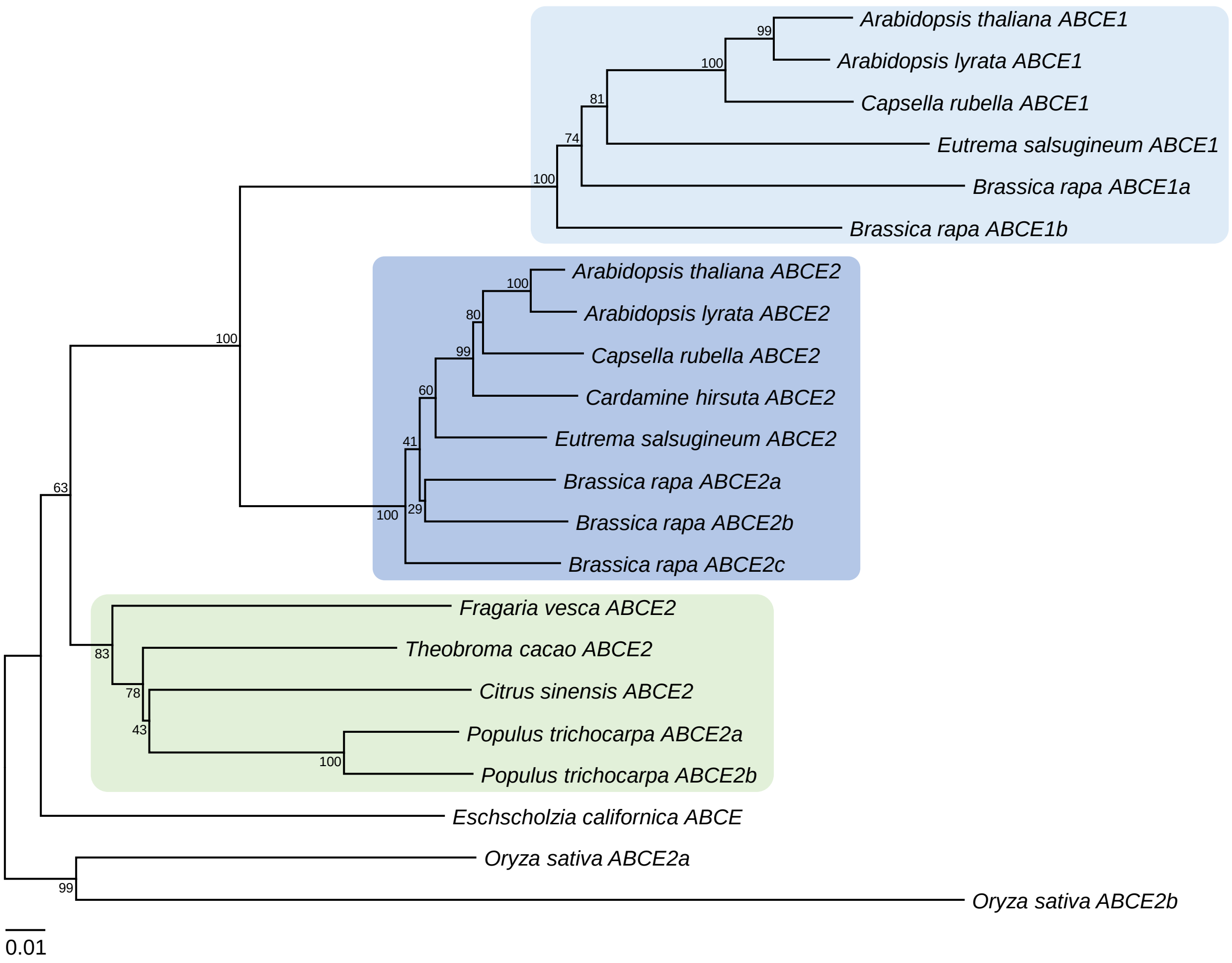

Supplementary Figure S8. Phylogenetic analysis of some Rosidae *ABCE* genes. Rectangles indicate Brassicaceae *ABCE1* (clear blue), *ABCE2* (dark blue), and other rosid *ABCE2* (green) genes. *Eschscholzia californica* and *Oryza sativa ABCE* sequences were used as outgroups. Multiple *ABCE1* or *ABCE2* genes from *Brassica rapa*, *Populus trichocarpa*, and *Oryza sativa* are distinguished with arbitrarily given *a*, *b*, and *c* designations. Refer to Supplementary Table S4 to see the NCBI Nucleotide codes of the sequences used during the multiple sequence alignment. The phylogenetic tree was obtained using the Neighbor-Joining method. All positions containing gaps and missing data were eliminated (complete deletion option). The percentage of replicate trees in which the associated taxa clustered together in the bootstrap test (1000 replicates) are shown next to the branches. The tree was rooted on the midpoint. The scalebar indicates the evolutionary distance as the number of base substitutions per site, and was computed using the Tamura 3-parameter method.

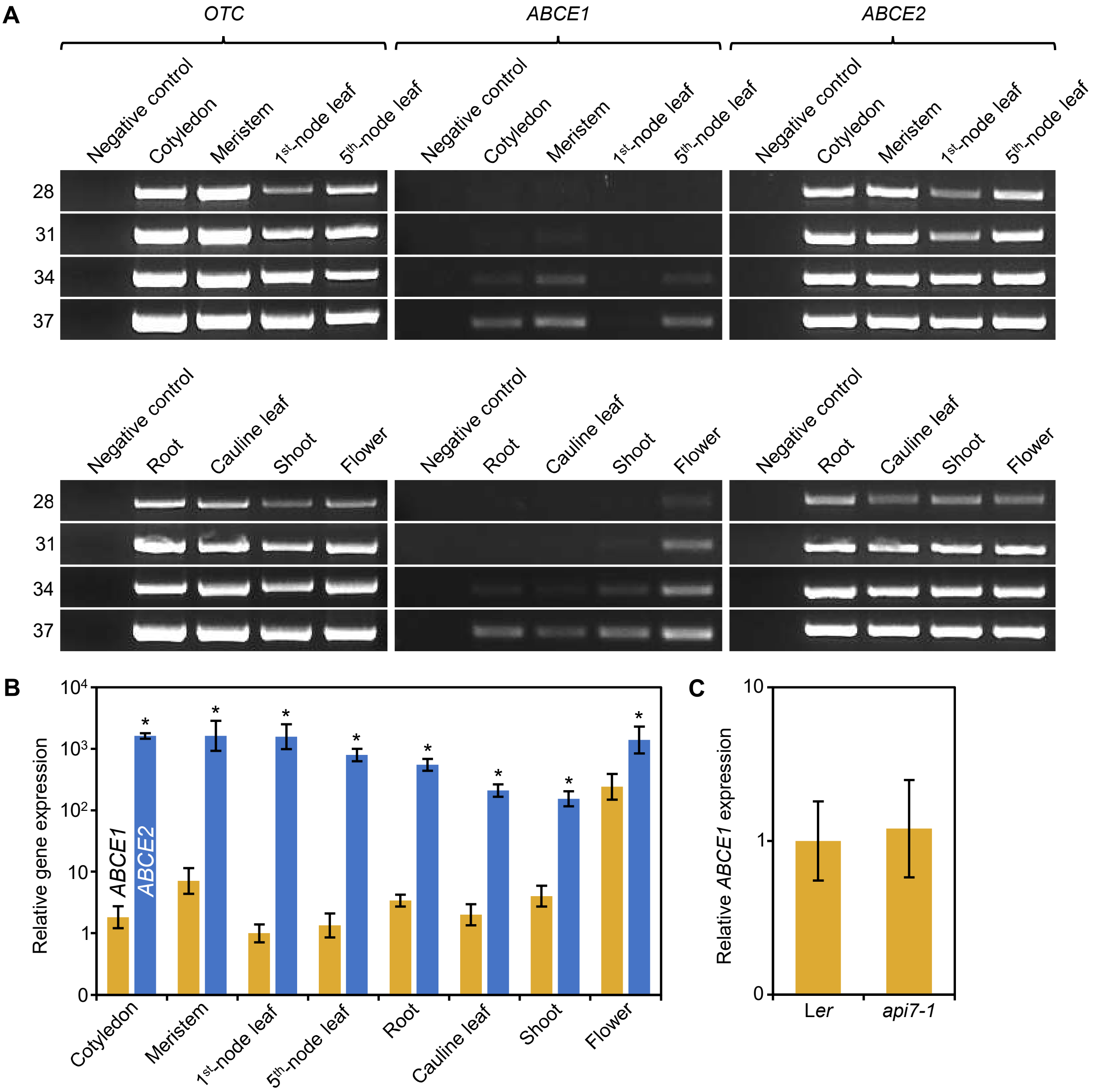

Supplementary Figure S9. *ABCE1* and *ABCE2* expression analyses. **(A)** Semiquantitative and **(B)** quantitative PCR analyses of *ABCE1* and *ABCE2* expression in Col-0 plants. **(A)** The domestic *OTC* gene was used as a control. Negative control samples had no template. The PCR products were visualized after 28, 31, 34, and 37 amplification cycles. **(A,B)** Samples were collected 7 (cotyledons), 14 (meristems, first- and fifth-node leaves, and roots), or 28 (cauline leaves, shoots, and flowers) das. **(C)** *ABCE1* expression in wild-type L*er* and mutant *api7-1* first-node leaves. Samples were collected 14 das. *ABCE1* expression levels in **(B)** first-node leaves and **(C)** L*er* were used as the reference value. **(B,C)** Error bars indicate the interval delimited by $2^{-(\Delta\Delta C_T \pm SD)}$, where SD is the standard deviation of the $\Delta\Delta C_T$ values. Note that the relative gene expression levels are in a logarithmic scale. Asterisks indicate values significantly different between *ABCE1* and *ABCE2* in a Mann-Whitney *U* test (*$P < 0.001$). Three different biological replicates were analyzed in triplicate.

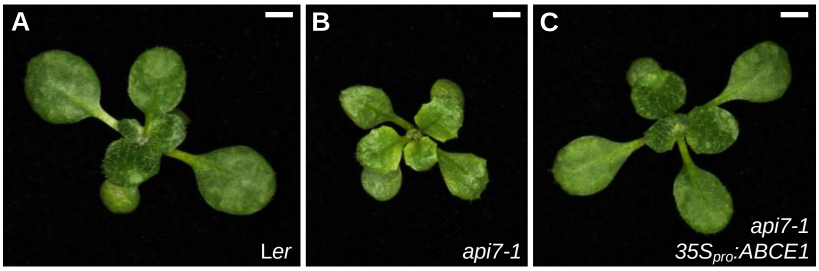

Supplementary Figure S10. The *35S$_{pro}$:ABCE1* transgene restores the wild-type phenotype in *api7-1* plants. Rosettes from **(A)** L*er*, **(B)** *api7-1*, and **(C)** *35S$_{pro}$:ABCE1 api7-1* plants. Pictures were taken 14 das. Scale bars indicate 2 mm.

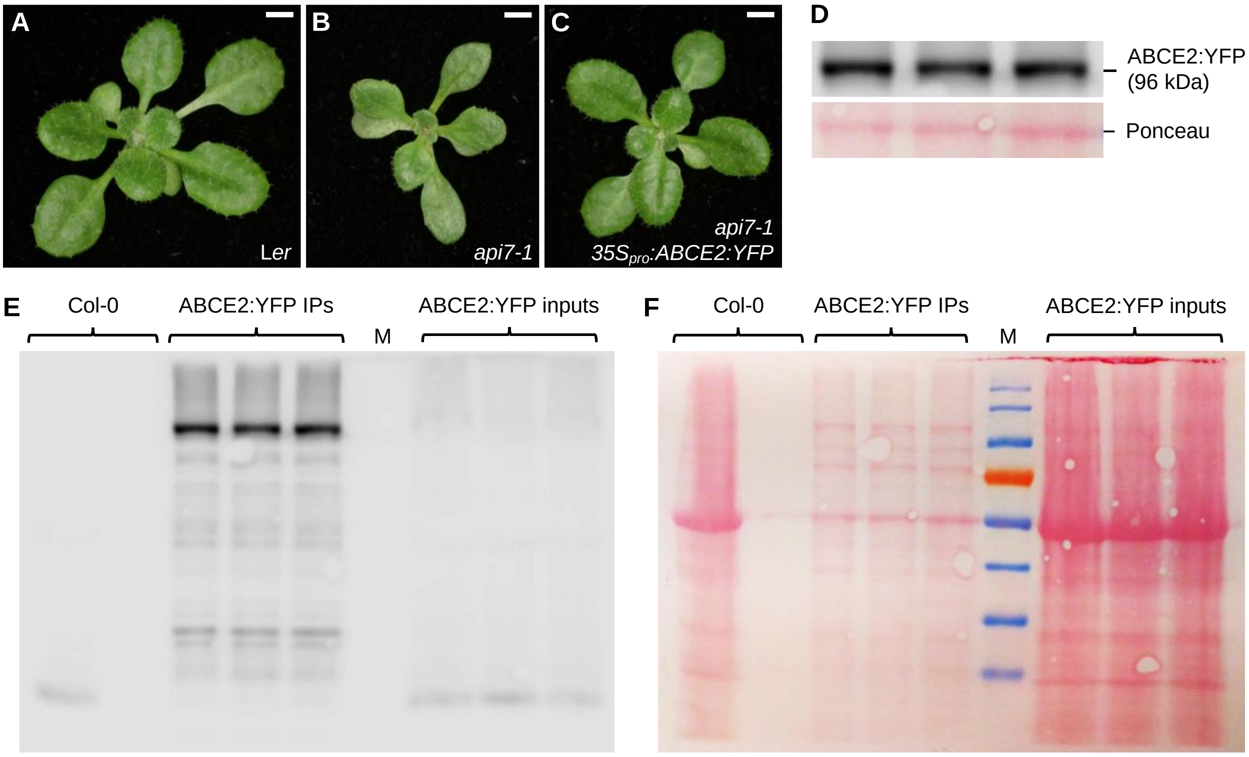

Supplementary Figure S11. The *35S$_{pro}$:ABCE2:YFP* transgene fully restores the wild-type phenotype in *api7-1* plants. **(A–C)** Rosettes from **(A)** L*er*, **(B)** *api7-1*, and **(C)** *api7-1 35S$_{pro}$:ABCE2:YFP* plants. Pictures were taken 16 das. Scale bars indicate 2 mm. **(D–F)** The ABCE2:YFP fusion protein was detected from three independent immunoprecipitates in a western blot probed against GFP. Immunoprecipitates were obtained by immunoprecipitation with anti-GFP magnetic beads of whole-protein extracts from *api7-1 35S$_{pro}$:ABCE2:YFP* plants collected 10 das. A band from the Ponceau staining of the membrane is shown as a loading control. Full pictures of the detection of **(E)** ABCE2:YFP and **(F)** the membrane stained with Ponceau, from the whole-protein extracts previous to immunoprecipitation (inputs) and the immunoprecipitated samples (IPs). A protein extract from wild-type Col-0 seedlings was used as a control: input and immunoprecipitation were loaded in the left and right lanes, respectively. M: EZ-Run Prestained *Rec* Protein Ladder (Thermo Fisher Scientific, Fisher BioReagents) molecular weight marker.

At4g19210 (ABCE2; 62%)

MADRLTRIAIVSSDRCKPKKCRQECKKSCPVVKTGKLCIEVTVGSKLAFISEELCIGCGICVKKCPFEAIQIINLPRDL
EKDTTHRYGANTFKLHRLPVPRPGQVLGLVGTNGIGKSTALKILAGKLKPNLGRFTSPPDWQEILTHFRGSELQNYFTR
ILEDNLKAIIKPQYVDHIPRAVKGNVGEVLDQKDERDKKAELCADLELNQVIDRDVENLSGGELQRFAIAVVAIQNAEI
YMFDEPSSYLDVKQRLKAAQVVRSLLRPNSYVIVVEHDLSVLDYLSDFICCLYGKPGAYGVVTLPFSVREGINIFLAGF
VPTENLRFRDESLTFKVAETPQESAEEIQSYARYKYPTMTKTQGNFRLRVSEGEFTDSQIIVMLGENGTGKTTFIRMLA
GLLKPDDTEGPDREIPEFNVSYKPQKISPKFQNSVRHLLHQKIRDSYMHPQFMSDVMKPLQIEQLMDQEVVNLSGGELQ
RVALTLCLGKPADIYLIDEPSAYLDSEQRIVASKVIKRFILHAKKTAFVVEHDFIMATYLADRVIVYEGQPSIDCTANC
PQSLLSGMNLFLSHLNITFRRDPTNFRPRINKLESTKDREQKSAGSYYYLDD

At3g13640 (ABCE1; 11%)

MSDRLTRIAIVSEDRCKPKKCRQECKKSCPVVKTGKLCIEVGSTSKSAFISEELCIGCGICVKKCPFEAIQIINLPKDL
AKDTTHRYGANGFKLHRLPIPRPGQVLGLVGTNGIGKSTALKILAGKLKPNLGRFNTPPDWEEILTHFRGSELQSYFIR
VVEENLKTAIKPQHVDYIKEVVRGNLGKMLEKLDERGLMEEICADMELNQVLEREARQVSGGELQRFAIAAVFVKKADI
YMFDEPSSYLDVRQRLKAAQVIRSLLRHDSYVIVVEHDLSVLDYLSDFVCCLYGKPGAYGVVTLPFSVREGINVFLAGF
IPTENLRFRDESLTFRVSETTQENDGEVKSYARYKYPNMTKQLGDFKLEVMEGEFTDSQIIVMLGENGTGKTTFIRMLA
GAFPREEGVQSEIPEFNVSYKPQGNDSKRECTVRQLLHDKIRDACAHPQFMSDVIRPLQIEQLMDQVVKTLSGGEKQRV
AITLCLGKPADIYLIDEPSAHLDSEQRITASKVIKRFILHAKKTAFIVEHDFIMATYLADRVIVYEGQPAVKCIAHSPQ
SLLSGMNHFLSHLNITFRRDPTNFRPRINKLESIKDKEQKTAGSYYYLDD

At4g11420 (eIF3a; 32%)

MANFAKPENALKRADELINVGQKQDALQALHDLITSKRYRAWQKPLEKIMFKYLDLCVDLKRGRFAKDGLIQYRIVCQQ
VNVSSLEEVIKHFLHLATDKAEQARSQADALEEALDVDDLEADRKPEDLQLSIVSGEKGKDRSDRELVTPWFKFLWETY
RTVLEILRNNSKLEALYAMTAHKAFQFCKQYKRTTEFRRLCEIIRNHLANLNKYRDQRDRPDLSAPESLQLYLDTRFDQ
LKVATELGLWQEAFRSVEDIYGLMCMVKKTPKSSLLMVYYSKLTEIFWISSSHLYHAYAWFKLFSLQKNFNKNLSQKDL
QLIASSVVLAALSIPPFDRAQSASHMELENEKERNLRMANLIGFNLEPKFEGKDMLSRSALLSELVSKGVLSCASQEVK
DLFHVLEHEFHPLDLGSKIQPLLEKISKSGGKLSSAPSLPEVQLSQYVPSLEKLATLRLLQQVSKIYQTIRIESLSQLV
PFFQFSEVEKISVDAVKNNFVAMKVDHMKGVVIFGNLGIESDGLRDHLAVFAESLSKVRAMLYPVPSKASKLAGVIPNL
ADTVEKEHKRLLARKSIIEKRKEDQERQQLEMEREEEQKRLKLQKLTEEAEQKRLAAELAERRKQRILREIEEKELEEA
QALLEETEKRMKKGKKKPLLDGEKVTKQSVKERALTEQLKERQEMEKKLQKLAKTMDYLERAKREEAAPLIEAAYQRRL
VEEREFYEREQQREVELSKERHESDLKEKNRLSRMLGNKEIFQAQVISRRQAEFDRIRTEREERISKIIREKKQERDIK
RKQIYYLKIEEERIRKLQEEEEARKQEEAERLKKVEAERKANLDKAFEKQRQREIELEEKSRREREELLRGTNAPPARL
AEPTVTPVGTTAPAAAAAAAGAPAAPYVPKWKRQTTEVSGPSAPTSSETDRRSNRGPPPGDDHWGSNRGAAQNTDRWTS
NRERSGPPAEGGDRWGSGPRGSDDRRSTFGSSRPRPTQR

At3g56150 (eIF3c; 14%)

MTSRFFTQVGSESEDESDYEVEVNEVQNDDVNNRYLQSGSEDDDDTDTKRVVKPAKDKRFEEMTYTVDQMKNAMKINDW
VSLQENFDKVNKQLEKVMRITEAVKPPTLYIKTLVMLEDFLNEALANKEAKKKMSTSNSKALNSMKQKLKKNNKLYEDD
INKYREAPEVEEEKQPEDDDDDDDDDDEVEDDDDSSIDGPTVDPGSDVDEPTDNLTWEKMLSKKDKLLEKLMNKDPKEI
TWDWVNKKFKEIVAARGKKGTARFELVDQLTHLTKIAKTPAQKLEILFSVISAQFDVNPGLSGHMPINVWKKCVLNMLT
ILDILVKYSNIVVDDTVEPDENETSKPTDYDGKIRVWGNLVAFLERVDTEFFKSLQCIDPHTREYVERLRDEPMFLALA
QNIQDYFERMGDFKAAAKVALRRVEAIYYKPQEVYDAMRKLAELVEEEEETEEAKEESGPPTSFIVVPEVVPRKPTFPE
SSRAMMDILVSLIYRNGDERTKARAMLCDINHHALMDNFVTARDLLLMSHLQDNIQHMDISTQILFNRTMAQLGLCAFR
AGMITESHSCLSELYSGQRVRELLAQGVSQSRYHEKTPEQERMERRRQMPYHMHLNLELLEAVHLICAMLLEVPNMAAN
SHDAKRRVISKNFRRLLEISERQAFTAPPENVRDHVMAATRALTKGDFQKAFEVLNSLEVWRLLKNRDSILDMVKDRIK
EEALRTYLFTYSSSYESLSLDQLAKMFDVSEPQVHSIVSKMMINEELHASWDQPTRCIVFHEVQHSRLQSLAFQLTEKL
SILAESNERAMESRTGGGGLDLSSRRRDNNQDYAGAASGGGGYWQDKANYGQGRQGNRSGYGGGRSSGQNGQWSGQNRG
GGYAGRVGSGNRGMQMDGSSRMVSLNRGVRT

At3g57290 (eIF3e; 40%)

MEESKQNYDLTPLIAPNLDRHLVFPIFEFLQERQLYPDEQILKSKIQLLNQTNMVDYAMDIHKSLYHTEDAPQEMVERR
TEVVARLKSLEEAAAPLVSFLLNPNAVQELRADKQYNLQMLKERYQIGPDQIEALYQYAKFQFECGNYSGAADYLYQYR
TLCSNLERSLSALWGKLASEILMQNWDIALEELNRLKEIIDSKSFSSPLNQVQNRIWLMHWGLYIFFNHDNGRTQIIDL
FNQDKYLNAIQTSAPHLLRYLATAFIVNKRRRPQLKEFIKVIQQEHYSYKDPIIEFLACVFVNYDFDGAQKKMKECEEV
IVNDPFLGKRVEDGNFSTVPLRDEFLENARLFVFETYCKIHQRIDMGVLAEKLNLNYEEAERWIVNLIRTSKLDAKIDS
ESGTVIMEPTQPNVHEQLINHTKGLSGRTYKLVNQLLEHTQAQATR

At1g64790 (ILA; 4%)

MSYSMVNASSAVSSPETAKNSDEPPPISSEAVNVLFPSVDPNSKLFRNSLNITISREAPPLTTSRIDFLSLFIFCKLTH
WLSLNPSSHRDEEEEEASPFYPFTIVLTYQPGPGQSPWKEMASPLESLLSISGSVSTSSTLIRLRIFRHDIPEILQNSD
MTSDIAPVIVDMIFQTLAIYDDRASRKAVDDLIVKGLGNVTFMKTFAAMLVQVMEKQLKFCFDTVCYRLLIWSCLLLEK
SQFATVSKNAFVRVASTQASLLRIIMESSFRMRRACKRFMFHLFSQSQAIYSLYMDEVKGSRIPYKDSPELLGLLLEFS
CSSPALFEQSKAIFVDIYVKDVLNSREKQKPNLSNCFKPLLQRLSHEEFQTVILPAAVKMLKRNPEIVLESVGFLLANV
NIDLSKYALELLPVILPQARHTDEDRRLGALSMVMCLSEKSSNPDTIEAMFASVKAIIGGSEGRLQSPHQRIGMLNAVQ
ELASAPEGKYIGSLSRTICSFLIACYKDEGNEDVKLSILSAVASWASRSSVAIQPNLVSFIAAGLKEKEALRRGHLRCV
RIICRNPDTISQISDLLSPLIQLVKTGFTKAVQRLDGIYALLIVSKIAACDIKAEDTMVKEKLWTLISQNEPSLVQITL
ASKLSSDDCVVCVDLLEVLLVEHSSRVLEAFSLKSLSQLLLFLLCHPSWNVRKTAYNSVTKIFLATSQLATTLLDEFSD
FLSITGDQIVSSRTSDADNPADHQAPFVPSVEVLVKALIVISSAAVAGPPSSWIVRAIFCSHHPSIVGTGKRDAVWKRL
QKCLKTCGFDVATFLSTNGESVCKSLLGPMGLTSAKTPEQQAAVYSLSTMMSLAPEDTFTVFKMHLQDLPDRLSHDMLS
ETDIKIFHTPEGMLLSEQGVYVAQTIGAKYTKQEPSSNHSLKKGLASRETANSGRRDTAKLTKKADKGKTAKEEARELM
LKEEASTRENVHRIQKSLSLVLHALGEMGLANPVFCHSQLPFLATFLDPLLRSPIVSAAAFENLVKLARCTVQPLCNWA
LEISTALRLIAIDEVDTSFDFRPSVDKAGKTYEGLFERIVNGLSISCKSGPLPVDTFTFIFPVLYHVLGVVPAYQASVG
PALNELCLGLQADDVANALYGVYSKDVHVRLACLNAVKCIPAVSKCSLPQNVKIATNIWIALHDPEKSVAESADDLWAR
YGHDLGTDYSGIFKALSHINLNVRLAAAEALADALHESPSSIQLSLSTLFSLYIRDATSGEDVFDAGWIGRQGIALALQ
SAADVLTTKDLPAVMTFLISRALADPNTDVRGKMINAGIMIIDKHGKENVSLLFPIFENYLNKEASDEEEYDLVREGVV
IFTGALAKHLARDDPKVHNVVEKLLEVLNTPSESVQRAVSTCLSPLVLSKQEEAPALFLRLLDKLMKSDKYGERRGAAF
GLAGVVMGFGISSLKKYGLIVTLQEALIDRNSAKRREGALLAFECLCEKLGKLFEPYVIKMLPLLLVSFSDQVGAVREA
AECAARAMMSQLSAYGVKLVLPSLLKGLEDKAWRTKQSSVQLLGAMAFCAPQQLSQCLPRVVPKLTEVFKTIQVLTDTH
PKVQSAGQLALQQVGSVIKNPEISSLVPTLLLALTDPNEYTRHALDTLLQTTFVNSVDAPSLALLVPIVHRGLRERSSE
TKKKASQIVGNMCSLVTEPKDMIPYIGLLLPEVKKVLVDPIPEVRSVAARAVGSLIRGMGEDNFPDLVPWLFETLKSDT
SNVERYGAAQGLSEVIAALGTDYFENILPDLIRHCSHQKASVRDGYLTLFKFLPRSLGAQFQKYLQLVLPAILDGLADE
NESVRDAALGAGHVLVEHHATTSLPLLLPAVEDGIFNDNWRIRQSSVELLGDLLFKVAGTSGKALLEGGSDDEGASTEA
QGRAIIDILGMDKRNEVLAALYMVRTDVSLSVRQAALHVWKTIVANTPKTLKEIMPILMSTLISSLASPSSERRQVAGR
SLGELVRKLGERVLPLIIPILSKGLKDPDVDKRQGVCIGLNEVMASAGRSQLLSFMDQLIPTIRTALCDSALEVRESAG
LAFSTLYKSAGLQAMDEIIPTLLEALEDDEMSTTALDGLKQIISVRTAAVLPHILPKLVHLPLSALNAHALGALAEVAG
AGFNTHLGTILPALLSAMGGENKEVQELAQEAAERVVLVIDEEGVETLLSELLKGVSDSQASIRRSSAYLIGYFFKSSK
LYLIDEAPNMISTLIVMLSDSDSTTVAVSWEALARVIGSVPKEVLPSYIKLVRDAVSTARDKERRKRKGGYVVIPGLCL
PKSLKPLLPVFLQGLISGSAELREQAAIGLGELIEVTSEQALKEFVIPITGPLIRIIGDRFPWQVKSAILATLIIILIQR
GGMALKPFLPQLQTTFVKCLQDSTRTIRSSAAVALGKLSALSTRIDPLVGDLMTSFQAADSGVREAILSAMRGVIKHAG
KSIGPAVRVRIFDLLKDLMHHEDDQVRISATSMLGVLSQYLEAAQLSVLLQEVNDLSASQNWGARHGSVLCISSLLKHN
PSTIMTSSLFSSMLNSLKSSLKDEKFPLRESSTKALGRLLLKQLATDPSNTKVVIDVLSSIVSALHDDSSEVRRRALSS
LKAFAKDNPSATMANISVIGPPLAECLKDGNTPVRLAAERCALHVFQLTKGAENVQAAQKYITGLDARRLSKFPEQSDD
SESDDDNVSG

At2g44060 (LEA26; 23%)

MSTSEDKPEIISRVVHQEGDVEIVDRSQKDKDEEKEEGKGGFLDKVKDFIHDIGEKLEGTIGFGKPTADVSAIHIPKIN
LERADIVVDVLVKNPNPVPIPLIDVNYLVESDGRKLVSGLIPDAGTLKAHGEETVKIPLTLIYDDIKSTYNDINPGMII
PYRIKVDLIVDVPVLGRLTLPLEKCGEIPIPKKPDVDIEKIKFQKFSLEETVAILHVRLQNMNDFDLGLNDLDCEVWLC
DVSIGKAEIADSIKLDKNGSGLINVPMTFRPKDFGSALWDMIRGKGTGYTIKGNIDVDTPFGAMKLPIIKEGGETRLKK
EDDDDDDEE

At4g20980 (eIF3d; 13%)

MVTEAFEFVAVPFNSDGWGPPDASDVSSSASPTSVAAANLLPNVPFASFSRSDKLGRVADWTR**NLSNPSARPNTGSK**SD
PSAVFDFSAFAIDEGFGLASSGGNPDEDAAFRLVDGKPPPRPKFGPKWRFNPHHNRNQLPQRRDEEVEAKKRDAEKERA
RRDRLYNNNRNNIHHQRREAAAFKSSVDIQPEWNMLEQIPFSTFSKLSYTVQEPEDLLLCGGLEYYNRLFDRITPKNER
RLERFKNRNFFK**VTTSDDPVIR**RLAKEDKATVFATDAILAALMCAPR**SVYSWDIVIQR**VGNKLFFDKRDGSQLDLLSVH
ETSQEPLPESKDDINSAHSLGVEAAYINQNFSQQVLVRDGKKETFDEANPFANEGEEIASVAYRYRRWKLDDNMHLVAR
CELQSVADLNNQR**SFLTLNALNEFDPK**YSGVDWRQKLETQRGAVLATELKNNGNKLAKWTAQALLANADMMKIGFVSRV
HPR**DHFNHVILSVLGYKPK**DFAGQINLNTSNMWGIVKSIVDLCMKLSEGK**YVLVKDPSKPQVR**IYEVPPDAFENDYVEE
PLPEDEQVQPTEENTEGAEASVAATKETEEKKADDAQA

At5g44320 (eIF3d; 9%)

MVFEAFEVGTVPFNSDGWGPPDASDTSSTSVAAANLLPNVPFASFSRSEKLGRVADWTRALSNPSARPHTGSKSDPSAI
FDFSAFAVDEGFGLTNSGGNADEDAAFRLVDGKPPPRPKFGPKWRFNQYHNRNQLPQRRDEEVEAKKREAEKDRARRDR
LYNNNRNNIHQQRREAAAFKSSVDIQPEWNMLEQIPFSTFSKLSFTVSEPEDLLLCGGLESYDRSFDRITPKADRRLER
FKNRSFKVTTSDDLVIRRLAKEDKATVFATDAILAALMCAPR**SVYSWDLVIQR**VGNKLFFDKRDGSPLDLLSVHETSQE
PLPEGKDDINSAHSLGLEAAYINQNFAQQVLVKNGKRETFDEPIPNVNEGEENASIAYRYRRWKLDDSMYLVARCELQS
TVDLNNQR**SFLTLNALNEFDPK**YSGVDWRQKLETQRGAVLANELKNNGNKLAKWTAQALLANADMMKIGFVSRVHPR**DH**
FNHVILSVLGYKPKDFAGQINLNTNNMWGIVKSIVDLCMKLSEGK**YVLVKDPSKPQVR**IYEVPADAFDNDYVEEPLPED
EQVQPPEENTDAGAETNGVSSTNVAVEDKKSEVEA

At5g17020 (XPO1A; 6%)

MAAEKLRDLSQPIDVGVLDATVAAFFVTGSKEERAAADQILRDLQANPDMWLQVVHILQNTNSLDTK**FFALQVLEGVIK**
YRWNALPVEQRDGMKNYISEVIVQLSSNEASFRSERLYVNK**LNVILVQIVK**HDWPAKWTSFIPDLVAAAKTSETICENC
MAILK**LLSEEVFDFSR**GEMTQQKIKELKQSLNSEFKLIHELCLYVLSASQRQDLIRATLSALHAYLSWIPLGYIFESTL
LETLLKFFPVPAYRNLTIQCLTEVAALNFGDFYNVQYVKMYTIFIGQLRIILPPSTKIPEAYSSGSGEEQAFIQNLALF
FTSFFKFHIRVLESTPEVVSLLLAGLEYLINISYVDDTEVFKVCLDYWNSLVLELFDAHHNSDNPAVSASLMGLQPFLP
GMVDGLGSQVMQRRQLYSHPMSKLRGLMINRMAKPEEVLIVEDENGNIVRETMKDNDVLVQYKIMRETLIYLSHLDHDD
TEKQMLRKLNKQLSGEEWAWNNLNTLCWAIGSISGSMAEDQENRFLVMVIRDLLNLCEITKGKDNKAVIASNIMYVVGQ
YPRFLRAHWKFLKTVVNKLFEFMHETHPGVQDMACDTFLKIVQKCKRKFVIVQVGENEPFVSELLTGLATTVQDLEPHQ
IHSFYESVGNMIQAESDPQKRDEYLQRLMALPNQKWAEIIGQARHSVEFLKDQVVIRTVLNILQTNTSAATSLGTYFLS
QISLIFLDMLNVYRMYSELVSTNITEGGPYASKTSFVKLLRSVKRETLK**LIETFLDKAEDQPHIGK**QFVPPMMESVLGD
YARNVPDAR**ESEVLSLFATIINK**YKATMLDDVPHIFEAVFQCTLEMITKNFEDYPEHRLKFFSLLRAIATFCFPALIKL
SSPQLKLVMDSIIWAFRHTERNIAETGLNLLLEMLKNFQQSEFCNQFYRSYFMQIEQEIFAVLTDTFHKPGFKLHVLVL
QQLFCLPESGALTEPLWDATTVPYPYPDNVAFVREYTIKLLSSSFPNMTAAEVTQFVNGLYESRNDPSGFKNNIRDFLV
QSKEFSAQDNKDLYAEEAAAQREREROQRMLSIPGLIAPNEIQDEMVDS

At3g03110 (XPO1B; 2%)

MAAEKLRDLSQPIDVVLLDATVEAFYSTGSKEERASADNILRDLKANPDTWLQVVHILQNTSSTHTK**FFALQVLEGVIK**
YRWNALPVEQRDGMKNYISDVIVQLSRDEASFRTERLYVNKLNIILVQIVKQEWPAKWKSFIPDLVIAAKTSETICENC
MAILKLLSEEVFDFSKGEMTQQKIKELKQSLNSEFQLIHELCLYVLSASQRQELIRATLSALHAYLSWIPLGYIFESPL
LEILLKFFPVPAYRNLTLQCLSEVASLNFGDFYDMQYVKMYSIFMNQLQAILPLNLNIPEAYSTGSSEEQAFIQNLALF
FTSFFKLHIKILESAPENISLLLAGLGYLISISYVDDTEVFKVCLDYWNSLVLELFGTRHHACHPALTPSLFGLQMAFL
PSTVDGVKSEVTERQKLYSDPMSKLRGLMISRTAKPEEVLIVEDENGNIVRETMKDNDVLVQYKIMRETLIYLSHLDHE
DTEKQMLSKLSKQLSGEEWAWNNLNTLCWAIGSISGSMVVEQENRFLVMVIRDLLSLCEVVKGKDNKAVIASNIMYVVG
QYSRFLRAHWKFLKTVVHKLFEFMHETHPGVQDMACDTFLKIVQKCKRKFVIVQVGESEPFVSELLSGLATIVGDLQPH
QIHTFYESVGSMIQAESDPQKRGEYLQRLMALPNQKWAEIIGQARQSADILKEPDVIRTVLNILQTNTRVATSLGTFFL
SQISLIFLDMLNVYRMYSELVSSSIANGGPYASRTSLVKLLRSVKREILKLIETFLDKAENQPHIGKQFVPPMMDQVLG
DYARNVPDAR**ESEVLSLFATIINK**YKVVMRDEVPLIFEAVFQCTLEMITKNFEDYPEHRLKFFSLLRAIATFCFRALIQ
LSSEQLKLVMDSVIWAFRHTERNIAETGLNLLLEMLKNFQKSDFCNKFYQTYFLQIEQEVFAVLTDTFHKPGFKLHVLV
LQHLFSLVESGSLAEPLWDAATVPHPYSNNVAFVLEYTTKLLSSSFPNMTTTEVTQFVNGLYESRNDVGRFKDNIRDFL
IQSKEFSAQDNKDLYAEEAAAQMERERQRMLSIPGLIAPSEIQDDMADS

At1g61580 (RPL3B; 12%)

MSHRKFEHPRHGSLGFLPRKRASRHRGKVKAFPKDDPTKPCRLTSFLGYKAGMTHIVRDVEKPGSKLHKKETCEAVTII
ETPPMVVVGVVGYVKTPRGLRSLCTVWAQHLSEELRRRFYKNWAKSKKKAFTRYSKKHETEEGKKDIQSQLEKMKKYCS
VIRVLAHTQIRKMKGLKQKKAHLNEIQINGGDIAKKVDYACSLFEKQVPVDAIFQKDEMIDIIGVTKGKGYEGVVTRWG
VTRLPRKTHRGLRKVACIGAWHPARVSYTVARAGQNGYHHRTEMNKKVYRVGKVGQETHSAMTEYDRTEKDITPMGGFP
HYGIVKEDYLMIKGCCVGPKKRVVTLRQTLLKQTSRLAMEEIKLKFIDAASNGGHGRFQTSQEKAKFYGRTIKA

At4g38740 (ROC1; 38%)

MAFPKVYFDMTIDGQPAGRIVMELYTDKTPRTAENFRALCTGEKGVGGTGKPLHFKGSKFHRVIPNFMCQGGDFTAGNG
TGGESIYGSKFEDENFERKHTGPGILSMANAGANTNGSQFFICTVKTDWLDGKHVVFGQVVEGLDVVKAIEKVGSSSGK
PTKPVVVADCGQLS

At3g13460 (ECT2; 11%)

MATVAPPADQATDLLQKLSLDSPAKASEIPEPNKKTAVYQYGGVDVHGQVPSYDRSLTPMLPSDAADPSVCYVPNPYNP
YQYYNVYGSGQEWTDYPAYTNPEGVDMNSGIYGENGTVVYPQGYGYAAYPYSPATSPAPQLGGEGQLYGAQQYQYPNYF
PNSGPYASSVATPTQPDLSANKPAGVKTLPADSNNVASAAGITKGSNGSAPVKPTNQATLNTSSNLYGMGAPGGGLAAG
YQDPRYAYEGYYAPVPWHDGSKYSDVQRPVSGSGVASSYSKSSTVPSSRNQNYRSNSHYTSVHQPSSVTGYGTAQGYYN
RMYQNKLYGQYGSTGRSALGYGSSGYDSRTNGRGWAATDNKYRSWGRGNSYYYGNENNVDGLNELNRGPRAKGTKNQKG
NLDDSLEVKEQTGESNVTEVGEADNTCVVPDREQYNKEDFPVDYANAMFFIIKSYSEDDVHKSIKYNVWASTPNGNKKL
AAAYQEAQQKAGGCPIFLFFSVNASGQFVGLAEMTGPVDFNTNVEYWQQDKWTGSFPLKWHIVKDVPNSLLKHITLENN
ENKPVTNSRDTQEVKLEQGLKIVKIFKEHSSKTCILDDFSFYEVRQKTILEKKAKQTQKQVSEEKVTDEKKESATAESA
SKESPAAVQTSSDVKVAENGSVAKPVTGDVVANGC

At4g33250 (eIF3k; 23%)

MGVEIQSPQEQSSYTVEQLVALNPFNPEILPDLENYVNVTSQTYSLEVNLCLLRLYQFEPERMNTHIVARILVKALMAM
PTPDFSLCLFLIPERVQMEEQFKSLIVLSHYLETGRFQQFWDEAAKNRHILEAVPGFEQAIQAYASHLLSLSYQKVPRS
VLAEAVNMDGASLDKFIEQQVTNSGWIVEKEGGSIVLPQNEFNHPELKKNTGENVPLEHIARIFPILG

At1g76810 (eIF5B; 4%)

MGRKKPSARGGDAEQQPPASSLVGATKSKKKGAQIDDDEYSIGTELSEESKVEEEKVVVITGKKKGKKGNKKGTQQDDD
DDFSDKVSAAGVKDDVPEIAFVGKKKSKGKKGGGSVSFALLDDEDEKEDNESDGDKDDEPVISFTGKKHASKKGKKGGN
SFAASAFDALGSDDDDTEEVHEDEEEESPITFSGKKKKSSKSSKKNTNSFTADLLDEEEGTDASNSRDDENTIEDEESP
EVTFSGKKKSSKKKGGSVLASVGDDSVADETKTSDTKNVEVVETGKSKKKKKNNKSGRTVQEEEDLDKLLAALGETPAA
ERPASSTPVEEKAAQPEPVAPVENAGEKEGEEETAAAKKKKKKKEKEKEKKAAAAAAATSSVEVKEEKQEESVTEPLQP
KKKDAKGKAAEKKIPKHVREMQEALARRQEAEERKKKEEEEKLRKEEEERRRQEELEAQAEEAKRKRKEKEKEKEKLLRKK
LEGKLLTAKQKTEAQKREAFKNQLLAAGGGLPVADNDGDATSSKRPIYANKKKSSRQKGIDTSVQGEDEVEPKENQADE
QDTLGEVGLTDTGKVDLIELVNTDENSGPADVAQENGVEEDDEEDEWDAKSWGTVDLNLKGDFDDEEEEAQPVVKKELK
DAISKAHDSEPEAEKPTAKPAGTGKPLIAAVKATPEVEDATRTKRATRAKDASKKGKGLAPSESIEGEENLRSPICCIM
GHVDTGKTKLLDCIRGTNVQEGEAGGITQQIGATYFPAENIRERTKELKADAKLKVPGLLVIDTPGHESFTNLRSRGSS
LCDLAILVVDIMHGLEPQTIESLNLLRMRNTEFIVALNKVDRLYGWKTCKNAPIVKAMKQQNKDVINEFNLRLKNIINE
FQEQGLNTELYYKNKDMGDTFSIVPTSAISGEGVPDLLLWLVQWAQKTMVEKLTYVDEVQCTVLEVKVIEGHGTTIDVV
LVNGELHEGDQIVVCGLQGPIVTTIRALLTPHPMKELRVKGTYLHYKEIKAAQGIKITAQGLEHAIAGTALHVVGPDDD
IEAIKESAMEDMESVLSRIDKSGEGVYVQASTLGSLEALLEYLKSPAVKIPVSGIGIGPVHKKDVMKAGVMLERKKEYA
TILAFDVKVTTEARELADEMGVKIFCADIIYHLFDLFKAYIENIKEEKKKESADEAVFPCVLQILPNCVFNKKDPIVLG
VDVIEGILKIGTPICVPGREFIDIGRIASIENNHKPVDYAKKGNKVAIKIVGSNAEEQKMFGRHFDMEDELVSHISRRS
IDILKSNYRDELSLEEWKLVVKLKNIFKIQ

At3g53610 (RAB8; 18%)

MAAPPARARADYDYLIKLLLIGDSGVGKSCLLLRFSDGSFTTSFITTIGIDFKIRTIELDGKRIKLQIWDTAGQERFRT
ITTAYYRGAMGILLVYDVTDESSFNNIRNWIRNIEQHASDSVNKILVGNKADMDESKRAVPKSKGQALADEYGMKFFET
SAKTNLNVEEVFFSIAKDIKQRLADTDARAEPQTIKINQSDQGAGTSQATQKSACCGT

At5g37475 (eIF3j; 16%)

MDDWEAEDFQPLPSKVELKSNWDDEDVDENDIKDSWEEEDVSAPPPIVKPASEKAPKKPAVKAVEKKVKTVEAPKGTSR
EEPLDPIAEKLRMQRLVEEADYQSTAELFGVKTEEKSVDMLIPKSESDFLDYAELISQRLVPFEKSFHYIGLLKAVMRL
SVANMKAADVKDVASSITAIANEKLKAEKEAAAGKKKSGKKKQLHVDKPDDDLVSGPYDAMDDDDFM

At3g43600 (AAO2; 3%)

MSLVFAINGQRFELELSSVDPSTTLLEFLRYQTSFKSVKLSCGEGGCGACVVLLSKFDPVLQKVEDFTVSSCLTLLCSV
NHCNITTSEGLGNSRDGFHPIHKRLSGFHASQCGFCTPGMSVSLFSALLDADKSQYSDLTVVEAEKAVSGNLCRCTGYR
PIVDACKSFASDVDIEDLGLNSFCRKGDKDSSSLTRFDSEKRICTFPEFLKDEIKSVDSGMYRWCSPASVEELSSLLEA
CKANSNTVSMKLVAGNTSMGYYKDEREQNYDKYIDITRIPHLKEIRENQNGVEIGSVVTISKVIAALKEIRVSPGVEKI
FGKLATHMEMIAARFIRNFGSIGGNLVMAQRKQFPSDMATILLAAGAFVNIMSSSRGLEKLTLEEFLERSPLEAHDLVL
SIEIPFWHSETNSELFFETYRAAPRPHGSALAYLNAAFLAEVKDTMVVNCRLAFGAYGTKHAIRCKEIEEFLSGKVITD
KVLYEAITLLGNVVVPEDGTSNPAYRSSLAPGFLFKFLHTLMTHPTTDKPSNGYHLDPPKPLPMLSSSQNVPINNEYNP
VGQPVTKVGASLQASGEAVYVDDIPSPTNCLYGAFIYSKKPFARIKGIHFKDDLVPTGVVAVISRKDVPKGGKNIGMKI
GLGSDQLFAEDFTTSVGECIAFVVADTQRHADAAVNLAVVEYETEDLEPPILSVEDAVKKSSLFDIIPFLYPQQVGDTS
KGMAEADHQILSSEIRLGSQYVFYMETQTALAVGDEDNCIVVYSSTQTPQYVQSSVAACLGIPENNIRVITRRVGGGFG
GKSVKSMPVATACALAAKKLQRPVRTYVNRKTDMIMTGGRHPMKITYSVGFKSTGKITALELEILIDAGASYGFSMFIP
SNLIGSLKKYNWGALSFDIKLCKTNLLSRAIMRSPGDVQGTYIAEAIIENIASSLSLEVDTIRKINLHTHESLALFYKD
GAGEPHEYTLSSMWDKVGVSSKFEERVSVVREFNESNMWRKRGISRVPIIYEVLLFATPGRVSVLSDGTIVVEIGGIEL
GQGLWTKVKQMTSYALGMLQCDGTEELLEKIRVIQSDSLSMVQGNFTGGSTTSEGSCAAVRLCCETLVERLKPLMERSD
GPITWNELISQAYAQSVNLSASDLYTPKDTPMQYLNYGTAVSEVEVDLVTGQTTVLQTDILYDCGKSLNPAVDLGQIEG
SFVQGLGFFMLEEYIEDPEGLLLTDSTWTYKIPTVDTIPKQFNVEILNGGCHEKRVLSSKASGEPPLLLAASVHCATRQ
AVKEARKQLCMWKGENGSSGSAFQLPVPATMPVVKELCGLDIIESYLEWKLHDNSNL

At1g65860 (FMO GS-OX1; 4%)

MAPTQNTICSKHVAVIGAGAAGLVTARELRREGHTVVVFDREKQVGGLWNYSSKADSDPLSLDTTRTIVHTSIYESLRT
NLPRECMGFTDFPFVPRIHDISRDSRRYPSHREVLAYLQDFAREFKIEEMVRFETEVVCVEPVNGKWSVRSKNSVGFAA
HEIFDAVVVCSGHFTEPNVAHIPGIKSWPGKQIHSHNYRVPGPFNNEVVVVIGNYASGADISRDIAKVAKEVHIASRAS
ESDTYQKLPVPQNNLWVHSEIDFAHQDGSILFKNGKVVYADTIVHCTGYKYYFPFLETNGYININENRVEPLYKHVFLP
ALAPSLSFIGLPGMAIQFVMFEIQSKWVAAVLSGRVILPSQDKMMEDIIEWYATLDVLGIPKRHTHKLGKISCEYLNWI
AEECHCSPVENWRIQEVERGFQRMVSHPEIYRDEWDDDDLMEEAYKDFARKKLISSHPSYFLES

At2g20830 (Folic acid binding / transferase; 8%)

MSSGLNEDFLDCIVRLEETHVQQGFDEGYEEGLVSGREDARHLGLKLGFETGELIGFYRGCSALWNSALRIDPTRFSPQ
LHKHLNDFHVLLDKIPLLDPEDEAKDGIKDDLRVKFSIICASLGFSKKQFEWSEEMLREMLGCCKVYISEARNKTALEA
IERALKPFPPAAIVNKFEDAAYGRVGYTVVSSLANGSSSSLKNAVFAMVKTALDTINLELHCGSHPRLGVVDHICFHPL
SQTSIEQVSSVANSLAMDIGSILRVPTYLYGAAEKEQCTLDSIRRKLGYFKANREGHEWAGGFDLEMVPLKPDAGPQEV
SKAKGVVAVGACGWVSNYNVPVMSNDLKAVRRIARKTSERGGGLASVQTMALVHGEGVIEVACNLLNPSQVGGDEVQGL
IERLGREEGLLVGKGYYTDYTPDQIVERYMDLLNNS

At5g58410 (HEAT/U-box domain-containing protein; 1%)

MGKPKGDAARSKARPSSSSLAASLLPSGSAAAVGFGGYVGSSRFQTSLSNEDSASFLDLDSEVAQHLQRLSRKDPTTKI
KALASLSELVKQKQGKELLPIIPQWTFEYKKLILDYSRDVRRATHDVMTNVVTGAGRDIAPHLKSIMGPWWFSQFDLAS
EVSQAAKSSFQVGSSFGNSVFLVEAAFPAQEKRLHALNLCSAEIFAYLEENLKLTPQNLSDKSLASDELEEMYQQMISS
SLVGLATLLDILLREPDNTGSANINSESKLASKARAVATSSAEKMFSSHKCFLNFLKSESPSIRSATYSLLSSFIKNVP
EVFGEGDVRSLAPALLGVFRENNPTCHSSMWEAVLLFSKKFPQSWVYLNVHKSVLNHLWQFLRNGCYGSPQVSYPALIL
FLEVMPAQSVESDKFFVNFFKNLLAGRSMCESSSTDQLSLLRATTECFLWGLRNASRYCDVPNSIHDLQVDLIDKVLVK
ILWADFTELSKGSIPPNQRKSAENLGMGNSVSYLQELGRCILEILSGINLLEQNLLSFFCKAVQESFLNMLQQGDLEIV
AGSMRKMIDFLLLLERYSVLEGESWPLHQFMGPLLSKAFPWIRSSELLDGVKLLSVSVSVFGPRKVVPVLIDDIETSTL
LSVEKEKNMSPEKLIKVFQEIFIPWCMDGYDSSTAARQDLLFSLLDDECFTQQWSDVISYVFNQQHQGFNNLAAMKMLL
EKARDEITKRSSGQELNQRIGSRPEHWHHTLIESTAISLVHSSSATTSAVQFLCSVLGGSTQDSSISFVSRSSLVLIY
RGILEKLLSFIKQSPLCSVNDTCSSLIVEAIAFDSSSSVDVIVVAKFAAEVIDGSFFSLKSLSQDATLLTTVLSSIFII
DLENRMTSLVDNTLSESKEKRKDRNFVCDYVHAVCSKMDNQFWKSINYDVRKSSASTLAQFLRSVVLLEDDLQPFELTL
LCASRMTEVLEYLSLDQSDEENICGLLLLESDAWPIWVSPSSSASIDTHGMPVQLCELRKSKSQRYVSFIDSLIMKLGI
HRFIVGHKDHGFASQAWLSVEILCTWEWPGGKVQTSFLPNLVSFCKDEPSSGGLLNSIFDILLNGALVHVKDEEEGLGN
MWVDFNNNIVDVVEPFLRALVSFLHILFKEDLWGEEEAMAAFKMITDKLFIGEETSKNCLRIIPYIMSIIISPLRTKVK
SGGSGKDTLLPLEVLLRNWLERSLSFPPLVLWQSGEDIQDWFQLVISCYPVSDKAEEAKELQRHLSTEERTLLLDLFRK
QKQDPGASTVVTQLPAVQILLARLIMIAVSYCGNDFNEDDWDFVFSNLKRLIQSAVVVMEETSENVNDFISGVSSMEKE
KENDTLEGLGHIVFISDPSINSAQNALSAFSLLNALVNHKSVEGEDNLKSLADETWDPVKDRILEGVLRLFFCTGLTEA
IAASYSPEAASIVASFRVDHLQFWELVAHLVVDSSPRARDRAVRAVEFWGLSRGSISSLYAIMFSSNPIPSLQLAAYTV
LSTEPISRLAIVADLNAPLNDESLNDQDSSNAGLPSEDKLLLRDEVSCMVEKLDHELLDTDLTAPERVQTFLAWSLLLS
NVNSLPSLTQGRERLVQYIEKTANPLILDSLFQHIPLELYMGQSLKKKDGDIPSELSVVASAATRAIITGSSLSTVESL
WPIETGKMASLAGAIYGLMLRVLPAYVREWFSEMRDRSASSLIEAFTRTWCSPSLIKNELSQIKKADFNDESFSVSISK
AANEVVATYTKDETGMDLVIRLPVSYPLKPVDVNCAKSIGISEAKQRKWLMSMQMFVRHQNGALAEAIRIWKRNSDKEF
EGVEDCPICYSVIHIGNHSLPRRACVTCKYKFHKACLDKWFYTSNKKLCPLCQSPC

At2g42910 (PRS4; 8%)

MSENAANNIMETKICTDAIVSELQKKKVHLFYCLECEELARNIAAESDHITLQSINWRSFADGFPNLFINNAHDIRGQH
VAFLASFSSPAVIFEQISVIYLLPRLFVASFTLVLPFFPTGSFERMEEEGDVATAFTMARIVSNIPISRGGPTSVVIYD
IHALQERFYFADQVLPLFETGIPLLTKRLQQLPETEKVIVAFPDDGAWKRFHKLLDHYPTVVCTKVREGDKRIVRLKEG
NPAGCHVVIVDDLVQSGGTLIECQKVLAAHGAVKVSAYVTHGVFPKSSWERFTHKKNGLEEAFAYFWITDSCPQTVKAI
GNKAPFEVLSLAGSIADALQI

At3g08850 (RAPTOR1; 1%)

MALGDLMVSRFSQSSVSLVSNHRYDEDCVSSHDDGDSRRKDSEAKSSSSYGNGTTEGAATATSMAYLPQTIVLCELRHD
ASEASAPLGTSEIVLVPKWRLKERMKTGCVALVLCLNITVDPPDVIKISPCARIEAWIDPFSMAPPKALETIGKNLSTQ
YERWQPRARYKVQLDPTVDEVRKLCLTCRKYAKTERVLFHYNGHGVPKPTANGEIWVFNKSYTQYIPLPISELDSWLKT
PSIYVFDCSAARMILNAFAELHDWGSSGSSGSSRDCILLAACDVHETLPQSVEFPADVFTSCLTTPIKMALKWFCRRSL
LKEIIDESLIDRIPGRQNDRKTLLGELNWIFTAVTDTIAWNVLPHELFQRLFRQDLLVASLFRNFLLAERIMRSANCNP
ISHPMLPPTHQHHMWDAWDMAAEICLSQLPQLVLDPSTEFQPSPFFTEQLTAFEVWLDHGSEHKKPPEQLPIVLQVLLS
QCHRFRALVLLGRFLDMGSWAVDLALSVGIFPYVLKLLQTTTNELRQILVFIWTKILALDKSCQIDLVKDGGHTYFIRF
LDSSGAFPEQRAMAAFVLAVIVDGHRRGQEACLEANLIGVCLGHLEASRPSDPQPEPLFLQWLCLCLGKLWEDFMEAQI
MGREANAFEKLAPLLSEPQPEVRAAAVFALGTLLDIGFDSNKSVVEDEFDDDEKIRAEDAIIKSLLDVVSDGSPLVRAE
VAVALARFAFGHKQHLKLAAASYWKPQSSSLLTSLPSIAKFHDPGSATIVSLHMSPLTRASTDSQPVARESRISSSPLG
SSGLMQGSPLSDDSSLHSDSGMMHDSVSNGAVHQPRLLDNAVYSQCVRAMFALAKDPSPRIASLGRRVLSIIGIEQVVA
KPSKPTGRPGEAATTSHTPLAGLARSSSWFDMHAGNLPLSFRTPPVSPPRTNYLSGLRRVCSLEFRPHLLGSPDSGLAD
PLLGASGSERSLLPLSTIYGWSCGHFSKPLLGGADASQEIAAKREEKEKFALEHIAKCQHSSISKLNNNPIANWDTRFE
TGTKTALLHPFSPIVVAADENERIRVWNYEEATLLNGFDNHDFPDKGISKLCLINELDDSLLLVASCDGSVRIWKNYAT
KGKQKLVTGFSSIQGHKPGARDLNAVVDWQQQSGYLYASGETSTVTLWDLEKEQLVRSVPSESECGVTALSASQVHGGQ
LAAGFADGSLRLYDVRSPEPLVCATRPHQKVERVVGLSFQPGLDPAKVVSASQAGDIQFLDLRTTRDTYLTIDAHRGSL
TALAVHRHAPIIASGSAKQLIKVFSLQGEQLGIIRYYPSFMAQKIGSVSCLTFHPYQVLLAAGAADSFVSIYTHDNSQA
R

At5g01770 (RAPTOR2; 1%)

MALGDLMVSRLSQSSVTVVTNHLYDDDDNCASSAHDDSRVSIIASPRVASSSYENLSAATSMAYLPQTLVLCDLRHDDA
SDIVQPPRWRLKERMKTGCVALVMCLHITVDPPDVIKISPCARLECWIDPFSMFPPRRALEAIGQNLSIQYERWLARAR
YKVELDPTKDDVRKLCLSCRKYAKTERVLFHYNGHGVPKPTPNGEIWVYNKNFTQYIPLPVSELDSWLKTPTIYVFDCS
AARVILNAFAEGESSGPPKDCILLAACDVHETLPQSVEFPADVFTSCLTTPINIALKWFCRRSLLKEFIDESLIDRIPG
RQNDRKTLLGELNWIFTAVTDTIAWNVLPRELFQRLFRQDLLVASLFRNFLLAERIMRSGNCTPISHPMLPPTHQHHMW
DAWDMAAEICLSQLPQFFLDPNTEFQPSSFFTEQLTAFEVWLDHGSEHKKPPEQLPIVLQVLLSQCHRYRALVLLGRFL
DMGPWAVDLALSVGIYPCVVKLLQTTTIELRQILVFIWTKILALDKSCQVDLVKDRGHIYFIRFLDSSDAFPEQRAMAA
FILAVIVDGYKRGQESCLEANLIAVCLGHLEATQLCDPPPEPLFLQWLCLCLGKLWEDYLEAQIMGREANASENLIAGH
TNLLQVRAAAVFALGTLLDVGFDSGKGVCDEEFDDDENIVEDIIIKSLLDVVSDGSPLVRTEVAVALARFAFGHKQHLK
SVADSYWKPQNSLRTSLPSMAKFHDSGTSIVASSDMGSLTRASPDSQPVAREGRISSSLQEPFSGLMQGSPLADDSSLH
SDVGIIHDGVSNGVVHQPRPLDNAIYSQSVLAMFTLAKDPSPRIASLGRRVLSVIGIEQIVAKPSKSNGRPGEAASASH
TPLAGLVRSSSWFDMHTGHLPLTFRTPPVSPPQTSYLTGLRRVCSLELRPHLLGSPDSGLADPILGVSGSERSLLPQST
IYNWSCGHFSKPLLGGADANEEIAAQREEKKKFSLEHIAKCQHSSISGLSNIPIANWDTKFETGTKTALLHPFSPIVVA
ADENERIRVWNYEEATLLNGFDNNDFPDKGISNLCLVNELDDSLLLVASCNVPTLSRASFAIRIWKDYATKGRQKLVTG
FSSIQGQKPGASGLNAVVDWQQQSGYLYVSGESLSIMVWDLDKEQLVKSMPFESGCSVTALSASQVHGSQLAAGFADGS
VRLYDVRTPDFLVCATRPHQRVEKVVGLSFQPGLDPAKIVSASQAGDIQFLDLRRPKETYLTIDAHRGSLTALGVHRHA
PIIASGSAKQLIKVFSLKGEQLGIIKYHTSFMGQQIGPVSCLAFHPYQMLLAAGAAGSFVSLYTHHNTQLPR

Supplementary Figure S12. Amino acid sequences of proteins identified by LC-ESI-MS/MS in co-immunoprecipitated ABCE2:YFP protein. The Arabidopsis Genome Initiative (AGI) gene identifier (AtNgNNNNN) is shown, together with the TAIR10 annotation or protein name and peptide coverage (in percentage) for each protein. Full-length protein sequences were obtained from TAIR. The unique peptides identified by LC-ESI-MS/MS are shaded in black; some unique peptide sequences overlap. Peptide coverage was calculated by dividing the total number of residues of each protein by that of those covered by the peptides.

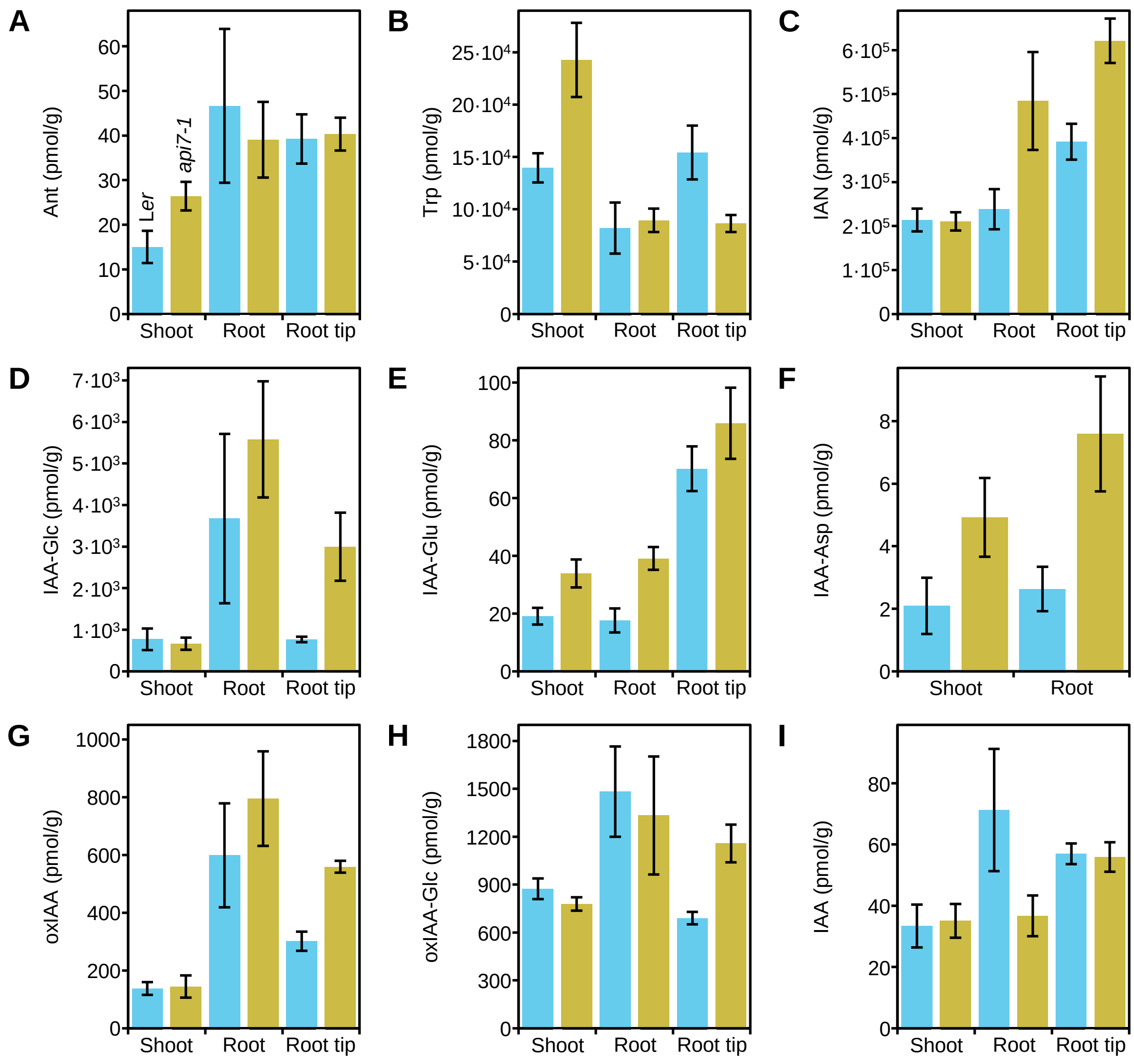

Supplementary Figure S13. Tissue profiling of IAA metabolites in *api7-1* seedlings. The levels of **(A–C)** IAA precursors, **(D)** the IAA storage molecule IAA-Glc, **(E–H)** IAA catabolites, and **(I)** IAA were quantified in shoots, roots, and root tips from L*er* and *api7-1* seedlings 9 das. Concentrations are shown as the mean values from four biological replicates in pmol·g^{-1} of fresh weight. Error bands represent the standard deviation.

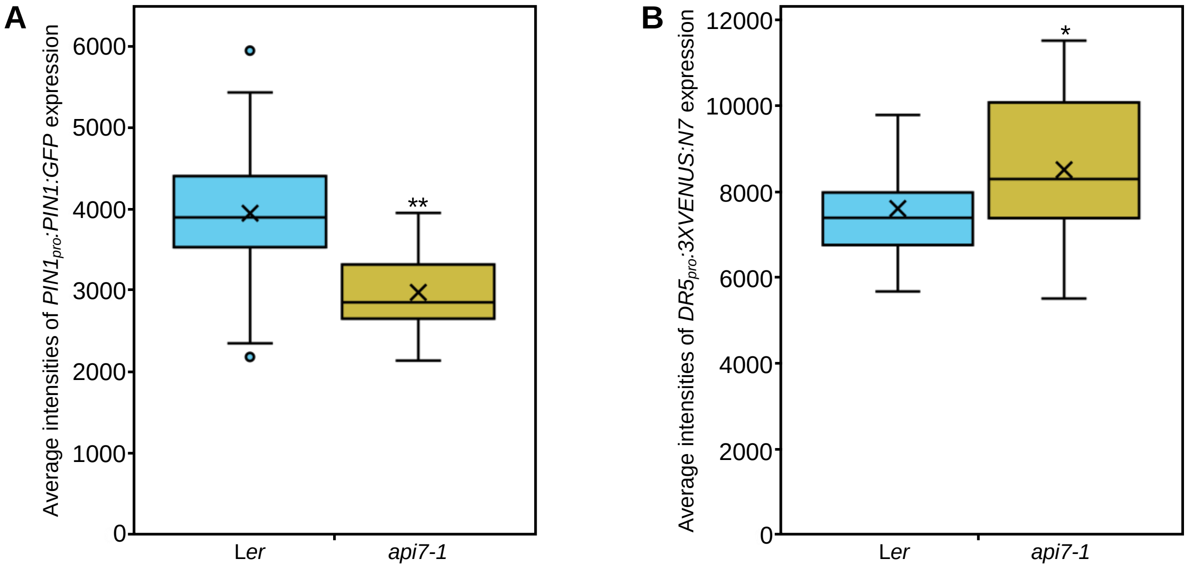

Supplementary Figure S14. Average fluorescence intensities of *PIN1$_{pro}$:PIN1:GFP* and *DR5$_{pro}$:3XVENUS:N7* expression in L*er* and *api7-1* root tips. Boxplot distributions of average fluorescence intensities of **(A)** GFP and **(B)** VENUS. Measurements were performed on pictures taken 5 das [**(A)** n = 25; **(B)** n = 27]. Other details as described in the legend of Supplementary Figure S1 for its (A) section. Asterisks indicate a significant difference with the wild-type in a Student's t test (*P < 0.05, **P < 0.001).

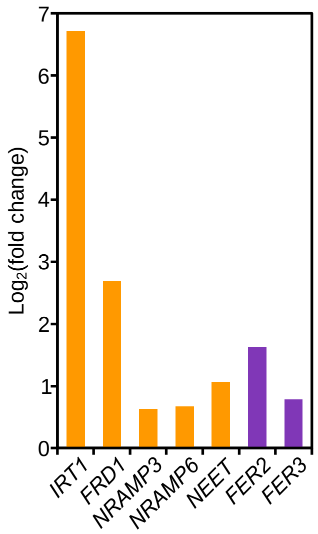

Supplementary Figure S15. Expression levels of some genes deregulated in *api7-1* plants. Expression levels of genes related to iron homeostasis and FeS cluster biogenesis (orange), and response to oxidative stress (purple). Values are shown as the binary logarithm of the foldchange between *api7-1* and L*er* mean reads. Mean reads were calculated from three biological replicates.

Supplementary Table S1. Primer sets used in this work

Purpose	Name	Forward primer (F; 5′ → 3′)	Reverse primer (R; 5′ → 3′)
Linkage analysis	nga1111_F/R	GGGTTCGGTTACAATCGTGT	AGTTCCAGATTGAGCTTTGAGC
	AtF28J12.3_F/R	GCTCCGCCGTTGGATTCTG	GTTCGGGTTTAATTCTCGGGT
	AtM7J12.1_F/R	AGCAACTTGTGTTCTCATTT	TTATAGGGTACGACAACCAT
	nga1139_F/R	CTAGGCTCGGGTGAGTCAC	TTTTTCCTTGTGTTGCATTCC
	nga1107_F/R	GCGAAAAAACAAAAAAATCCA	CGACGAATCGACAGAATTAGG
	g3883_F/R	CATCCATCAAACAAACTCC	TGTTTCAGAGTAGCCAATTC
	T13K14_F/R	CTGAAACATATAAGAGAATCATCC	ACTCGTAGTTTGGTGTTGAGAC
	AtF16G20.1_F/R	TCAGTGTTACTATGTACCAAGTA	TAGGACGTAATATCCTTAGTTAC
	AG_F/R	CAACAGGTTTCTTCTTCTTCTC	CAAACACCATTTAATCTTGACA
	T18B16_F/R	TAACTTCTTGCAGCCTCTGAAG	TTCTATTGGGATGCTGCCCTC
Sequencing of *ABCE1* and *ABCE2*	ABCE1_F1/R1	GGTTAGCTAGTCCCTTTCAAAG	GAAGTATGCTAATGTGGCCC
	ABCE1_F2/R2	ACTACCTCTTGCGCAGACTC	GGCATCAGACTCACTTCATGA
	ABCE1_F3/R3	GGAAGTGAAGTCCTATGCAAGA	CTTGAAATCTCCAAGTTGCTTAGT
	ABCE1_R4		CTGCCAAGATTTGGTTTGAG
	ABCE2_F1/R1	TCGGTTCACCATTTTTATCTGAAG	CACCACAAGATGCTAACAATGAT
	ABCE2_F2/R2	AGCCTGCGGATATATACCTGAT	GGTCGTATCTTTCTCCAAGTCT
	ABCE2_F3/R3	GTTACACCGTATGGGCAAGAG	GGAGACTTACAGATAAGAAGAGA
	ABCE2_F4/R4	CTTAGAACAATCGGCACACG	AGAGAAATCGAGATTAGTACCTGAG
	ABCE2_F5/R5	GTTCTGATACCCTGTGCATG	ATCTCTTCAGCACTTTCTTGTG
Genotyping	api7-1_F/R	TGCCTCTAGAAATGGCACCT	GTATGGCAACAACAGCGATT
	GABI_509C06_LP/RP	TTCTTGGTCTGAAATTGGTGG	TGGCTGGATTTGTTCCTACAG
	o8409 (GABI-Kat lines)[1]	ATATTGACCATCATACTCATTGC	
	M13_F/R	TGTAAAACGACGGCCAGT	GGAAACAGCTATGACCATGATT
	GFP_R		CACGTATCCCTCAGGCATGG
	YFP_R		GACTTGAAGAAGTCGTGCTGC

Supplementary Table S1 (continued). Primer sets used in this work

Purpose	Name	Forward primer (F; 5′ → 3′)	Reverse primer (R; 5′ → 3′)
qRT-PCR	qABCE1_F/R	CCTAAATCTTCGGAAAGTGAAC	GGCCATGAACCAACTTACGC
	qABCE2_F/R	GACAACTACCAAGAGAATATAGG	CAACTCAGGAAGTACAAAGCC
	qACTIN2_F/R[2]	GCACCCTGTTCTTCTTACCG	AACCCTCGTAGATTGGCACA
	OTC3D/OTCR[3]	TCCTTGCCAAATCATGGCCG	GCATGCATGCGATTCTCCGC
Gateway cloning	ABCE2pro:ABCE2_F/R	GGGGACAAGTTTGTACAAAAAAGCAGGCT TTTCTATCTTGTTATTCTTCGTTTTT	GGGGACCACTTTGTACAAGAAAGCTGGGT AGTTTTCCTATATCAGTGAGTTAG
	35Spro:ABCE2:GFP-YFP_F/R	GGGGACAAGTTTGTACAAAAAAGCAGGCT GGATGGCAGATCGATTGACACGTA	GGGGACCACTTTGTACAAGAAAGCTGGGT CATCATCCAAGTAGTAGTATGAGC
	ABCE1pro_F/R	GGGGACAAGTTTGTACAAAAAAGCAGGCT TACTTTTCTCTCGGCCGTACT	CAATACGTGTCAATCGATCTGCCATCTCTC TTGAGAATATTACATACAAG
	ABCE2tu_F/R	CTTGTATGTAATATTCTCAAGAGAGATGGC AGATCGATTGACACGTATTG	GGGGACCACTTTGTACAAGAAAGCTGGGT CTAATCATCCAAGTAGTAGTA
	ABCE2pro_F/R	GGGGACAAGTTTGTACAAAAAAGCAGGCT TTTCTATCTTGTTATTCTTCGTTTTT	AATCCGCGTCAATCGATCTGACATCTCTCA ACAGACCTACAAAATACATG
	ABCE1tu_F/R	CATGTATTTTGTAGGTCTGTTGAGAGATGT CAGATCGATTGACGCGGATT	GGGGACCACTTTGTACAAGAAAGCTGGGT TCAATCGTCTAAGTAGTAGTA

Sequences were taken from [1](S1), [2](S2), and [3](S3).

Supplementary Table S2. Excitation and detection parameters of fluorophores

Fluorophore	Laser type	Excitation (nm)	Detector type	Detection (nm)
GFP	Argon ion	488	Barrier filter	515/30
YFP				
VENUS				
DAPI	Diode	408	Barrier filter	450/35
Propidium iodide	Helium-neon	543	Barrier filter	605/75

Nuclei or cell walls were stained by immersing complete seedlings in a 0.2 $\mu g \cdot ml^{-1}$ DAPI solution (Sony Biotechnology) for 12 min or a 10 $\mu g \cdot ml^{-1}$ propidium iodide solution (Sigma-Aldrich) for 8 min, respectively.

Supplementary Table S3. Quality control summary of the RNA-seq assay

Sample	Number of clean reads	Q30 quality score (%)*	Mapped reads (%)
L*er* replicate 1	31536086	95.11	95.40
L*er* replicate 2	31336166	95.24	95.50
L*er* replicate 3	30756110	94.99	95.43
api7-1 replicate 1	29977410	94.95	96.14
api7-1 replicate 2	27789921	95.39	96.35
api7-1 replicate 3	29383285	94.36	95.95

*Q30 quality score indicates the percentage of bases whose correct base recognition rates are greater than 99.9% in total bases.

Supplementary Table S4. NCBI accession numbers of the sequences used for phylogenetic analysis

Species	*ABCE1* gene	*ABCE2* gene
Arabidopsis thaliana	NM_112210.3	ABCE2 NM_118041.5
Arabidopsis lyrata subsp. *lyrata*	XM_021033623.1	XM_021033596.1
Capsella rubella	XM_006299733.2	XM_006285967.2
Cardamine hirsuta	-	JX097073.1
Eutrema salsugineum	XM_006418662.2	XM_006413929.2
Brassica rapa (*a*)	XM_033286408.1	XM_033276765.1
Brassica rapa (*b*)	XM_009119375.3	XM_009138752.3
Brassica rapa (*c*)	-	XM_018655198.2
Fragaria vesca subsp. *vesca*	-	XM_004291176.2
Theobroma cacao	-	XM_018117748.1
Citrus sinensis	-	XM_015531558.2
Populus trichocarpa (*a*)	-	XM_024599960.1
Populus trichocarpa (*b*)	-	XM_024597900.1
Eschscholzia californica	-	Eca_sc194497.1_g0120.1*
Oryza sativa (*a*)	-	XM_015762126.2
Oryza sativa (*b*)	-	XM_026023394.1

Multiple *ABCE1* or *ABCE2* genes from *Brassica rapa*, *Populus trichocarpa*, and *Oryza sativa* are distinguished with arbitrarily given *a*, *b*, and *c* designations. *Obtained from Eschscholzia Genome DataBase (http://eschscholzia.kazusa.or.jp/cgi-bin/list.cgi).

Supplementary Table S5. Morphometry of the leaf venation pattern of the *api7-1* mutant

Organ	Genotype	Area (mm^2)	Circularity	Vein density	Vein branching points	Free-ending veins
Cotyledons	L*er*	2.9 ± 0.5	0.84 ± 0.03	2.7 ± 0.2	6.5 ± 0.5	2.1 ± 1.7
	api7-1	***1.9 ± 0.5***	0.85 ± 0.01	***3.1 ± 0.3***	*8.2 ± 2.4*	**4.4 ± 2.2**
First-node leaves	L*er*	33.9 ± 8.3	0.86 ± 0.02	3.1 ± 0.2	178.6 ± 35.6	77.8 ± 17.1
	api7-1	***11.2 ± 4.8***	***0.76 ± 0.05***	3.0 ± 0.3	***72.4 ± 24.6***	***35.9 ± 10.8***
Third-node leaves	L*er*	51.3 ± 11.2	0.85 ± 0.01	3.8 ± 0.3	361.7 ± 56.9	126.9 ± 24.4
	api7-1	***18.8 ± 4.0***	*0.84 ± 0.02*	*3.5 ± 0.4*	***145.2 ± 25.1***	***52.7 ± 10.6***
Cauline leaves	L*er*	200.6 ± 32.8	0.56 ± 0.23	3.9 ± 0.3	1307.6 ± 213.8	439.9 ± 66.1
	api7-1	***136.6 ± 50.5***	*0.69 ± 0.04*	4.2 ± 0.5	**1023.0 ± 201.1**	*373.3 ± 73.4*
Sepals	L*er*	1.4 ± 0.2	0.66 ± 0.05	6.6 ± 0.7	13.4 ± 4.2	9.5 ± 2.3
	api7-1	*1.3 ± 0.1*	0.65 ± 0.04	7.0 ± 1.0	14.7 ± 4.1	10.8 ± 4.8
Petals	L*er*	2.4 ± 0.4	0.70 ± 0.03	4.2 ± 0.6	6.4 ± 0.8	6.5 ± 1.8
	api7-1	2.2 ± 0.2	0.70 ± 0.03	4.0 ± 0.4	5.9 ± 1.1	7.7 ± 2.1

All values are means ± standard deviation from 12 measurements. Organs were collected 6 (cotyledons), 21 (first- and third-node leaves), and 35 (cauline leaves, petals, and sepals) das. Values in italics, bold, or bold and italics are significantly different from those of L*er* in a Student's *t* test, with $P < 0.05$, $P < 0.01$, or $P < 0.001$, respectively.

Supplementary Table S6. Mutations identified in the *api7-1* candidate interval

Mutation	Region affected	Predicted effect
G→A	At4g19185, 1st intron	-
G→A	At4g19185, 1st exon	Cys55→Cys (Synonymous)
C→T	At4g19210, 6th exon	Pro138→Ser
G→A	At4g19390, 2nd exon	Ile128→Ile (Synonymous)

Supplementary Table S7. ABCE2 interactors identified in a co-immunoprecipitation assay

AGI gene code	Protein		Peptides		Peptide coverage
	Abbreviation	Full name	Total[1]	Unique[2]	(%)
At4g19210	ABCE2	ATP-BINDING CASSETTE E2	153 (20)	26 (5)	62
At3g13640	ABCE1	ATP-BINDING CASSETTE E1			
-	YFP	Yellow fluorescent protein	59	10	-
At4g11420	eIF3a[3]	EUKARYOTIC TRANSLATION INITIATION FACTOR 3 SUBUNIT A	48	24	32
At3g56150	eIF3c[3]	EUKARYOTIC TRANSLATION INITIATION FACTOR 3 SUBUNIT C	29	11	14
At3g57290	eIF3e[3]	EUKARYOTIC TRANSLATION INITIATION FACTOR 3 SUBUNIT E	25	15	40
At1g64790	ILA[3]	ILITYHIA	17	11	4
At2g44060	LEA26[3]	LATE EMBRYOGENESIS ABUNDANT 26	15	6	23
At4g20980	eIF3d[3]	EUKARYOTIC TRANSLATION INITIATION FACTOR 3 SUBUNIT D	13 (8)	6 (4)	13
At5g44320	eIF3d[3]	EUKARYOTIC TRANSLATION INITIATION FACTOR 3 SUBUNIT D			
At5g17020	XPO1A[3]	EXPORTIN 1A	12 (4)	5 (2)	6
At3g03110	XPO1B[3]	EXPORTIN 1B			
At1g61580	RPL3B	RIBOSOMAL PROTEIN L3 B	12	4	12
At4g38740	ROC1	ROTAMASE CYP 1	11	5	38
At3g13460	ECT2[3]	EVOLUTIONARILY CONSERVED C-TERMINAL REGION 2	8	6	11
At4g33250	eIF3k[3]	EUKARYOTIC TRANSLATION INITIATION FACTOR 3 SUBUNIT K	8	4	23
At1g76810	eIF5B[3]	EUKARYOTIC TRANSLATION INITIATION FACTOR 5B	6	4	4
At3g53610	RAB8	RAB GTPASE HOMOLOG 8	6	3	18
At5g37475	eIF3j	EUKARYOTIC TRANSLATION INITIATION FACTOR 3 SUBUNIT J	5	3	16
At3g43600	AAO2	ALDEHYDE OXIDASE 2	4	3	3
At1g65860	FMO GS-OX1	FLAVIN-MONOOXYGENASE GLUCOSINOLATE S-OXYGENASE 1	4	3	4
At2g20830	-[4]	FOLIC ACID BINDING / TRANSFERASE	4	2	8
At5g58410	LTN1	E3 UBIQUITIN-PROTEIN LIGASE LISTERIN	4	2	1

Supplementary Table S7 (continued). ABCE2 interactors identified in a co-immunoprecipitation assay

AGI gene code	Protein		Peptides		Peptide coverage
	Abbreviation	Full name	Total[1]	Unique[2]	(%)
At2g42910	PRS4	PHOSPHORIBOSYL DIPHOSPHATE SYNTHASE 4	4	2	8
At3g08850	RAPTOR1	REGULATORY-ASSOCIATED PROTEIN OF TOR 1	4 (3)	2 (2)	1
At5g01770	RAPTOR2	REGULATORY-ASSOCIATED PROTEIN OF TOR 2			

Three biological replicates were assayed and we identified 20 candidate interactors. Translation initiation factors were named according to (S4). [1]Sum of the number of peptides identified in the three biological replicates. [2]Number of associated peptides with significantly different sequences from each other. Values within parentheses refer to the second protein of the paralogous group, whose peptides were also associated with the first protein in all cases. [3]Enriched proteins (identified with at least twice the number of peptides associated with the same protein in the control co-immunoprecipitations). The rest of the proteins were unique to ABCE2:YFP samples. [4]This protein was included after being first discarded due to its predicted mitochondrial localization.

Supplementary Table S8. Conservation level and described functions of putative ABCE2 interactors

AGI gene code	Protein	Conservation between orthologs (%)[1]			Described molecular function(s) and available evidence of its interaction with ABCE proteins[2]
		Arabidopsis and *S. cerevisiae*	Arabidopsis and *H. sapiens*	*S. cerevisiae* and *H. sapiens*	
At4g19210	ABCE2	68.9 (82.6)	75.4 (86.0)	68.3 (82.9)	In Arabidopsis: suppression of RNA silencing (S5,S6).<br>In yeast (Rli1): ribosome dissociation (S7).<br>In humans (ABCE1): inhibition of RNAse L, suppression of RNA silencing, and ribosome dissociation (S8,S9,S10).
At4g11420	eIF3a	26.3 (43.4)	25.7 (41.6)	18.3 (32.0)	In Arabidopsis, yeast (Rpg1), and humans (EIF3A): translation initiation (S11).
At3g56150	eIF3c	24.3 (41.7)	34.5 (49.9)	23.4 (39.2)	In Arabidopsis, yeast (Nip1), and humans (EIF3C): translation initiation (S11).
At4g20980	eIF3d	NC	39.0 (54.4)	NC	In Arabidopsis and humans (EIF3D): translation initiation (S11).
At3g57290	eIF3e	NC	50.1 (69.0)	NC	In Arabidopsis and humans (EIF3E): translation initiation (S11).
At5g37475	eIF3j	22.5 (37.0)	29.7 (45.1)	26.2 (40.9)	In yeast (also named High-Copy suppressor of Rpg1 [Hcr1]): translation initiation and, as a non-stoichiometric subunit of the eIF3 complex, participates in pre-40S maturation, and as an accessory factor for Rli1-mediated ribosome dissociation (S12,S13).<br>In humans (EIF3J): start codon selection and eIF3 complex formation during translation initiation (S14,S15). It is also present in the 40S post-splitting complex with ABCE1 (S16).
At4g33250	eIF3k	NC	31.4 (50.7)	NC	In Arabidopsis and humans (EIF3K): translation initiation (S11) .
At1g76810	eIF5B	36.3 (50.1)	39.7 (55.6)	36.5 (54.1)	In yeast (Fun12): 40S and 60S joining during pre-40S maturation and translation initiation (S17,S18,S19,S20).
At1g61580	RPL3B	65.3 (79.6)	64.9 (80.4)	65.3 (80.0)	In Arabidopsis, yeast (Rpl3), and humans (RPL3): ribosomal protein. Translation (S21,S22).

Supplementary Table S8 (continued). Conservation level and described functions of putative ABCE2 interactors

AGI code	Protein	Conservation between orthologs (%)[1]			Described molecular function(s) and available evidence of its interaction with ABCE proteins[2]
		Arabidopsis and *S. cerevisiae*	Arabidopsis and *H. sapiens*	*S. cerevisiae* and *H. sapiens*	
At1g64790	ILA	27.6 (45.1)	32.4 (50.2)	27.7 (47.1)	In Arabidopsis: translation regulation through two pathways, one involving GCN2 and eIF2α, and the other involving GCN20 (S23,S24).<br>In yeast (Gcn1): translation downregulation by binding to translating ribosomes with Gcn2 and Gcn20 (S25,S26).
At3g13460	ECT2	12.5 (19.6)	25.5 (35.9)	15.5 (24.6)	In Arabidopsis: m^{6}A reader that regulates 3′UTR processing in the nucleus and mRNA stability in the cytoplasm (S27,S28,S29).<br>In yeast (Pho92) and humans (YTHDF2): m^{6}A reader that decreases mRNA stability (S30,S31).
At5g58410	LTN1	19.2 (36.1)	22.5 (37.9)	20.8 (36.8)	In yeast, *Drosophila melanogaster*, and humans: ubiquitination of nascent non-stop proteins for their degradation during ribosome quality control (S32,S33,S34).
At4g38740	ROC1	64.0 (74.4)	67.4 (80.2)	64.2 (74.5)	In Arabidopsis, yeast (Cpr1), and humans (PPIA): it is a cyclophilin that belongs to the peptidyl-prolyl cis-trans isomerase family and participates in protein folding (S35,S36,S37).
At3g08850	RAPTOR1	27.3 (42.1)	40.4 (54.7)	31.8 (45.3)	In Arabidopsis, yeast (Kog1), and humans (RAPTOR): it is part of the TORC1 complex, composed of TOR, RAPTOR, and LST8-1 proteins. It controls cellular growth in response to different signals through regulation of translation, as it promotes translation reinitiation and ribosome biogenesis (S38,S39).<br>In Arabidopsis: ABCE2 has been shown to interact with LST8-1 (S40).
At2g20830	-	26.7 (47.2)	30.2 (43.4)	27.8 (50.9)	In Arabidopsis: not studied. A BLASTp search suggested homology to *S. cerevisiae* and human Lto1.<br>In yeast and humans: FeS cluster assembly on Rli1 and ABCE1, respectively (S41,S42).

Supplementary Table S8 (continued). Conservation level and described functions of putative ABCE2 interactors

AGI code	Protein	Conservation between orthologs (%)[1]			Described molecular function(s) and available evidence of its interaction with ABCE proteins[2]
		Arabidopsis and *S. cerevisiae*	Arabidopsis and *H. sapiens*	*S. cerevisiae* and *H. sapiens*	
At5g17020	XPO1A	40.7 (61.7)	48.5 (67.6)	46.2 (65.8)	In Arabidopsis, yeast (Crm1), and humans (XPO1): nuclear export receptor (S43,S44).<br>In yeast, humans, and *Xenopus laevis*: ABCE might be an XPO1 cargo (S45).<br>In yeast: *xpo1-1* mutants accumulate ABCE1 in nucleus (S46,S47).
At2g42910	PRS4	20.7 (38.2)	19.2 (40.0)	60.3 (76.6)	In yeast (Prs4) and humans (PRPS1): synthesis of phosphoribosylpyrophosphate (PRPP), which is required for nucleotide biosynthesis (S48).
At1g65860	FMO GS-OX1	NC	NC	NC	In Arabidopsis: synthesis of aliphatic glucosinolates (S49).
At3g53610	RAB8	51.1 (70.0)	58.7 (73.1)	48.9 (67.0)	In Arabidopsis: it might be involved in post-Golgi transport to the plasma membrane (S50,S51).<br>In yeast (Sec4): involved in membrane trafficking during cytokinesis and autophagy (S52,S53).
At3g43600	AAO2	NC	29.6 (48.1)	NC	In Arabidopsis: it might be involved in ABA biosynthesis (S54,S55).<br>In humans (AOX1): it is an oxidase with broad substrate specificity (S56).
At2g44060	LEA26	NC	NC	NC	In Arabidopsis: unknown.

[1]Identity and similarity (between parentheses) percentages were obtained by global pairwise sequence alignments between pairs of protein sequences using the Needle EMBOSS tool. Protein sequences were obtained from TAIR for Arabidopsis, *Saccharomyces* Genome Database (SGD; https://www.yeastgenome.org/) for *S. cerevisiae*, and UniProt for *H. sapiens* proteins. NC: not conserved. [2]The abbreviated names for *S. cerevisiae* and *H. sapiens* orthologs are indicated in parentheses. The full names of Arabidopsis proteins are provided in Supplementary Table S7.

SUPPLEMENTARY REFERENCES

S1. Kleinboelting, N., Huep, G., Kloetgen, A., Viehoever, P. and Weisshaar, B. (2012) GABI-Kat SimpleSearch: new features of the *Arabidopsis thaliana* T-DNA mutant database. *Nucleic Acids Res.*, **40**, D1211-1215.

S2. Moschopoulos, A., Derbyshire, P. and Byrne, M.E. (2012) The *Arabidopsis* organelle-localized glycyl-tRNA synthetase encoded by *EMBRYO DEFECTIVE DEVELOPMENT1* is required for organ patterning. *J. Exp. Bot.*, **63**, 5233-5243.

S3. Quesada, V., Ponce, M.R. and Micol, J.L. (1999) *OTC* and *AUL1*, two convergent and overlapping genes in the nuclear genome of *Arabidopsis thaliana*. *FEBS Lett.*, **461**, 101-106.

S4. Browning, K.S. and Bailey-Serres, J. (2015) Mechanism of cytoplasmic mRNA translation. *The Arabidopsis Book*, **13**, e0176.

S5. Mõttus, J., Maiste, S., Eek, P., Truve, E. and Sarmiento, C. (2020) Mutational analysis of *Arabidopsis thaliana* ABCE2 identifies important motifs for its RNA silencing suppressor function. *Plant Biol.*, **23**, 21-31.

S6. Sarmiento, C., Nigul, L., Kazantseva, J., Buschmann, M. and Truve, E. (2006) AtRLI2 is an endogenous suppressor of RNA silencing. *Plant Mol. Biol.*, **61**, 153-163.

S7. Shoemaker, C.J. and Green, R. (2011) Kinetic analysis reveals the ordered coupling of translation termination and ribosome recycling in yeast. *Proc. Natl. Acad. Sci. USA*, **108**, E1392-E1398.

S8. Kärblane, K., Gerassimenko, J., Nigul, L., Piirsoo, A., Smialowska, A., Vinkel, K., Kylsten, P., Ekwall, K., Swoboda, P., Truve, E. *et al.* (2015) ABCE1 is a highly conserved RNA silencing suppressor. *PLOS ONE*, **10**, e0116702.

S9. Pisarev, A.V., Skabkin, M.A., Pisareva, V.P., Skabkina, O.V., Rakotondrafara, A.M., Hentze, M.W., Hellen, C.U. and Pestova, T.V. (2010) The role of ABCE1 in eukaryotic posttermination ribosomal recycling. *Mol. Cell*, **37**, 196-210.

S10. Bisbal, C., Martinand, C., Silhol, M., Lebleu, B. and Salehzada, T. (1995) Cloning and characterization of a RNase L inhibitor. A new component of the interferon-regulated 2-5A pathway. *J. Biol. Chem.*, **270**, 13308-13317.

S11. Burks, E.A., Bezerra, P.P., Le, H., Gallie, D.R. and Browning, K.S. (2001) Plant initiation factor 3 subunit composition resembles mammalian initiation factor 3 and has a novel subunit. *J. Biol. Chem.*, **276**, 2122-2131.

S12. Valášek, L., Hašek, J., Nielsen, K.H. and Hinnebusch, A.G. (2001) Dual function of eIF3j/Hcr1p in processing 20 S pre-rRNA and translation initiation. *J. Biol. Chem.*, **276**, 43351-43360.

S13. Young, D.J. and Guydosh, N.R. (2019) Hcr1/eIF3j is a 60S ribosomal subunit recycling accessory factor *in vivo*. *Cell. Rep.*, **28**, 39-50.

S14. Borgo, C., Franchin, C., Salizzato, V., Cesaro, L., Arrigoni, G., Matricardi, L., Pinna, L.A. and Donella-Deana, A. (2015) Protein kinase CK2 potentiates translation efficiency by phosphorylating eIF3j at Ser127. *Biochim. Biophys. Acta*, **1853**, 1693-1701.

S15. ElAntak, L., Wagner, S., Herrmannová, A., Karásková, M., Rutkai, E., Lukavsky, P.J. and Valášek, L. (2010) The indispensable N-terminal half of eIF3j/HCR1 cooperates with its structurally conserved binding partner eIF3b/PRT1-RRM and with eIF1A in stringent AUG selection. *J. Mol. Biol.*, **396**, 1097-1116.

S16. Kratzat, H., Mackens-Kiani, T., Ameismeier, M., Potocnjak, M., Cheng, J., Dacheux, E., Namane, A., Berninghausen, O., Herzog, F., Fromont-Racine, M. *et al.* (2021) A structural inventory of native ribosomal ABCE1-43S pre-initiation complexes. *EMBO J.*, **40**, e105179.

S17. Fringer, J.M., Acker, M.G., Fekete, C.A., Lorsch, J.R. and Dever, T.E. (2007) Coupled release of eukaryotic translation initiation factors 5B and 1A from 80S ribosomes following subunit joining. *Mol. Cell. Biol.*, **27**, 2384-2397.

S18. Lebaron, S., Schneider, C., van Nues, R.W., Swiatkowska, A., Walsh, D., Böttcher, B., Granneman, S., Watkins, N.J. and Tollervey, D. (2012) Proofreading of pre-40S ribosome maturation by a translation initiation factor and 60S subunits. *Nat. Struct. Mol. Biol.*, **19**, 744-753.

S19. Strunk, B.S., Novak, M.N., Young, C.L. and Karbstein, K. (2012) A translation-like cycle is a quality control checkpoint for maturing 40S ribosome subunits. *Cell*, **150**, 111-121.

S20. Wang, J., Johnson, A.G., Lapointe, C.P., Choi, J., Prabhakar, A., Chen, D.H., Petrov, A.N. and Puglisi, J.D. (2019) eIF5B gates the transition from translation initiation to elongation. *Nature*, **573**, 605-608.

S21. García-Gómez, J.J., Fernández-Pevida, A., Lebaron, S., Rosado, I.V., Tollervey, D., Kressler, D. and de la Cruz, J. (2014) Final pre-40S maturation depends on the functional integrity of the 60S subunit ribosomal protein L3. *PLOS Genet.*, **10**, e1004205.

S22. Meskauskas, A. and Dinman, J.D. (2007) Ribosomal protein L3: gatekeeper to the A-site. *Mol. Cell*, **25**, 877-888.

S23. Faus, I., Niñoles, R., Kesari, V., Llabata, P., Tam, E., Nebauer, S.G., Santiago, J., Hauser, M.T. and Gadea, J. (2018) Arabidopsis ILITHYIA protein is necessary for proper chloroplast biogenesis and root development independent of eIF2α phosphorylation. *J. Plant Physiol.*, **224-225**, 173-182.

S24. Izquierdo, Y., Kulasekaran, S., Benito, P., López, B., Marcos, R., Cascón, T., Hamberg, M. and Castresana, C. (2018) Arabidopsis *nonresponding to oxylipins* locus *NOXY7* encodes a yeast GCN1 homolog that mediates noncanonical translation regulation and stress adaptation. *Plant Cell Environ.*, **41**, 1438-1452.

S25. Lee, S.J., Swanson, M.J. and Sattlegger, E. (2015) Gcn1 contacts the small ribosomal protein Rps10, which is required for full activation of the protein kinase Gcn2. *Biochem. J.*, **466**, 547-559.

S26. Sattlegger, E. and Hinnebusch, A.G. (2005) Polyribosome binding by GCN1 is required for full activation of eukaryotic translation initiation factor 2α kinase GCN2 during amino acid starvation. *J. Biol. Chem.*, **280**, 16514-16521.

S27. Arribas-Hernández, L., Bressendorff, S., Hansen, M.H., Poulsen, C., Erdmann, S. and Brodersen, P. (2018) An m^{6}A-YTH module controls developmental timing and morphogenesis in Arabidopsis. *Plant Cell*, **30**, 952-967.

S28. Scutenaire, J., Deragon, J.M., Jean, V., Benhamed, M., Raynaud, C., Favory, J.J., Merret, R. and Bousquet-Antonelli, C. (2018) The YTH domain protein ECT2 is an m^{6}A reader required for normal trichome branching in Arabidopsis. *Plant Cell*, **30**, 986-1005.

S29. Wei, L.H., Song, P., Wang, Y., Lu, Z., Tang, Q., Yu, Q., Xiao, Y., Zhang, X., Duan, H.C. and Jia, G. (2018) The m^{6}A reader ECT2 controls trichome morphology by affecting mRNA stability in Arabidopsis. *Plant Cell*, **30**, 968-985.

S30. Kang, H.J., Jeong, S.J., Kim, K.N., Baek, I.J., Chang, M., Kang, C.M., Park, Y.S. and Yun, C.W. (2014) A novel protein, Pho92, has a conserved YTH domain and regulates phosphate metabolism by decreasing the mRNA stability of *PHO4* in *Saccharomyces cerevisiae*. *Biochem. J.*, **457**, 391-400.

S31. Wang, X., Lu, Z., Gomez, A., Hon, G.C., Yue, Y., Han, D., Fu, Y., Parisien, M., Dai, Q., Jia, G. *et al.* (2014) N^6-methyladenosine-dependent regulation of messenger RNA stability. *Nature*, **505**, 117-120.

S32. Bengtson, M.H. and Joazeiro, C.A. (2010) Role of a ribosome–associated E3 ubiquitin ligase in protein quality control. *Nature*, **467**, 470-473.

S33. Kashima, I., Takahashi, M., Hashimoto, Y., Sakota, E., Nakamura, Y. and Inada, T. (2014) A functional involvement of ABCE1, eukaryotic ribosome recycling factor, in nonstop mRNA decay in *Drosophila melanogaster* cells. *Biochimie*, **106**, 10-16.

S34. Shao, S., von der Malsburg, K. and Hegde, R.S. (2013) Listerin-dependent nascent protein ubiquitination relies on ribosome subunit dissociation. *Mol. Cell*, **50**, 637-648.

S35. Coaker, G., Zhu, G., Ding, Z., Van Doren, S.R. and Staskawicz, B. (2006) Eukaryotic cyclophilin as a molecular switch for effector activation. *Mol. Microbiol.*, **61**, 1485-1496.

S36. Davis, T.L., Walker, J.R., Campagna-Slater, V., Finerty, P.J., Paramanathan, R., Bernstein, G., MacKenzie, F., Tempel, W., Ouyang, H., Lee, W.H. *et al.* (2010) Structural and biochemical characterization of the human cyclophilin family of peptidyl-prolyl isomerases. *PLOS Biol.*, **8**, e1000439.

S37. Haendler, B., Keller, R., Hiestand, P.C., Kocher, H.P., Wegmann, G. and Movva, N.R. (1989) Yeast cyclophilin: isolation and characterization of the protein, cDNA and gene. *Gene*, **83**, 39-46.

S38. Kim, D.H. and Sabatini, D.M. (2004) Raptor and mTOR: subunits of a nutrient-sensitive complex. *Curr. Top. Microbiol. Immunol.*, **279**, 259-270.

S39. Schepetilnikov, M., Dimitrova, M., Mancera-Martínez, E., Geldreich, A., Keller, M. and Ryabova, L.A. (2013) TOR and S6K1 promote translation reinitiation of uORF-containing mRNAs via phosphorylation of eIF3h. *EMBO J.*, **32**, 1087-1102.

S40. Van Leene, J., Han, C., Gadeyne, A., Eeckhout, D., Matthijs, C., Cannoot, B., De Winne, N., Persiau, G., Van De Slijke, E., Van de Cotte, B. *et al.* (2019) Capturing the phosphorylation and protein interaction landscape of the plant TOR kinase. *Nat. Plants*, **5**, 316-327.

S41. Paul, V.D., Mühlenhoff, U., Stümpfig, M., Seebacher, J., Kugler, K.G., Renicke, C., Taxis, C., Gavin, A.C., Pierik, A.J. and Lill, R. (2015) The deca-GX_3 proteins Yae1-Lto1 function as adaptors recruiting the ABC protein Rli1 for iron-sulfur cluster insertion. *eLIFE*, **4**, e08231.

S42. Zhai, C., Li, Y., Mascarenhas, C., Lin, Q., Li, K., Vyrides, I., Grant, C.M. and Panaretou, B. (2014) The function of ORAOV1/LTO1, a gene that is overexpressed frequently in cancer: essential roles in the function and biogenesis of the ribosome. *Oncogene*, **33**, 484-494.

S43. Xu, X., Wan, W., Jiang, G., Xi, Y., Huang, H., Cai, J., Chang, Y., Duan, C.G., Mangrauthia, S.K., Peng, X. *et al.* (2019) Nucleocytoplasmic trafficking of the *Arabidopsis* WD40 repeat protein XIW1 regulates ABI5 stability and abscisic acid responses. *Mol. Plant*, **12**, 1598-1611.

S44. Zhu, G., Chang, Y., Xu, X., Tang, K., Chen, C., Lei, M., Zhu, J.K. and Duan, C.G. (2019) EXPORTIN 1A prevents transgene silencing in *Arabidopsis* by modulating nucleo-cytoplasmic partitioning of HDA6. *J. Integr. Plant Biol.*, **61**, 1243-1254.

S45. Kirli, K., Karaca, S., Dehne, H.J., Samwer, M., Pan, K.T., Lenz, C., Urlaub, H. and Görlich, D. (2015) A deep proteomics perspective on CRM1-mediated nuclear export and nucleocytoplasmic partitioning. *eLIFE*, **4**, e11466.

S46. Kispal, G., Sipos, K., Lange, H., Fekete, Z., Bedekovics, T., Janáky, T., Bassler, J., Aguilar Netz, D.J., Balk, J., Rotte, C. *et al.* (2005) Biogenesis of cytosolic ribosomes requires the essential iron-sulphur protein Rli1p and mitochondria. *EMBO J.*, **24**, 589-598.

S47. Yarunin, A., Panse, V.G., Petfalski, E., Dez, C., Tollervey, D. and Hurt, E.C. (2005) Functional link between ribosome formation and biogenesis of iron-sulfur proteins. *EMBO J.*, **24**, 580-588.

S48. Hernando, Y., Carter, A.T., Parr, A., Hove-Jensen, B. and Schweizer, M. (1999) Genetic analysis and enzyme activity suggest the existence of more than one minimal functional unit capable of synthesizing phosphoribosyl pyrophosphate in *Saccharomyces cerevisiae*. *J. Biol. Chem.*, **274**, 12480-12487.

S49. Hansen, B.G., Kliebenstein, D.J. and Halkier, B.A. (2007) Identification of a flavin-monooxygenase as the *S*-oxygenating enzyme in aliphatic glucosinolate biosynthesis in Arabidopsis. *Plant J.*, **50**, 902-910.

S50. Rutherford, S. and Moore, I. (2002) The *Arabidopsis* Rab GTPase family: another enigma variation. *Curr. Opin. Plant Biol.*, **5**, 518-528.

S51. Speth, E.B., Imboden, L., Hauck, P. and He, S.Y. (2009) Subcellular localization and functional analysis of the Arabidopsis GTPase RabE. *Plant Physiol.*, **149**, 1824-1837.

S52. Geng, J., Nair, U., Yasumura-Yorimitsu, K. and Klionsky, D.J. (2010) Post-Golgi Sec proteins are required for autophagy in *Saccharomyces cerevisiae*. *Mol. Biol. Cell*, **21**, 2257-2269.

S53. Lepore, D., Spassibojko, O., Pinto, G. and Collins, R.N. (2016) Cell cycle–dependent phosphorylation of Sec4p controls membrane deposition during cytokinesis. *J. Cell Biol.*, **214**, 691-703.

S54. Khan, M., Imran, Q.M., Shahid, M., Mun, B.G., Lee, S.U., Khan, M.A., Hussain, A., Lee, I.J. and Yun, B.W. (2019) Nitric oxide- induced *AtAO3* differentially regulates plant defense and drought tolerance in *Arabidopsis thaliana*. *BMC Plant Biol.*, **19**, 602.

S55. Seo, M., Aoki, H., Koiwai, H., Kamiya, Y., Nambara, E. and Koshiba, T. (2004) Comparative studies on the *Arabidopsis* aldehyde oxidase (*AAO*) gene family revealed a major role of *AAO3* in ABA biosynthesis in seeds. *Plant Cell Physiol.*, **45**, 1694-1703.

S56. Cheshmazar, N., Dastmalchi, S., Terao, M., Garattini, E. and Hamzeh-Mivehroud, M. (2019) Aldehyde oxidase at the crossroad of metabolism and preclinical screening. *Drug Metab. Rev.*, **51**, 428-452.
